# From biomolecules to breakthroughs: exosomes as next-generation theranostics in female infertility

**DOI:** 10.3389/fcell.2025.1605174

**Published:** 2025-10-01

**Authors:** Ahmed A. Aldarmahi, Shifan Khanday, Ehab S. Taher, Ahmed Abdeen, Gamal A. Atia, Dania A. Mohammed, Dina S. Nasr, Rayan G. Albarakati, Donia E. Zaghamir, Helal F. Hetta, Ahmed M. Atwa, Kasim S. Abass, Ekramy M. Elmorsy, Abeer Alshambky, Mohamed A. El-Sakhawy, Ali El-Far, Shimaa S. Attia

**Affiliations:** ^1^ Department of Basic Science, College of Science and Health Professions, King Saud bin Abdulaziz University for Health Sciences, Jeddah, Saudi Arabia; ^2^ National Guard- Health Affairs, King Abdullah International Medical Research Centre, Jeddah, Saudi Arabia; ^3^ Department of Biomedical Sciences, Dubai Medical College for Girls, Dubai Medical University, Dubai, United Arab Emirates; ^4^ Department of Basic and Clinical Medical Sciences, Faculty of Dentistry, Zarqa University, Zarqa, Jordan; ^5^ Department of Forensic Medicine and Toxicology, Faculty of Veterinary Medicine, Benha University, Toukh, Egypt; ^6^ Department of Oral Medicine, Periodontology, and Diagnosis, Faculty of Dentistry, Suez Canal University, Ismailia, Egypt; ^7^ Department of Physiology, Faculty of Medicine, Benha University, Benha, Egypt; ^8^ Department of Clinical Medical Sciences, College of Medicine, AlMaarefa University, Riyadh, Saudi Arabia; ^9^ Research Center, Deanship of Scientific Research and Post-Graduate Studies, Al Maarefa University, Riyadh, Saudi Arabia; ^10^ College of Nursing, Prince Sattam bin Abdualziz University, Alkarj, Saudi Arabia; ^11^ Division of Microbiology, Immunology and Biotechnology, Department of Natural Products and Alternative Medicine, Faculty of Pharmacy, University of Tabuk, Tabuk, Saudi Arabia; ^12^ Department of Pharmacology and Toxicology, Faculty of Pharmacy, Egyptian Russian University, Cairo, Egypt; ^13^ College of Pharmacy, Al-Ayen Iraqi University, AUIQ, An Nasiriyah, Iraq; ^14^ College of Veterinary Medicine, University of Kirkuk, Kirkuk, Iraq; ^15^ Center for Health Research, Northern Border University, Arar, Saudi Arabia; ^16^ Department of Forensic Medicine and Clinical Toxicology, Faculty of Medicine, Mansoura University, Mansoura, Egypt; ^17^ Center for metabolic disease research, Lewis Katz School of Medicine, Temple University, Philadelphia, PA, United States; ^18^ Department of Biochemistry, Animal Health Research Institute, Cairo, Egypt; ^19^ Department of Medical Laboratory, College of Applied Medical Sciences, Prince Sattam bin Abdulaziz University, Al-Kharj, Saudi Arabia; ^20^ Department of Medicinal and Aromatic Plants, Desert Research Center, Cairo, Egypt; ^21^ Key Laboratory of Epigenetics and Oncology, The Research Center for Preclinical Medicine, Southwest Medical University, Luzhou, China; ^22^ Department of Anatomy and Embryology, Faculty of Medicine, Ain-Shams University, Cairo, Egypt; ^23^ Department of Biomedical Sciences, College of Medicine, Gulf Medical University, Ajman, United Arab Emirates

**Keywords:** nanomedicine, gynecological cancers, extracellular vesicles, stem cell, tissue engineering, regenerative medicine

## Abstract

Female infertility and reproductive disorders represent a significant global health challenge, with complex etiologies often linked to impaired cellular communication, inflammation, and tissue dysfunction. Exosomes (EXOs), nanosized extracellular vesicles laden with bioactive molecules, have become recognized as significant transmitters of intercellular signaling in reproductive physiology and pathology. This review comprehensively discusses the dual diagnostic and therapeutic potential of EXOs in addressing female infertility disorders, such as endometriosis, polycystic ovary syndrome (PCOS), primary ovarian insufficiency (POI), Asherman syndrome, and gynecological cancers. We investigate the strategies whereby EXOs govern important activities like endometrial regeneration, folliculogenesis, immune modulation, and angiogenesis, while highlighting their role in restoring ovarian and uterine homeostasis. Advances in exosome isolation techniques, bioengineering strategies (e.g., cargo loading, surface modification), and scaffold-based delivery systems are critically evaluated for their capacity to enhance therapeutic precision and efficacy. Notwithstanding their potential, issues include standardization of isolation protocols, scalability, and long-term safety, which necessitate further research. By integrating molecular insights with translational innovations, this review underscores the clinical implementation of exosome-based therapeutics in revolutionizing reproductive medicine, offering new hope for personalized, non-invasive treatments in female fertility restoration.

## 1 Introduction

Infertility can be described as the inability of a woman to get pregnant following at least 1 year of periodic, unprotected sexual activity (Ara et al., 2022). Infertility is caused by a variety of clinical disorders, anatomical malformations, and ecological and genetic variables, which makes it a complex condition (Bala et al., 2021). Despite the presence of several variables that contribute to infertility conditions, female related infertility issues are the most common factors (Alesi et al., 2024). These illnesses involve ovulatory dysfunction, ovarian cancer, and endometrial disorders (Bhardwaj et al., 2021). While female infertility impacts millions of women throughout the world, there have been few recent advancements in theranostics (Wang et al., 2024c). During implantation, embryo-maternal crosstalk takes place, and structural and functional alteration of the endometrium and uterine space result in an optimal embryo implantation (Andreescu, 2023). Any disruption in any of the above procedures might result in infertility (Berdiaki et al., 2024).

Tissue engineering uses a mix of cells, biomaterials, and engineering technologies to fix and substitute damaged tissues, as well as preserve and restore the functionality of tissues following injury (Taymour et al., 2024). It provides a unique approach for prompt therapy and repair that aims to enhance long-lasting outcomes (Han and Du, 2020). Stem cell-based treatments have developed as the major method because of their distinctive capabilities of self-renewal and transformation (Jahanbani et al., 2020). Nevertheless, allogenic cells, often utilized in regenerative therapies, have intrinsic limits and obstacles, including immunological reactivity, tumorigenic potentials, and ethical issues. While endogenous cells provide a safer and more affordable alternative, they have diminished cellular metabolism and functionality in old or sick individuals (Pourakbari et al., 2020). As a result, maintaining endogenous cell activity is critical for improving tissue regeneration effectiveness in aged or sick populations. Cells release numerous extracellular vesicles (EVs) under both healthy and pathological situations, as components of their regular functioning after acquired disorders (Cervelló et al., 2015). EVs may be characterized depending on their biogenetic process, physical properties, and composition (Miron and Zhang, 2024). EVs are typically classified into three types depending on their biosynthesis and dimensions: microvesicles, EXOs, and apoptosomes (Lu et al., 2021a).

EXOs have been extracted from numerous tissues and fluids. EXOs are currently gaining popularity as a viable possibility for repairing and increasing endogenous cell activity while also facilitating tissue healing (Li et al., 2024c). EXOs are essential transmitters of paracrine signals and transport a variety of bioactive cargos. As cell-to-cell transmitters, their contents transfer commands from original cells to targeted cells, effectively controlling physiological processes such as immunological reactions, aging, neural communication, inflammatory processes, and disease promotion/inhibition (Rodríguez-Eguren et al., 2022). Their function in intercellular communication is complicated by the unique and little-understood processes and procedures of exosome intake by receptor cells. Following absorption, EXOs are either broken down by lysosomes or join with the endosomal membrane to expel EXOs cargos into the cytoplasm (Patel et al., 2021). However, EXOS can be returned to the plasma membrane and re-secreted outside of cells. After being consumed by endogenous cells and reversing their pathogenic changes, natural or synthetic EXOs interacting with them may offer therapeutic benefits (Si et al., 2023).

Furthermore, compared to standard cell treatment, EXOs have lower immunogenicity, improved storage and distribution stability, and less ethical debate (Kim et al., 2022a; Zhu et al., 2024). The present article aims to offer insights into the functioning of EXOs and how they can be enhanced to improve endogenous cell activity in the management of different types of female infertility disorders.

## 2 Mechanism of actions of exosomes in pathophysiological conditions

### 2.1 EXO cargo and intercellular communication

Cell metabolites are secreted into the surrounding environment by diffusion, membrane channels, and active secretion. EXOs have been identified to facilitate the transport of metabolites or across cells (Desdín-Micó et al., 2017). EXOs have been discovered as transmitters of signals between cells in both normal and pathologic conditions (Bowers et al., 2020). EXOs can carry RNA, proteins, enzymes, and lipids, influencing numerous biological mechanisms in numerous disorders such as cancer, neurological diseases, infections, and autoimmune diseases. Consequently, they serve vital functions in numerous biological processes, like angiogenic activities, antigen presentation, apoptosis, coagulation, cellular equilibrium, inflammatory processes, and interactions between cells (Zhang et al., 2023d).

EXOs can transport misfolded proteins, prions, and neurotoxic proteins, such as amyloid β (Shetgaonkar et al., 2022). EXOs may transport building materials between cells, like amino acids and lipids, and transport them to numerous locations throughout the body (O’Brien et al., 2020). These structures also influence the uterine microenvironment by enhancing the bioavailability of chemicals for energy synthesis. EXOs are thought to engage with endocrine and paracrine systems that regulate homeostasis (Xiong et al., 2023).

### 2.2 EXOs in immune regulation and inflammation

EXOs miRNA release provides a quick way to control gene expression. EXOs release miR-23 and miR-182 when muscles are forced to atrophy. The discharge of these cargoes may alleviate cellular stress (Zhang et al., 2022b). EXOs also have a role in blood vessel development, cellular transformation, immune regulation, metabolism, the elimination of outdated molecules, and antigen presentation (Ahmadieh-Yazdi et al., 2024). Trophoblast cells secrete EXOs that can control angiogenesis, or placenta development, via the matrix metalloproteinase inducer (EMMPRIM) (Göhner et al., 2017).

Progenitor cells can produce EXOs that play an essential role in the movement of endothelial cells in blood arteries, as well as cell division and angiogenesis (Liang et al., 2016). Exosome content during inflammatory processes may serve as a novel biomarker for inflammatory illnesses and disorders (Chen et al., 2021; Umair et al., 2022).

Neoplastic EXOs have been reported to contain an elevated miRNA content, suppress T-cell division and transformation, and trigger apoptosis via the FasL and MARK1 pathways, hence enhancing the tumor’s ability to fight against immune-mediated responses (Bernardi and Farina, 2021).

Fabbri et al. discovered that cancer miR-21 and miR-29a-loaded EXOs trigger inflammation and cytokine discharge by attaching to the Toll-like 8 receptor and activating it in immunological cells, resulting in NF-κB activity (Fabbri et al., 2012).

### 2.3 EXOs in cancer and aging

The role of the senescence-associated secretory phenotype (SASP) in aging is intricate and multidimensional. Although it can aid in tissue healing and the immune system’s removal of damaged cells, its ongoing activation leads to age-related illnesses and chronic inflammation. With regard to the situation, the SASP, a group of substances released by senescent cells, may possess both positive and negative consequences (Tanaka and Takahashi, 2021).

EXOs contain an abundance of the Wnt system, which is imperative for the maintenance of physiological equilibrium and performs functions in aging. Consequently, EXOs are thought to function as SASP messengers. They get liberated by aged fibroblasts and epithelial cells. Composition of EXOs from elderly individuals is altered; for example, levels of galectin-3, important for bone cell development, are significantly reduced (D’Anca et al., 2019).

In a healthy person, these mechanisms are controlled to perform the changes essential for healthy reproductive adjustments to take place. As a result of their actions in cell signaling and modulation at various stages of the reproductive cycle, EXOs play a crucial part in preserving a regulated reproductive condition (Tong et al., 2016; Fazeli and Godakumara, 2024). Grange et al. discovered that microvesicles from tumors have a role in metastasis and angiogenesis. Human kidney cancer has a subpopulation of cells that express the CD105 antigen, which is a hallmark of mesenchymal stem cells (MSCs). Furthermore, CD105-positive cells possess the capability to change the tumor’s surrounding environment and induce revascularization (Grange et al., 2011).

### 2.4 EXOs in reproductive physiology

EXOs perform several functions during the reproductive cycle by activating multiple regulatory processes triggered by fetal-maternal communications and cellular control (Natali et al., 2023), therefore giving the body adaptation capacities for a variety of physiological alterations (Morales-Prieto et al., 2020). These pathways may be linked to immune responses, signals of inflammation, and metabolic adjustments required to nurture a developing fetus (Chiarello et al., 2018).

## 3 Origin and separation of exosomes

In the realm of exosome investigation, several methodologies have been used to isolate and purify them such as ultracentrifugation, precipitation, immunoaffinity capture, and microfluidic approaches (Lin et al., 2021a). These methods offer advantages such as high yield and accessibility (Johnstone, 2020). Mammalian-derived EXOs are commonly extracted from physiological secretions, whereas plant EXOs are obtained from apoplastic washing solution, with differential centrifugation remaining the primary extraction technique for both (Stanly et al., 2016).

Scientists have developed numerous centrifugation technique combinations to boost the efficiency of separation and solve some of the limitations of traditional centrifugation procedures. One extensively used technique mixes differential centrifugation with sucrose density gradient centrifugation (Kim et al., 2022b). This approach is widespread because it is simple to use, inexpensive, and capable of producing exceptional extracting efficiency. Employing this method, EXOs frequently stay in the intermediary layer of a 30%–45% sucrose solution. In addition, there are various other extraction strategies, like Immunoaffinity capture, ultra-filtering or size-exclusion chromatography (SEC), co-precipitation methods, and microfluidic advancements, that have been effectively utilized in the case of mammalian EXOs (Le Gall et al., 2020). For example, immunoaffinity capture, which involves the creation of immunological systems that target extracellular vesicle surface antigens, has features such as quick separation, straightforwardness, and excellent specificity, rendering it a suitable approach for the purification of extracellular vesicles (Song et al., 2020).

Furthermore, various developing methodologies have made effective EXOs separation in trials possible, making future studies and applications more convenient. It is worth noting that there is far more variety between plants than between animals, resulting in considerable changes in dimensions, shape, productivity, purity, and dispersion of EXOs generated by various cells. Furthermore, the composition of EXOs derived from diverse sources varies significantly. Precipitation kits/polymer and ultracentrifugation are two typical ways of obtaining EXOs. These procedures can give solutions with outstanding recovery percentages, but limited specificity (Hendijani, 2017). Size filtration and flow cytometry can be used to extract EXOs with a high degree of specificity. Meanwhile, the last two procedures have inadequate recuperation rates and are susceptible to damaging vesicle architecture; hence, they are seldom used. EXOs are tiny and heterogeneous, and the number of carriers transported inside an individual exosome is limited. Abundant inactive components in Exos may diminish therapeutic effectiveness and raise therapeutic dosage (Xin et al., 2020).

Polymer precipitating methods may differentiate EXOs from bodily fluids by reducing their ability to dissolve. The combination of ultracentrifugation and the ExoQuick polymer precipitation technology enhances the integrity and extraction efficiency of plant EXOs, particularly those derived from ginseng (Jokhio et al., 2024).

Musante et al. proposed a strategy for isolating EXOs from urine samples using hydrostatic filtration dialysis. The most significant benefits of this technology are the elimination of the ultracentrifugation stage and the ability to isolate EXOs from much diluted liquids. Furthermore, the authors ensured that this strategy prevents EXOs’ loss (Musante et al., 2014). The specimens were centrifuged at 2,000 × g to exclude any contaminants and some of the Tamm–Horsfall protein (THP) aggregates. The resulting liquid is passed to a separator with a dialysis membrane that can permeate particulates up to 1,000 kDa. This procedure removes undesired ingredients from the specimen and reduces its volume. They centrifuged the EXOs at 40,000 × g to sediment them. The authors successfully isolated EXOs measuring 50–90 nm and containing a EXOs biomarker, TSG101. This approach combines the quantity, volume, and electrolytic content of the sample; thus, the researchers recommend it to handle specimens designated for preservation in biobanks (Barreiro et al., 2020). This procedure is essentially ultrafiltration in situations where the sample is subjected to a modest amount of pressure, similar to the fluid column in a dialysis unit (Musante, 2024).

Kim et al. described an innovative strategy for EXOs separation using a two-stage process with ATPS, which is presented to solve the issue of protein infiltration in the EXOs portion. Under specific conditions, these two macromolecules dissolve simultaneously in aqueous solution and generate two distinct phases. In this procedure, tailored biochemical properties of the chemical reactions between polymer molecules and EXOs trigger the latter to accumulate preferentially in the dextran (DEX) phase. In contrast, other ingredients traveled between the phases, accumulating preferentially in the polyethylene glycol (PEG) phase. This study established a straightforward and rapid separation procedure from a tiny sample volume utilizing a PEG/DEX ATPS that did not require any specific equipment. The ATPS isolation approach demonstrated a sevenfold greater recovery performance than the standard ultra-centrifugation technique, and when paired with a batch process, the integrity of the isolated EXOs increased. The reliability of the ATPS approach was proven using Western blot and RT-PCR. This simple and quick separation procedure may aid scientists in isolating and analyzing EXOs (Kim et al., 2015).

As a result of these attempts, newly created, user-friendly, polymer-based kits like ExoQuickTM and Total Exosome IsolationTM kits are now commercially available (Yamada et al., 2012). These kits are now extensively utilized since they do not involve costly gadgets. They need long overnight incubations, though, and operators complain of non-EXOs contaminants, which cause notable variations in outcomes (Taylor and Shah, 2015). In general, Van Deun et al. found that EXOs separated with commercially available kits had lower purity than those obtained by centrifugation techniques (Van Deun et al., 2014). Given the drawbacks of current conventional techniques, a quick, affordable, easy-to-use exosome separation approach with high purity has not yet been created.

EXOs are often characterized by utilizing antibodies that bind to particular receptors, such as MHC antigens (Liu et al., 2025). Naturally, similar antibodies may be employed for separating EXOs; antibodies that are covalently linked to the fixed phase are commonly utilized for this function (Xin et al., 2020). Magnetic beads, extremely permeable monolithic silica columns, the surface of plastic dishes, cellulose filters, and membranes are all useful for achieving this goal (Lee et al., 2024; Yan et al., 2023). The broad spectrum of antibodies and fixed phases has resulted in a huge range of EXOs isolation techniques. As an instance, Clayton et al. suggested an immunomagnetic method to separate B-lymphocyte EXOs from cultured cellular supernatants. The researchers employed 4.5 μm paramagnetic beads labeled with antibodies and cultured them in prepared media for 24 h at ambient temperature. They then separated the EXOs clusters with magnetic granules using a magnet. EXOs with an average diameter of 70 nm contributed to 71.6% of all EVs, whereas those with a dimension of 100 nm or bigger made up 29.4%. In terms of time and functionality, the procedure is equivalent to older strategies. When examining an extensive number of biomarkers and cells for exosome separation, typical ultracentrifugation followed by immunoblotting might take a few days to a week. Flow cytometry study of magnetic bead-exosome complexes necessitates 1 day and utilizes 1 × 10^6^ cells (Clayton et al., 2001).

Microfluidics was developed in the latter part of the twentieth century as a result of breakthroughs in the field of semiconductors. The emergence of microfluidic technology began in the 1980s, coinciding with rapid advances in microelectronics, materials, and systems (Convery and Gadegaard, 2019).

EXOs may be captured and isolated using a variety of dielectrophoretic (DEP) force-based microfluidic devices. In dielectrophoretic separation, polarized dielectric particles are transported in an erratic electric field. DEP forces can be either repulsive or attractive, determined by the polarization actions, but they both cause electrically polarizable particles to migrate (Kwizera et al., 2021). Particle size, volume, used field intensity and frequency, dielectric characteristics, medium pH, and texture all affect this transport process. Systems that adopt these approaches have proven to be more cost-effective, portable, scalable, and process-time-efficient than traditional exosome separation techniques. It has also been claimed that this method makes it possible to analyze EXOs in tiny samples without the need for specialized reagents or costly equipment (Bhadra and Sachan, 2024).

Cho et al. isolated EXOs from the blood plasma by a high-yield electrophoretic migration technique. Compared to the ultracentrifugation approach, this gadget produced eight times as many EXOs. In contrast to traditional methods, the electrophoretic technique may remove up to 83.6% of proteins while recapturing 65% of EXOs. This was accomplished in around 30 min, which is nine times quicker than the traditional ultracentrifugation method (Cho et al., 2016). The most widely utilized isolation technique is the selective capturing of EXOs by antibodies anchored on solid surfaces, albeit each microfluidic substrate has distinct properties and performs differently.

Chen et al. presented a groundbreaking immunological affinity technique for capturing EXOs within a microfluidic chip. The separation concept is based on ligands on the exosome’s external surface, which permit particular gathering based on source and functionality while isolating them from other dispersed membrane components. The gadget has a flat design with herringbone carvings to improve mixing. Following many rinsing processes, captured EXOs are either digested for DNA extraction or characterized *in situ*. Chen et al. showed a speedier approach (∼1 h) with fewer volumes of chemicals (100–400 μL), compared to established methods. EXOs collected on-chip utilizing CD63 antibodies from 400 μL blood samples yielded roughly 30 ng of total RNA for non-small cellular lung carcinoma patients, which demonstrated sufficient integrity (Chen et al., 2010).

Kanwar et al. applied the same idea, adapting the previous approach to perform “on-chip” exosome measurement using a fluorescence assay technique on a typical read-out plate analyzer. The gadget, known as Exochip, consists of numerous circular wells linked by small tubes to improve mixing. Furthermore, the longer duration of retention promotes a greater contact between EXOs and the customized surface. Aside from being specifically designed for additional examination, the gadget can be readily expanded by simply introducing additional rows of wells to the same chip. The ExoChip EXOs produced 15–18 μg of entire protein and 10–15 ng of overall nucleic acid from 400 μL blood specimens. EXOs from pancreatic cancer patients fluoresced more on the chip than those from healthy individuals. This was consistent with the increased protein levels of CD63 and Rab5 detected in cancer patients’ EXOs (Western blot). A collection of miRNAs found in isolated EXOs was also effective in discriminating between carcinoma patients and healthy controls (Kanwar et al., 2014).

Davies et al. pioneered a novel isolation way, by sieving EXOs directly from whole blood via a membrane and controlling filtration using pressure or electrophoresis. The scientists believe that the device’s non-selectivity with regard to vesicle species is a benefit over the ultra-specific capture afforded by immune-affinity-associated approaches that may give rise to prejudicial data processing. A key disadvantage is the poor exosome restoration, notwithstanding the gadget seems to operate effectively with regard to of isolation duration. The apparatus attained saturation after extracting 3–4 μL of filtrate using pressure guided filtering. Electrical based filtering yielded 79 ng RNA per 100 μg protein from a 100 μL specimen, whereas centrifugation yielded 187 ng per 100 μg protein from a 5 mL sample (Davies et al., 2012). This contributes to a quicker separation duration, while the electrical current provides a greater purity of the isolated vesicles (Zhang et al., 2020d).

Urine is a promising source of EXOs called urinary EXOs, which may be acquired non-invasively (Atia et al., 2025). Yet, the resulting amount of EXOs from urine specimens may be inadequate for some investigations because of EXOs immobilization by the THP meshwork. In this context, Puhka et al. designed a simple dilution approach to improve the urine EXOs output by breaking the bond between THP filaments and EXOs using alkaline pH and reduced ionic intensity. The average EXOs production from the dilution process was 2–7 times that of the undiluted control, increasing by 130%–624%. The productivity rose the greatest in samples with an elevated THP to EV ratio. The treatment made no changes to the EXOs’ shape or size spectrum. The KeepEX dilution approach offers a straightforward and effective way to avoid EXOs loss, hence improving urine production. Because KeepEX needs no particular modification of specimen pH or additional centrifugation processes, it might be employed on its own or in conjunction with existing EXOs purification techniques to increase EXOs separation, especially with tiny urine quantities (Puhka et al., 2017).

Yet, the reliability of EXOs’ separation from urine specimens is greatly impacted by the varied composition of urine caused by variables such as hydration, nutrition, and illness. As a biofluid, urine naturally varies in volume, pH, osmolality, and solute concentration over time as well as between people (Zhou et al., 2021). Standardizing pre-analytical urine handling practices is essential since these variations may influence the content and purity of isolated EXOs. This entails standardizing the thawing and subsequent processing procedures, employing protease inhibitors, freezing urine at suitable temperatures (such as −80 °C), and accounting for the time spent voiding (Staub et al., 2025). Hydration levels directly impact urine volume and concentration. While extremely concentrated urine may result in greater quantities of certain compounds that obstruct EXOs’ isolation, very diluted urine can include lesser amounts of EXOs or render their separation more difficult (Tong et al., 2023).

Balaj et al. present an innovative strategy for EXOs separation centered around heparin’s capacity for binding EXOs. EXOs were recovered from conditioned cell media utilizing an agarose sorbent with heparin, Affi-Gel® Heparin Gel (Bio-Rad), which was contrasted to the effectiveness of ultracentrifugation and the ExoQuick-ТC commercial kit. After at least 12 h of incubation at 4 °C, the resin was rinsed with normal saline solution to remove any loose agarose beads. The EXOs extracted from heparinized agarose were architecturally comparable to those produced after normal ultracentrifugation. However, this procedure is fairly extensive, and the biological fluids include a variety of heparin-binding proteins. To enhance EXOs’ productivity while utilizing heparinized sorbents, concentrate the EV portions after separation by ultrafiltration via a 100-kDa filter. However, this prolongs and complicates the separation process (Balaj et al., 2015).

In contrast to animal EXOs, plant EXOs have a wider size range (50–500 nm) (Kumar et al., 2023b), which makes it challenging to extract a homogeneous population utilizing size-based separation methods like size exclusion chromatography or differential centrifugation (Tian et al., 2023). Because a consistent dimension is essential for constant drug loading and targeted delivery, this size variance affects downstream usage, particularly when employing EXOs as pharmaceutical delivery carriers (Han et al., 2022).

However, when it comes to plant EXOs, the collected specimens are first cleaned and then physically treated by mixing, crushing, and squeezing them in buffer solutions. Since plant EXOs are likely to have certain metabolites in common with their parent plants, such as cell walls, chloroplasts, and other membrane vesicles (Yang et al., 2023). Furthermore, exosome formulations may get contaminated by the complex variety of biological substances found in plant cells, such as cell walls, chloroplasts, and other membrane vesicles. These impurities may impair the quality of separated EXOs and impede further studies (Wang et al., 2020).

As a result, each species of plant should be thoroughly screened. For example, blending is a better approach for extracting more bioactive ingredients from grapefruits than juicing or crushing (Li et al., 2022b).

There is no set procedure or set of rules for the development of plant EXOs. Even when the same techniques were used, there were significant differences in procedures as well as the different approach choices across the research we gathered. Specifications like time, velocity, and buffer were among these variations (Garaeva et al., 2021). For example, Regente et al. found that the 40,000×g pellet had a greater density of sunflower seed apoplastic vesicles than the 100,000×g fraction (Regente et al., 2009).

Zeng et al. suggested that 10–20 min of centrifugation at 100,000×g was suitable for extracting Aloe vera EXOs. On the other hand, centrifugation at the same velocity for 60 min produced a very different population with a polydispersity value of 0.59 and a swelling size exceeding 500 nm (Zeng et al., 2021). Actually, the centrifugation process may be challenging since it may be ineffective to pellet the targeted vesicles at low speeds, and prolonged ultracentrifugation may produce impurities that are not vesicles and distort the size profiles (Rutter and Innes, 2020). Additionally, when creating a strategy for the creation of plant EXOs, pH should be taken into account. According to a study, separating ginger-derived EXOs by the PEG-precipitation technique in low pH settings (pH 4 and 5) produced a 4- to 5-fold increase in vesicle production and polyphenolic load in comparison with neutral and alkaline pH environments (Suresh et al., 2021).

For optimal outcomes, the separation and purification processes should be carefully tailored to a variety of criteria, including research objectives, controlled targeting, and laboratory circumstances.

## 4 Characteristics of exosomes

**FIGURE 1:**
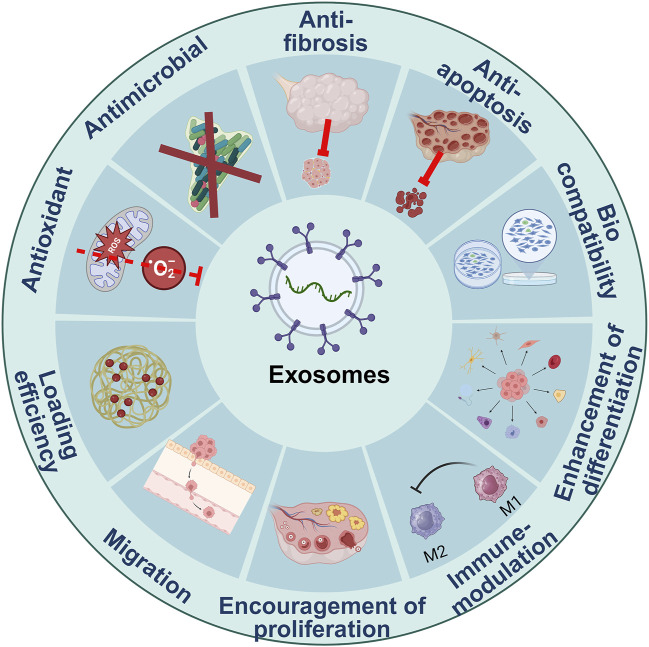
Characteristics of exosomes.

EXOs were first recognized as undesirable cell-based waste; nevertheless, subsequent studies have revealed that EXOs serve as essential biological mediators in interactions between cells, given their capacity to carry biological molecules across the body (Figure 1; Liu et al., 2020d).

### 4.1 Cytocompatibility

Biological compatibility is a critical consideration in the medical use of EXOs. They have shown good biosafety and cytocompatibility when tested (Fordjour et al., 2022). Their cytotoxic properties *in vitro* are assessed by determining the longevity of cells exposed to various exosome doses (Marchante et al., 2023).

According to the kind of host cell, the EXOs protein composition changes and indicates their origin. All EXOs from different cell types share a few groups of proteins (Vaiciuleviciute et al., 2025; Chen et al., 2025).

Numerous studies have shown that EXOs contain mRNAs, miRNAs, and other noncoding RNAs (El Fekih et al., 2025; Cheng et al., 2025). When EXOs circulate, they can be ingested, which ultimately changes the biological functionality of the cells that receive them. Pathological or physiological circumstances may modify the synthesis of EXOs miRNA (Zhu et al., 2025; Ju et al., 2025).

Some lipids that are carried by EXOs are crucial for preserving biological activity. Cholesterol, saturated fatty acid chains, phosphoglycerides, ceramides, and sphingolipids are all transported by EXOs (Palmulli et al., 2024). Crucially, EXOs become stiffer and stable in terms of lipid, which aids in the internalization process (Donoso-Quezada et al., 2021a). However, the host cell is not represented by the lipid composition of EXOs (Elmallah et al., 2022).

Additionally, preclinical testing is required prior to clinical deployment as a vehicle or therapeutic component of the pharmaceutical delivery system (Hajipour et al., 2021).

First of all, the pharmacokinetics, action pathway, target, and mechanism of action of EXOs *in vivo* remain unclear despite their complex biological properties and roles (He et al., 2023). The scientists also noted that a number of critical issues, including pharmacokinetics and targeting, safety assessment, quantification and characterization, and manufacturing techniques, must be resolved before EXOs may be successfully converted into clinical application (Qin et al., 2025).

### 4.2 Immune-modulating effects

The immune system’s function is responsible for producing immunological responses that protect the human body from an attack by harmful organisms. Along with the immune-related organs, the immune system consists of many different immune cells and immunological molecules (He et al., 2021). The immune-modulating capabilities of EXOs such as regulating immune cell behavior and modulating reactions to inflammation, are critical for encouraging tissue regeneration while minimizing unfavorable immunological reactions (Bai et al., 2021).

Additionally, EXOs have the potential to influence immune cell polarization, promoting an anti-inflammatory phenotype. This capability to generate a beneficial microenvironment is essential for tissue regeneration because it reduces excessive inflammation, which can delay the recovery process (Björk et al., 2024). Initial investigations have found that syncytiotrophoblast EXOs suppress the levels of activating markers, generation of cytokines, and lymphocyte and endothelial cell proliferation (Göhner et al., 2017). EXOs generated by B cells and DCs include functional peptide-bound MHC II, in addition to co-stimulatory components CD80 and CD86, which enhance T cell multiplication (Kowal et al., 2019). Administration of endometrial stem cell EXOs (EnSCs-EXOs) could polarize macrophages into an M2-like phenotype and reduce their mediated phagocytosis (Sun et al., 2019).

Xin et al. created a collagen scaffold and exosome construct (CS/Exos) for endometrial regeneration and studied its potential in the management of endometriosis *in vivo*. Endometrial regeneration, collagen remodeling, enhanced expression of the α/progesterone receptor, and fertility restoration were all powerfully stimulated by the CS/Exos transplantation via promotion of CD163+ M2 macrophage polarization (Xin et al., 2020). EXOs play key roles in the pathogenesis of infertility and have a major impact on reproductive health. By transporting different substances, such as proteins, lipids, and RNA that affect follicle growth, oocyte maturation, fertilization, and embryo implantation, EXOs act as messengers for interactions between cells. Through the disruption of these vital reproductive processes, dysregulation or aberrant exosome activity can lead to infertility (Liu et al., 2024b).

Neoplastic EXOs have been demonstrated to contain an increased miRNA content, impede T-cell differentiation and division, and induce apoptosis (Bernardi and Farina, 2021) through the FasL and MARK1 pathways, all of which help the tumor dodge the immune system (Abusamra et al., 2005; Ye et al., 2014).

EXOs from plants can penetrate and control cellular processes in mammals. According to a recent study, intestinal macrophages may absorb plant EXOs and use them to control immunological response (Mu et al., 2014). Macrophages can absorb ginger EXOs, which increase the production of heme oxygenase-1 (HO-1), IL-6, and IL-10. Ginger EXOs produced from carrots cause macrophages to express IL-10. Ginger EXOs produced from grapefruit, carrot, and ginger stimulate macrophages to produce nuclear factor (erythroid-derived 2)-like-2 (Nrf2) (Mu et al., 2014). Ginger EXOs prevent macrophages’ NLRP3 inflammasome from assembling (Chen et al., 2019).

In a different study, Ou et al. investigated *C. roseus* leaves and their apoplastic fluid, a new plant-based chemotherapeutic immune modifier. *In vitro*, 60–240 μg/mL of *C. roseus* EXOs stimulated lymphocyte division and macrophage polarization and phagocytosis. In immunocompromised mice given cyclophosphamide, administration of 20 mg/kg and 60 mg/kg of *C. roseus* EXOs prevented bone marrow cell cycle disruption and white blood cell decrease. *In vitro* and *in vivo*, *C. roseus* EXOs significantly boosted TNF-α production, triggered the NF-κB signal pathway, and elevated the expression of the transcription factor PU.1, which is linked to hematopoietic function. Plant cell cultivation methods of *C. roseus* were developed to produce *C. roseus* EXOs with comparable physical characteristics and biological activity in order to guarantee a consistent supply of these organisms. The growth medium was effectively converted into gram-level EXOs, and the yield was three times more than the initial amount (Ou et al., 2023).

In conclusion, EXOs have a significant impact on female fertility and are essential components of the reproductive system. They are the focus of research with the goal of comprehending and treating infertility because of their function in intercellular communication, controlling autoimmune disease, and enhancing wound healing.

### 4.3 Antioxidative properties

Oxidative stress arises when the equilibrium of free radicals and antioxidant defenses in cells is disrupted. It is linked to several ailments, including diabetes and neurological disorders. Oxidative stress causes cell death via apoptosis (Shaoyong et al., 2022).

Human follicular fluid contains EXOs, which vary in concentration and molecular makeup according to the size of the follicle, the hormonal milieu, and the pathological condition (Pan et al., 2024b). The EXOs miRNA profile in follicular fluid has been shown in several studies to be correlated with the development of embryos, the quality of oocytes, and the success of fertilization. For instance, developed oocytes and high-quality embryos have been favorably connected with miR-21, which is known for its anti-apoptotic and pro-survival functions (Martinez et al., 2018). Bioactive lipids, including sphingomyelin, phosphatidylserine, and ceramide, are transported by EXOs and improve membrane integrity, promote vesicle formation, and affect follicular cell survival and oocyte integrity (Yáñez-Mó et al., 2015).

In addition, EXOs miRNA-21 has been demonstrated to control zygote advancement and growth while suppressing embryonic mortality (Lv et al., 2018). By controlling the MSX1 activity, miR-21 prevents granulosa cell death and enhances hormone production, offering a promising option for the management of autoimmune premature ovarian insufficiency (POI) (Yang et al., 2024b).

According to Xiong et al., hPMSCs-Exo can reduce the senescence of CD4^+^ T cells by delivering miRNA-21 and triggering exogenous antioxidant responses coordinated by the PTEN/PI3K-Nrf2 axis (Xiong et al., 2021). EXOs made from human umbilical cord mesenchymal stem cells (HUCMSCs-EXOs) can maintain homeostasis via modulation of two important effector molecules, manganese-containing superoxide dismutase (MnSOD) and glutathione peroxidase 1 (GPX1) (Yao et al., 2019a; Yan et al., 2017). Interestingly, hUC-MSC-EXOs had greater MnSOD levels than BMMSCs-EXOs (Yao et al., 2019a).

According to de Godoy et al., BMMSCs-EXOs transmit catalase (CAT), which fully restores the baseline neuronal ROS level that was raised by the generation of AβOs (de Godoy et al., 2018). After being treated with H2O2, BMMSCs-exos decrease internal mitochondrial ROS generation, hence exhibiting a mitochondrial-protective function in nucleus pulposus (NP) cells. The mitochondrial proteins that are transported from the EXOs to the NP cells determine the effectiveness (Xia et al., 2019).

However, several miRNAs found in EXOs have been linked to controlling steroidogenesis, atresia, and follicular development. MSCs-EXOs can enhance the production of anti-Müllerian hormone (AMH) and facilitate the shift from primordial to primary follicles. EXOs have the potential to assist in reestablishing homeostasis in the injured ovarian milieu by providing a mix of advantageous chemicals (Yu et al., 2016; Dalmizrak and Dalmizrak, 2022).

Following myocardial infarction and hypoxia, MSCs-EXOs can alleviate cardiac dysfunction. Myocardial ischemia-reperfusion (I/R) damage can be treated by miR-182-5p, which is transported by MSCs-EXOs, according to studies. Comparable to miR-182-5p in rat myocardial cells’ reaction to I/R, miR-199a-3p and miR-214 may both increase myocardial cell viability and hence cure myocardial ischemia-reperfusion damage (Yue et al., 2022).

MSCs-EXOs can mitigate the cytotoxicity of LPS-induced astrocytes by blocking the expression of inflammatory astrocyte proliferation biomarkers like GFAP, C3, and CD81, while increasing Ki67. Furthermore, it can lower the production of cytokines associated with inflammation, including TNF-α and IL-1β (Xian et al., 2019).

Xiang et al. create milk-derived EXOs as a unique, effective, and non-toxic siRNA carrier in order to investigate therapeutic delivery techniques. After siRNA-Keap1 (siKeap1) was sonicated into milk EXOs, it was shown that the resulting mEXOs-siKeap1 relieved oxidative stress in MGO-treated HUVECs and promoted HUVECs movement and multiplication. In contrast, mILK EXOs-siKeap1 injection drastically sped up diabetic wound healing in a mouse model of diabetic wounds by promoting collagen production and neovascularization. When combined, these findings show that milk EXOs may be used as an adaptable, biocompatible, and economical siRNA delivery technology and promote the advancement of Keap1 knockdown as a possible therapeutic approach for diabetic wounds (Xiang et al., 2023). As possible cargo molecules involved in intracellular communication and post-translational gene activity, EXOs microRNAs (ExomiRs) are essential in diagnostics. For example, exomir-122-5p can be employed as a prognostic biomarker for detecting gestational diabetes mellitus (GDM), since it inhibits the proper function of genes such as Glucose-6-Phosphate Catalytic Subunit 3 (G6PC3), which is necessary for the hydrolysis of glucose 6-phosphate in glycolysis, resulting in insulin resistance and, ultimately, GDM in patients (Ye et al., 2022).

### 4.4 Encouraging the inter-cellular communication

#### 4.4.1 Embryonic/implantation role

By carrying regulatory molecules like miRNA from donor to recipient cells, EXOs help cells communicate with one another. For instance, miR-21-5p and miR-30d encourage placentation (Zhang et al., 2024). The blastocyst communicates with and controls the endometrium during embryo implantation, and the embryo is nurtured by endometrial fluid generated by the endometrial epithelium (Bai et al., 2023; Vilella et al., 2015).

EXOs miRNAs and EXOs proteins both play key roles in embryo implantation. Research found that Hsa-miR-30d, released by EXOs secreted by human endometrial cells, is absorbed by the mouse embryo (Vilella et al., 2015).

By specifically targeting histone deacetylase 9, miRNA-30d-5p from placenta-derived EXOs mechanistically caused macrophage polarization to the M2 phenotype. Additionally, they stimulated trophoblast invasion and migration. In contrast, the conditioned media hindered the transfer and development of endothelial cell tubes. T-cell proliferation was unaffected by macrophages treated with placenta-derived EXOs. In conclusion, EXOs produced from the placenta polarize macrophages to take on the characteristics of decidua-like macrophages, which in turn alter the activities of trophoblasts and endothelial cells (Bai et al., 2023).

*In vitro,* amniotic epithelial cells (AEC) EXOs activated NF-κB and COX-2, contracting proteins, causing uterine myometrial cells to contract. The same mouse study demonstrated that dye-labeled EXOs administered intra-amniotically into pregnant mice traveled into the mother mice’s bloodstream and kidneys. EXOs have been shown to pass the placenta and disseminate throughout the bloodstream (Sheller-Miller et al., 2016).

#### 4.4.2 Aging related roles

The age-related decline is connected with the development of the SASP, which may aid in phagocytosis-mediated clearance of aging cells. EXOs contain a high concentration of the Wnt signals, which are crucial for the preservation of homeostatic balance and are implicated in the aging process (Zhang et al., 2020b). As a consequence, it has been suggested that EXOs are SASP messengers. They are secreted by aged fibroblasts and epithelial cells. The aged also have an abnormal EXOs composition; for example, galectin-3, which is required for bone cell development, is drastically diminished. EXOs recovered from old individuals may arise from a lack of bone stemness (D’Anca et al., 2019).

Numerous physiological processes and illnesses are linked to the Wnt/β-catenin signaling system, which is home to a large number of glycoproteins with distinctive properties. It can take part in tissue reconditioning, physiological homeostasis, and growth and development (Jung and Suh, 2015). According to several studies, MSCs and their EXOs use the Wnt/β-catenin signaling pathway to help cure disorders of the skin, cardiovascular system, neurological system, and other areas. BMMSCs-EXOs at a dose of 100 mg/mL, and the results demonstrated that BMMSCs-EXOs could raise the levels of Bcl-2, β-catenin, and TCF-4 while drastically lowering the degree of protein expression of Bax, cleaved caspase-9, and cleaved caspase-3 (Hromadnikova et al., 2015).

Additionally, by lowering oxidative stress, encouraging DNA repair, restoring BMMSCs’ activity, stimulating the Wnt/β-catenin cascade, and reestablishing the lipogenic-osteogenic equilibrium, BMMSCs-EXOs can help alleviate osteoporosis (Zuo et al., 2019).

### 4.5 Promoting cellular differentiation

The development of cells is an intricate procedure that involves the anatomical and functional modification of cells, leading to the production of diverse cell types (Zakrzewski et al., 2019). This mechanism is predominantly connected with embryonic growth, but it also promotes the renewal and repair of tissues. Repair of damaged organs requires directing specific cell differentiation pathways of cells (Yin et al., 2020).

The EXOs cargo consists of various proteins, lipids, and nucleic acids (DNA, mRNA, and short RNAs. Noncoding, endogenous, single-stranded RNAs with a length of 18–25 bases, microRNAs (miRNAs) mostly inhibit their target genes at the post-transcriptional phase (Wang et al., 2018). There is growing evidence that miRNA-regulated epigenetic modifications are linked to various illnesses, such as osteoporosis and metabolic disorders. By encouraging the proliferation and migration of pig trophoblast cells (PTr2) through its target gene phosphofructokinase-M (PFKM), miR-92b-3p can regulate embryo implantation (Wang et al., 2022c).

Furthermore, recent research in pigs has shown that miR-92b-3p, an EXOs generated from pigs’ endometrium, could control the division, movement, and adherence of trophoblasts (Hua et al., 2022). Additionally, EXOs have been linked to the formation of oocytes. Previous research has shown that bovine follicular EXOs can improve oocyte maturation by enhancing cumulus cell expansion (Hung et al., 2015).

The process by which tiny primordial follicles develop into giant preovulatory follicles, which partly takes place throughout the oestrus cycle, is known as folliculogenesis. Most follicles commit to atresia during folliculogenesis, but a small percentage become Graafian follicles. Peroxisome proliferator-activated receptor gamma (PPARγ), the target of miR-27b, is essential for the maturation of pig oocytes, whereas miR-202 is gonad-specific and may help avoid premature ovarian failure (POF) (Song et al., 2016). In humans, miR-15a may control BCL2 and cell division cycle 25A (CDC25A) to control oocyte development and maturation (Xu et al., 2011), while miR-335-5p regulates developing spindles and cytoskeleton activity in mice oocytes through MAPK signaling (Cui et al., 2013). Through the Notch2/TIM3/mTORC1 axis, EXOs miR-18b improves trophoblast recruitment and division, hence alleviating preeclampsia (Yang et al., 2021b).

### 4.6 Exosomes as pharmaceutical carriers

The emergence of EXOs-tailored delivery methods has created new avenues of optimism for targeted pharmaceutical delivery (Ogunnaike et al., 2021). According to research, the potency and purity of EXOs, in addition to their number, possess a tremendous influence on the success of treatment approaches (Machtinger et al., 2021; Andronico et al., 2019). Creating a consistent and reproducible strategy for obtaining high-quality EXOs is crucial (Liang et al., 2021). EXOs’ unique qualities, such as intrinsic stability, minimal antigenicity, and high infiltration capability, have made them a popular choice for building tailored delivery devices (Taravat et al., 2024). Despite developing EXOs as drug transporters presenting several obstacles, it is moving quickly. EXOs administration technologies have fundamental challenges in entering clinical trials due to swift elimination from the circulatory system and insufficient targeting capabilities (Zhang et al., 2022a). In fact, other engineering procedures have been devised to produce modified EXOs with greater effectiveness. EXOs can be customized in two ways: 1 interior adjustments, which include integrating drugs and bioactive ingredients, and 2 external changes, which customize the exosome’s surface to target specific cells or tissues (Li et al., 2024a).

#### 4.6.1 Cargo packaging into exosomes

Endogenous and exogenous cargo loading techniques are the two primary groups into which exosome cargo packing techniques fall (Gul et al., 2024). Exogenous cargo loading involves directly loading medications into the retrieved MSCs’ EXOs, whereas endogenous cargo loading involves modifying parental cells using viral vectors and plasmids (Kumar et al., 2023a). Viral vectors and plasmids are examples of genetic engineering tools that may be used to modify the expression levels of endogenous molecules in stem cells (Farzanehpour et al., 2023). Exogenous cargo loading strategies for managing illnesses include saponin permeabilization, freeze-thaw cycles, and room temperature incubation (Ahmed et al., 2024).

#### 4.6.2 Surface modification

Notwithstanding their natural origin, EXOs may be easily surface-changed. Genetic engineering and chemical modification are two types of modification techniques. Genetic engineering entails integrating the genetic sequencing of a directing protein or polypeptide with that of a EXOs membrane protein. This approach functions effectively for expressing peptides and proteins on the surface; however, it is limited to targeting arrangements that are genetically programmed (Cheng et al., 2022).

Chemical modification enables a vast variety of ligands to be demonstrated via conjugation methods or lipid assembly. Conjugation processes can covalently and stably change EXOs surface proteins, although the complicated nature of the exosome surface can impair reaction efficiency, and site specificity is frequently lost (Chu et al., 2022; Sengupta et al., 2020). Covalent alteration may potentially threaten the vehicle’s structural and functional integrity and may increase the toxicity of EXOs (Smyth et al., 2014).

Notwithstanding encouraging existing accomplishments, there are only a few investigations that reveal EXOs to be superior to FDA-authorized nanomedicines (e.g., liposomes); therefore, further research into EXOs as carriers of medication is unavoidable (Lu et al., 2018; Zhang et al., 2023b).

EXOs are more bioactive and antigenic than liposomes because they are primarily generated by cells, which improves their stability in the circulation and increases their absorption capacity and medicinal efficacy *in vitro* and *in vivo* (Bethi et al., 2025; Aare et al., 2024).

Liposomes, on the other hand, have three key drawbacks that drastically limit their therapeutic use. First, liposomes may be unable to endure shear pressures or variations in environmental factors or diluent content. Second, liposomes are exceedingly sensitive to environmental stimuli and reactions, making them unsuitable for widespread application in medication administration. Third, it is challenging to precisely transport substances within liposomes to specific locations *in vivo* (Smyth et al., 2015).

## 5 Formulation of exosome agents

**FIGURE 2:**
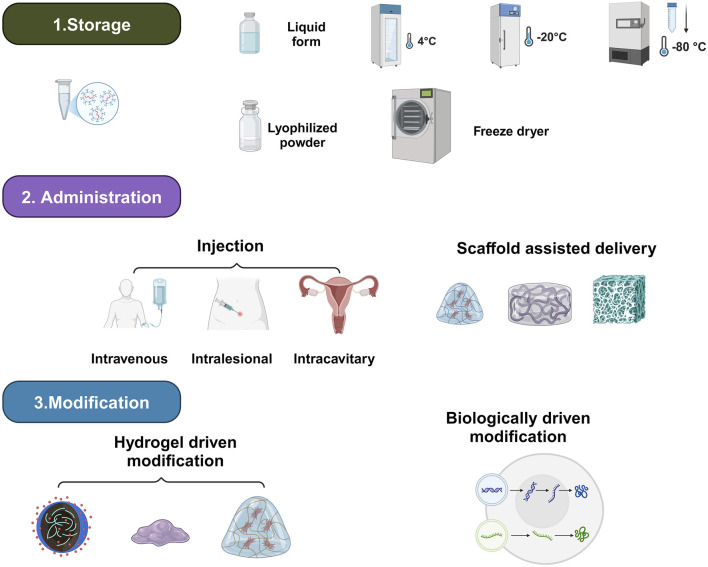
Formulation of exosome agents.

To enhance their therapeutic effects, exosome preparation studies should focus on three critical aspects: storage, delivery modalities, and therapeutic enhancement (Figure 2; Table 1; Donoso-Quezada et al., 2021b).

**TABLE 1:** Pitfalls in EV studies.

EV-related procedures	Pitfalls	Reference
Gathering	The optimum methodology depends on the kind of fluid, and cellular origin of the EVs, along with downstream assessment.	()
Different isolation methods	Consistency in EXOs separation techniques is necessary to avoid variations in EXOs content and size throughout the analysis.	Konoshenko et al. (2018)
Storage	To prevent vesicle breakdown, freeze samples at 80 °C. Additionally, it is recommended to prevent repetitive Freezing-thawing of the portions before testing.	Lee et al. (2016)
Purity	Sample duplicates and adjustment of outcomes for numerous evaluations are critical.	Rupert et al. (2017)

### 5.1 Storage

EXOs are a potential cell-free treatment; nevertheless, they cannot attain activity for very long. As a result, studies on exosome storage technologies are necessary to maintain their biological activity while also making them easier to carry and apply in therapeutic settings (Levy et al., 2023). At present, the most common protective strategy is the storage in freezers, freeze-drying, and spray-drying (Song et al., 2020). Traditionally, EXOs are preserved at 4 °C, −20 °C, and −80 °C (Levy et al., 2023). Despite notable variations in conclusions, several studies have evaluated the influence of temperature on storage (Klymiuk et al., 2024; Rashidi et al., 2022). For example, Levy et al. proposed that EXOs stored at −20 °C and prolonged freeze-thawing resulted in EXOs aggregation. Wu et al. supported this result by seeing a drop in both the overall protein quantity and general RNA concentration at more elevated storage temperatures (RT, 4 °C) and following repeated intervals of freeze-thawing (Wu et al., 2021a). Furthermore, Van De Wakker et al. revealed that bioactivity of BMMSCs-EXOs is reduced after storage at room temperature and 4 °C, but storage at −20 °C, −80 °C, or lyophilization typically retains bioactivity for up to 4 weeks (Van De Wakker et al., 2022). Maroto et al. found that keeping EXOs for more than 4 days, whether at 4 °C or −80 °C, had a negative impact on their proteomic composition (Maroto et al., 2017). Yuana et al. obtained cell-free pee and kept it in freezing conditions for a year before collecting the EXOs and comparing them to those recovered from fresh urine. The quantity of EXOs extracted from a fresh urine sample was 109–1,010/mL, which reduced twofold following a single freeze-thaw cycle. EXOs’ diameter rose by 17% after storage. However, no morphological alterations were seen during storage (Yuana et al., 2015). In general, research suggests that storing EXOs-containing urine samples at −80 °C with protease inhibitors is effective for long-term preservation.

Spray drying begins by atomizing the EXOs solution, then, when exposed to a hot gas, the droplets are swiftly changed into a dry powder. When compared to freeze drying, spray drying is quicker, requires only one stage, and serves as an ongoing drying procedure, making it more cost-effective (Chernyshev et al., 2015). Spray drying is recommended for heat-sensitive ingredients (Singh et al., 2023). Furthermore, water retention can worsen chemical fragility by lowering the glass transition temperature of solid fragments. Further research is needed to bring this technology to the development of EXO-based therapeutics (Emami et al., 2023).

Lyophilization has recently emerged as a viable alternative to standard 80 °C storage. Lyophilization not only increases exosome storage time by allowing direct room temperature preservation, but it also lowers preservation expenses. Lyophilized substances can be kept at room temperature and quickly reproduced in water or a physiological solution (Liu et al., 2021). Although lyophilization has storage advantages, it also has substantial drawbacks like ice crystallization, dehydration, and osmosis, which may jeopardize the dimensional stability and composition of EXOs cargos and membranes. Lyophilized EXOs using lyoprotectants such trehalose and sucrose show superior diversity in sizes, structural reliability, particle amount, and protein/RNA content preservation than those held at −80 °C (Arte et al., 2025).

Investigators conducted trials with the inclusion of several lyoprotectants to prevent lyophilization damage while maintaining exosome integrity and size. Before getting freeze-dried, these compounds may bond with phospholipid motifs, dislodging moisture and producing a glassy lattice of sugar (Chen T.-Y. et al., 2024). This matrix inhibits ice crystal formation, minimizing vesicle damage and aggregation (Abla and Mehanna, 2022). In this procedure, effectiveness may be influenced by the application of various cell origins as well as initial separation techniques (Merivaara et al., 2021).

### 5.2 Administration

EXOs’ research has made significant progress in cell-free medical applications around the world (Moghadasi et al., 2021). Several techniques for targeted exosome delivery have been studied, including direct application, intravenous infusion, intraperitoneal injection, swallowing, and hydrogel-based encapsulation (Moss et al., 2021).

Direct administration can be administered via intravenous injection or topically in tissues, which is a typical method of exosome treatment. EXOs rapidly leave the circulation and aggregate in parenchymal tissues, with a plasma half-life of only 2–4 min (Driedonks et al., 2022). Local delivery can be more beneficial in terms of enhancing the amount and sustainability of their effects (Sanz-Ros et al., 2022). Intranasal administration is more successful, especially in avoiding the difficulties associated with transporting medications across the blood-brain barrier (BBB). The intranasal approach reduces exosome loss by bypassing intestinal and hepatic routes (Guo et al., 2019). According to investigations, intranasal injection of EXOs containing curcumin and cucurbitacin led to fast transport to the mouse brain. This platform can boost tumor apoptosis and reduce metastasis. Curcumin-loaded EXOs demonstrated a substantial decrease in microglial cell count (Zhuang et al., 2011).

Exosome injections without scaffolds have disadvantages, including systemic uptake and lower efficiency at the defect location, prompting the usage of exosome-loaded scaffolds. This exosome-delivery technology has the potential to be highly successful in tissue healing. Meanwhile, certain kinds of mesenchymal stem cells (MSCs)-EXOs aggregate in damaged tissues, causing inflammatory responses and other pathological alterations (Ghafouri-Fard et al., 2021). EXOs from adipose tissue-derived MSCs have been shown to increase the expression of miR-122. Increased levels of miR-122 inhibited LX2 cell growth by targeting the P4HA1 gene. This miRNA has been demonstrated to inhibit collagen maturation and extracellular matrix formation (Li et al., 2013). As a consequence, the invention and utilization of EXOs-loaded scaffolds for effective and controlled release have arisen as a captivating study subject in regenerative medicine (Li et al., 2023a). Non-invasive intracavitary injection is an excellent treatment for uterine and vaginal damage (Lv et al., 2020).

The development of ways to extend the half-life and local longevity of EXOs is a critical challenge for their therapeutic use. According to the research, mixing EXOs with biomaterials may be the most appropriate answer to this difficulty (Zhu Y.-G. et al., 2022). A desirable biomaterial should be capable of maintaining exosome biological stability while also regulating release kinetics in accordance with a favorable release schedule (Akhlaghpasand et al., 2024). Lin et al. discovered that AMSCs-|EXOs loaded into injectable PEG hydrogels provide antimicrobial capacity for the endometrial environment, promote endometrium regeneration, and fertility reconstruction (Lin et al., 2021b).

### 5.3 Therapeutic enhancement

When establishing EXOs as biological therapies, simply addressing storage issues is insufficient for EXOs to engage in important therapeutic transformations. The limited extraction amount and longevity of EXOs have led investigators to focus on exosome modification (Zhou C. et al., 2023). Repeated injections are not viable in the clinic, necessitating the development of improved delivery methods with high tissue intake, biosafety, and simplicity of application (Wang et al., 2022b). While topical exosome administration seems promising, there are some drawbacks, such as limited skin penetration, variability in exosome creation and characterization, and a lack of established techniques. EXOs may have a limited half-life *in vivo*, necessitating several doses or sustained-release preparations to obtain the desired therapeutic effects While efforts have been made to address these concerns, there is still a need for simple and efficient solutions (Wan et al., 2023). An increasing amount of research suggests that providing medium conditioned with mesenchymal stem cells might be a viable option for live cell treatment. MSCs have an excellent safety profile and may be preserved without losing their regenerative potential (Lin et al., 2021b). It is adaptable enough to be used in a variety of delivery vehicles, improving engraftment and controlling therapeutic administration.

Hydrogels are a potential way to regulate exosome delivery, but they have significant disadvantages (Zhang et al., 2023a). These include obstacles in ensuring continuous release, possible concerns with the hydrogel’s mechanical strength and stability, and challenges associated with large-scale manufacture (Zhang et al., 2023a). Furthermore, chemical and physical interactions between EXOs and the hydrogel matrix can influence exosome release and therapeutic effectiveness (Ghahremani-Nasab et al., 2025).

Liu et al. discovered that hydrogel cross-linking may lengthen the releasing duration of EXOs in rats from 4 to 7 days, resulting in a stronger therapeutic efficacy at the same dosage. The hydrogel’s 3-D matrix allows a wide range of medicines to cross-link, increasing the therapeutic value (Liu et al., 2019).

## 6 Medicinal advantages of EXOs in female infertility conditions

EXOs have been shown to be a promising therapeutic device for carrying payloads in the treatment of female infertility (Teng et al., 2024; Tuscharoenporn et al., 2025; Bhat et al., 2022). However, the pathophysiological processes of EXOs in female infertility have not been fully understood. More studies must be conducted to determine the cause and give proof for potential therapeutic treatments (Liu et al., 2024a).

### 6.1 Treatment of endometriosis

Endometriosis is a multifaceted illness associated with inflammatory processes, blood vessel development, and apoptosis tolerance. Eutopic endometrium (EUE) in endometriosis patients contributes to the disease’s development and promotes ectopic endometrium (EE) survival by regulating many molecular pathways (Zhu et al., 2023). Endometriosis can manifest in a variety of ways, ranging from asymptomatic lesions discovered by chance to a severe condition that is unrelated to the extent of the disease. Most typically, the initial symptoms appear before the age of 20 (Wang et al., 2023).

Manifestations of endometriosis include persistent pelvic discomfort, extremely excruciating periods, painful intercourse, urination, and/or painful bowel movements. It could also raise the chance of behavioral wellness concerns, like anxiety and sadness. Endometriosis can also cause infertility without accompanying other manifestations (Lin et al., 2021b). Endometriosis can affect fertility through a variety of mechanisms, including deformed pelvic cavity morphology, adherence development, fallopian tube fibrosis, localized inflammation of pelvic systems, immunological dysfunction, alterations in the hormonal homeostasis within the uterus, and/or deficient embryo implantation (Neto et al., 2024; Dabi et al., 2024).

**FIGURE 3:**
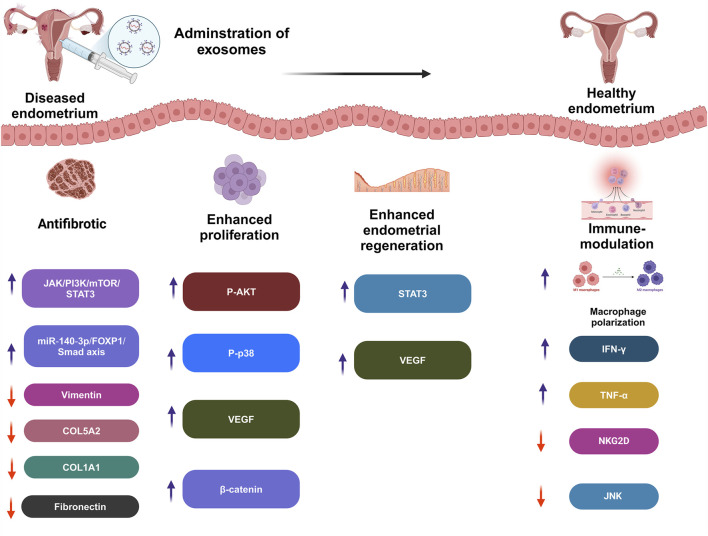
Effect of exosomes therapy on diseased endometrium.

Furthermore, the condition has a substantial negative influence on the standard of living and emotional health because of discomfort and other complaints such as exhaustion, excessive bleeding, or erratic emotions. Women may be unable to attend school or work, and may avoid sexual activity (Qin et al., 2024). One of the primary processes involved in disorders characterized by cell division and penetration is inflammation, which is produced by immunological dysregulation. Endometrial lesions are formed and further developed by immunological cells. In the case of endometriosis, proinflammatory mechanisms inhibit apoptotic processes, causing potentially dangerous cells to cling to distant regions, which demonstrates the benefits of EXOs in endometriosis management (Figure 3; Moghaddam et al., 2022).

Lin et al. discovered that intra-uterine injection of poly-ε-l-lysine hydrogel-loaded with human umbilical cord mesenchymal stem cells EXOs (HUCMSCs-EXOs) and spermidine prenatally increased pregnancy frequency in mice with a weak endometrial lining. This platform demonstrated much higher expression of integrin-β3, LIF, and VEGF proteins. These characteristics enhance and extend endometrial function (Lin et al., 2024). EXOs produced from ectopic embryonic stem cells were shown to induce M2 macrophage transition by releasing miR-146a-5p, via TRAF6 (Ji et al., 2024). Frequent abortion, curettage, or intrauterine infection can cause serious harm to the endometrium, potentially leading to pathological disorders and sabotaging fertility (Fernández et al., 2021). The primary goal of uterine infertility therapies is to promote endometrial regeneration (Zou et al., 2025). Traditional treatments have limited effectiveness, highlighting the need for new therapies to enhance endometrial regeneration (Table 2; Feng et al., 2021).

**TABLE 2:** Exosomes in the management of endometriosis.

Type of exosomes	Preparation method	Size	Model	Outcomes	Reference
ADSC-EXOs	Precipitation	30–200 nm	*In vivo*: Rats	Improved uterine structure, endometrial regeneration, collagen reorganization	Zhao et al. (2020)
peritoneal macrophages (pMφ) exosomes	Differential centrifugation	105 ± 3.9 nm	*In vitro*: Ectopic ESCs)	Enhanced cell division, immigration, and penetration.	Zhang et al. (2020a)
EMS pMφ -EXOs	Gradient centrifugation	100 nm	*In vitro*: macrophages *In vivo*: Mice	Increased the amount and weight of endometriosic implants and increased MII pMφ counts. EXOs were absorbed by pMφ, resulting in MI and MII polarizing were changed, and phagocytic function was reduced.	Sun et al. (2019)
EXOs-HP	Differential centrifugation	72.34 nm	*In vitro*: HEnSCs and glandular cells *In vivo*: Mice	Reduced fibrotic progression markers and restored the endometrium physiologic activities.	Lin et al. (2023)
CTF1-modified BMSCs-EXOs	Differential centrifugation	60–120 nm	*In vitro*: HUVECs *In vivo*: Rats	*In vitro*: better neovascularization, including increased multiplication, movement, and tube genesis. *In vivo*: promote tissue regeneration, neovascularization, and inhibit localized tissue fibrosis.	Zhu et al. (2022a)
Endometriotic EXOs	Sucrose gradient ultracentrifugation	120 nm	*In vitro* cytotoxicity: PBMCs and the erythroid cell line K562.	Endometriotic protection from cytotoxic attacks apoptosis in activated immune cells.	Björk et al. (2024)
EMS –EOXs- miR-301a-3p	Differential centrifugation	80 nm	*In vitro*: Human mononuclear cell line THP-1	Induced M2 transformation in macrophages	Huang et al. (2022b)
EECs-EXOs	Ultrafiltration	80 nm	*In vitro*: Ectopic ECs and normal ECs *In vivo*: Mice	Inhibited infiltration and migration capacity of ectopic nodules.	Zhang et al. (2022a)
UC-MSC EXOs/CS	Differential centrifugation	136 nm	*In vitro*: Peritoneal macrophages from SD rats *In vivo*: Rats	Significantly improved endometrial regeneration, collagen remodeling, lowering inflammation and increased anti-inflammatory mechanisms.	Xin et al. (2020)

ADSC-EXOs, exosomes derived from adipose-derived mesenchymal stem cells; BMSCs, BMSCs; CS, collagen scaffold; CTF1,Cardiotrophin-1; ESCs, Embypnic stem cells; EMS, endometriosis; EMS, exosomes from endometriosis; EXOs, Exosomes; EXOs-HP, mesenchymal stem cell-derived exosomes encapsulated in heparin-poloxamer hydrogel; HEnSCs, human endometrial stromal cells; HUVECs, human umbilical vein endothelial cells; PBMCs, Peripheral Blood Mononuclear Cells; pMφ, peritoneal macrophages; UC-MSC, Umbilical cord-derived mesenchymal stem cell.

Intrauterine adhesion (IUA) induced by endometrial damage is one of the most common causes of infertility in women of reproductive age and needs sophisticated therapeutic options (Huang et al., 2022b). Zhao et al. investigated adipose stem cells (ADSCs)/EXOs and evaluated the possibility of their use in intra-uterine adhesions (IUA) in rats. Following ADSCs-EXOs administration, the uterine cavity grew, the endometrium’s surface recovered epithelialization, and endometrial glands increased, along with fewer fibrotic regions (Zhao et al., 2020). In this regard, Jin et al. created an extracellular matrix (ECM)/ADSCs-EXOs scaffold that was cytocompatible and could enhance cellular division, motility, and revascularization *in vitro*. In addition, when implanted in rats, they enhanced endometrium regeneration, increased local angiogenesis, encouraged myometrium rejuvenation, and, ultimately, retained fertility (Jin et al., 2023).

Moreover, Lin et al. developed thermally sensitive poloxamer hydrogel loaded with EXOs to enhance EXOs’ bioavailability in the uterus. In the IUA model, this platform significantly repaired the activity and morphology of the endometrium by inhibiting fibrotic advancement markers (Lin et al., 2023). EXOs released by peritoneal macrophages (pMφ) can effectively transfer to endometrial stromal cells (EnSCs). EXOs from EMS containing pMφ increased EnSCs proliferation, migration, and invasion rates. MiR-22-3p levels were considerably elevated in pMφ-derived EXOs from EMS, which were then transferred to EnSCs via EXOs. EXOs miR-22-3p from pMφ increased EnSCs division, movement, and penetration by engaging SIRT1 and stimulating the NF-κB pathway (Zhang et al., 2020a).

EXOs from endometrial epithelial cells enhance embryo advancement, growth, and placement, whereas the SS performs a selective function in mouse embryo development (Gurung et al., 2020). HUVECs treated with canine bone marrow stem cells (C-BMMSCs)-EXOs showed better cellular division, migration, and tube formation, indicating increased neovascularization (Zhu Q. et al., 2022). EMS-originated EXOs miR-301a-3p regulate the polarization of macrophages via the PTEN-PI3K system (Huang et al., 2022b).

Abnormal accumulation of extracellular matrix in endometrial glands causes endometrial fibrosis, which impairs uterine function. Thus, it is critical to investigate endometriosis fibrosis therapy. Two distinct study groups found that EXOs miR-214 or miR-214-3p produced from ectopic endometriosis stromal cells prevented fibrosis by targeting cellular communication network-2 (CCN2), which is strongly associated with fibrogenesis (Wu et al., 2018; Zhang et al., 2021c). Furthermore, Zhang and colleagues demonstrated that EXOs played a critical role in the delivery of miR-214-3p for fibrosis therapy (Zhang et al., 2021c).

MiR-30c-loaded EXOs from ectopic endometrial cells (EECs) reduced the metastatic development of ectopic EEC nodules. EEC-derived EXOs supplied miR-30c, which blocked BCL9 transcription and suppressed the Wnt/β-catenin system, reducing tumor-like characteristics of ectopic ECs in EMS (Zhang et al., 2022a). Previous research has shown that UCMSCs-EXOs, as regenerative nano-conveyors, perform a comparable function to their parent cells in easing fibrosis, boosting division, and immune-modulation (Pu et al., 2023).

Xin et al. blended UCMSCs EXOs and collagen scaffold (CS/EXOs) construct for endometrium rejuvenation in rats. The CS/UC-MSC-EXOs transplantation considerably encouraged endometrial regeneration, collagen reconstruction, hormonal activity, and fertility restoration. Moreover, it promoted CD163+M2 macrophage polarization and decreased inflammatory reactions (Xin et al., 2020). UCMSCs-EXOs combine the benefits of hUCMSCs’ pluripotency with nanoscale dimensions, improving their therapeutic potential through longer circulation half-life. Notwithstanding these intriguing traits, investigations concerning their immunological toxicity are yet limited (Dehghani L. et al., 2024; Mao et al., 2024).

### 6.2 Management of polycystic ovary syndrome (PCOS)

PCOS is a neglected, underdiagnosed, and understudied illness that impacts a significant percentage of the female population worldwide, particularly in developing countries (Alesi et al., 2022). Women with PCOS remain undiagnosed in early care. As a result, it puts an economic burden on healthcare providers. It is also marked by ovulation problems, which can result in fertility issues (Siddiqui et al., 2023). The pathophysiology of PCOS is complex and influenced by the combination of reproductive and metabolic diseases (Koike et al., 2023). PCOS is characterized by hyperandrogenism and insulin resistance, which are further exacerbated by hypothalamic-pituitary-ovarian axis dysfunction (Liao et al., 2021).

It has been suggested that the oocyte and its adjacent cumulus cells (CCs) exhibit a mutually advantageous connection in the initial phase of developing follicles, of which CCs are primarily accountable for releasing growth hormones and ovarian steroid hormones, demonstrating that CCs perform essential functions in oocyte development (Sun et al., 2023). However, atresia is brought on by oocyte dysfunction brought on by aberrant CC cell division or apoptosis, which is in line with research showing that CCs’ abnormal cell functions are linked to infertility, anovulation, and collapse in follicle maturation—all of which are manifestations that PCOS patients also experience (Yang et al., 2021a).

**FIGURE 4:**
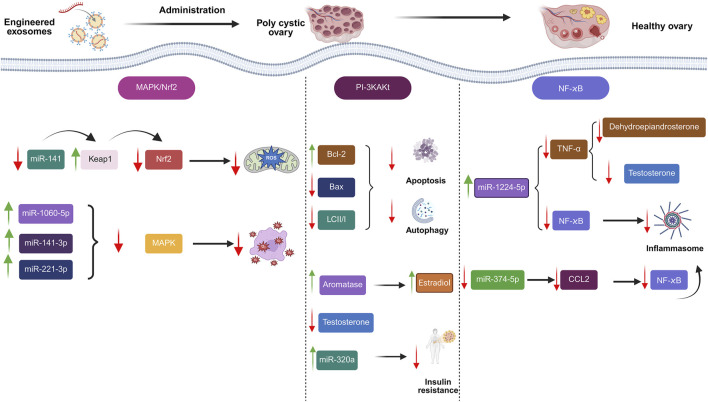
Engineered exosomes in management of polycystic ovarian syndrome (PCOS).

Exosome-based medicines were investigated as a viable therapeutic technique for treating PCOS (Figure 4; Hadidi et al., 2023; Fang et al., 2024; Jiang et al., 2023; Mansoori et al., 2024). Cao et al. proved that amniotic mesenchymal stem cells (AMSCs)/EXOs can provide protection against metabolic abnormalities, alleviate dehydroepiandrosterone (DHEA)-induced PCOS in rats, while increasing their fertility. After 3 weeks, injecting AMSCs-EXOs into PCOS rats can improve hepatic malfunction, ovarian cysts, and infertility caused by DHEA. Moreover, there was a noticeable decline in T levels. Adiponectin secretion was also enhanced by AMSCs-EXOs therapy (Cao et al., 2022). Exosome treatment increased cell division and inhibited apoptosis in CCs via upregulating miR-323-3p (Mehravar et al., 2025). Based on the outcomes of an investigation conducted by Zhou et al., EXOs derived from ovarian follicular fluid reduced PTEN transcription and lowered apoptosis. In rats with PCOS, these EXOs increase estradiol (E2) levels while decreasing LH and FSH concentrations, indicating that they may help follicular fluid (FF) ameliorate the condition (Zhou et al., 2022). HUCMSCs/EXOs could increase anti-inflammatory mediator IL-10 while suppressing inflammation-related mediators. Moreover, they could suppress apoptosis while increasing progesterone synthesis. Antral follicle count (AFC), testosterone (T), body mass index (BMI), and baseline levels of LH were all considerably greater in the PCOS group than in the healthy control group (P < 0.01). Nonetheless, the PCOS group’s baseline FSH level was much lower than that of the healthy control group (P = 0.033) (Zhao et al., 2022).

EXOs have shown promise in the treatment of PCOS, although there may be concerns to take into account. These include the possibility that EXOs might worsen pre-existing problems by carrying hazardous cargo, as well as dangers related to the EXOs’ source (such as human biologics or disease transmission). In particular, miRNAs that stimulate granulosa cell death or cancer cell migration may be present in EXOs from PCOS patients (Bai et al., 2022). In addition, further study is required to completely understand the long-term benefits and potential negative outcomes of EXOs, as their usage in PCOS therapy is still relatively new.

### 6.3 Exosomes in primary ovarian insufficiency (POI)

Sex hormones are generally known they regulate the development of eggs and the functioning of reproduction. They have been shown to have pleiotropic effects in both men and women. Furthermore, alongside their transcription in typically targeted tissues, such as the ovaries and the uterus, their receptors have been discovered in other tissues, such as the bone and the circulatory system (Yan et al., 2022a). POI is an impairment of normal ovarian functionality taking place before reaching the age of forty. Menstrual irregularities in the absence of pregnancy indicate a physiological or pathological disturbance of this well-organized mechanism. Although the actual cause of POI is uncertain, the involvement of environmental and genetic variables in this condition has been demonstrated (Liu et al., 2023a).

POI is distinct from menopause in that it is a reversible ovarian syndrome that affects around 50% of POI patients. Furthermore, roughly 5%–10% of individuals with POI get pregnant and give birth to a healthy child following therapy (Nelson, 2009). There have been no reports of successful treatment yet. Hormonal treatment can only give brief relief for E2 deficiency. Other options, including egg transfer, are sometimes unfeasible owing to financial and ethical considerations (Huang et al., 2022a). Higher E2 levels and enhanced follicle growth, as well as an expanded population of antral follicles, indicate proper ovarian function, which eventually leads to a healthy pregnancy. Cellular differentiation, better vascular remodeling, decreased apoptosis, and upregulation of antioxidant molecules all contribute to the recovery of ovarian tissue structure and function (Yan et al., 2022a). Numerous investigations demonstrate that EXOs have therapeutic advantages in POI (Table 3; Tesarik et al., 2021). Exosome therapy with MSCs prior to chemotherapy can maintain ovarian health and safeguard fertility by overexpressing ATP synthase-binding cassette carriers, including ABCB1b (Park et al., 2024). As key players in folliculogenesis, oocyte maturation, steroidogenesis, and ovulation, microRNAs (miRNAs) are essential regulators of ovarian function (Ghasroldasht et al., 2025; Nazdikbin Yamchi et al., 2023). EXOs affect several ovarian functions, including tissue remodeling, apoptosis, and division of cells. PCOS, POF, and gynecological cancers have all been related to dysregulation of miRNAs (Nouri et al., 2022).

**TABLE 3:** Investigation of exosomes therapeutic actions in POI.

Type of exosomes	Preparation method	Size	Model	Outcomes	Reference
ESCs-EXOS	Differential centrifugation and ultracentrifugation	50–75 nm	*In vivo*: Mice	Enhanced granulosa cell proliferation and increased phosphorylated PI3K and AKT expression.	Liu et al. (2020c)
miR-126-3p-HucMSCs-EXOs	Ultracentrifugation	10–100 nm	*In vitro*: Rat OGCs	Displayed both pro-angiogenic and anti-apoptotic effects	Qu et al. (2022)
HucMSC-Exos	Differential centrifugation and ultracentrifugation		*In vitro*: Mice OGCs *In vivo*: Mice	Re-established hormone levels for the ovaries, leading to enhanced functionality and multiplication.	Li et al. (2021b)
MenSCs-EXOs	Ultracentrifugation		*In vivo*: Rats	Increased the activity and reduced the death of VCD-induced granulosa cells *in vitro*. improved POI prognosis, restored ovaries bioactivities, and increased GCs activity.	Song et al. (2023)
HUC-MSCs EXOs	Differential centrifugation and ultracentrifugation	141.6 nm diameter	*In vivo*: Mice	Restored ovarian phenotype and function, increased proliferation	Ding et al. (2020)
MenSCs-EXOs	Ultracentrifugation and ultrafiltration	128 nm diameter	*In vivo*: Rats	MenSCs-EXOs exposure boosted granulosa cell proliferation in primordial and primary follicles	Zhang et al. (2021b)
MSC-derived EXOs	Centrifugation	NA	*In vivo*: Mice	Restored estrous cycle and serum hormone levels	Park et al. (2023)
BMSCS EXOs- miR-144-5p	Differential centrifugation	NA	*In vivo*: Rats	Effectively prevented CTX-induced POF and improved repair by inhibiting GC apoptosis through PTEN targeting.	Yang et al. (2020a)

BMSCs, Bone marrow mesenchymal stem cells; CTX, cyclophosphamide; ESCs, embryonic stem cells; HucMSC-EXOs, Human umbilical cord mesenchymal stem cell-derived-exosomes; HucMSCs, human umbilical cord mesenchymal stem cells; MenSCs, Menstrual blood stromal cells; MSCs, Mesenchymal stem cells; OGCs, ovarian granulosa cells; PI3K, phosphoinositide-3-kinase.

HUCMSCs-EXOs stimulate primordial follicles by transporting functioning microRNAs. Intrabursal injection of HUC-MSCs-EXOs into elderly female mice resulted in enhanced oocytic synthesis and better performance, reversing impaired fertility (Yang et al., 2020b). *In vitro,* MSCs-EXOs dramatically increased cell proliferation and estrogen release while inhibiting apoptosis and pyroptosis. EXO’s therapy corrected erratic estrous cycles, reversed apoptosis of the follicles, and raised the conception rate and quantity of pups in POI mice (Xie et al., 2024).

**FIGURE 5:**
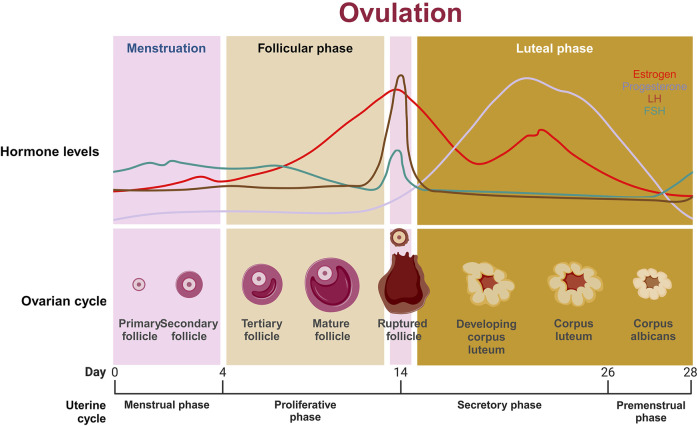
Ovarian Hormones and folliculogenesis throughout the human menstrual cycle.

Human amniotic epithelial cells-EXOs may recapture ovarian functioning in chemotherapy-triggered POF mice by transporting miRNAs (Zhang et al., 2019a). MiR-21 transported by HUCMSCs-derived EXOs might suppress LATS1, lowering phosphorylated LOXL2 and YAP, and, as a result, increasing estrogen release in ovarian granulosa cells (Cai et al., 2022). Human endometrial stem cells (EnSCs)-EXOs dramatically increased ovarian granulosa cells (OGCs) proliferation and function via modulating the Hippo signaling system. These results add to our comprehension of EnSC-EXOs’ role in ovarian function recovery (Wang et al., 2024b). Serum sex hormone levels returned to normal following embryonic stem cell (ESCs)/EXOs transplantation. Furthermore, the number of follicles grew dramatically, while the amount of apoptotic cells dropped (Figure 5). The *in vitro* tests showed that ESCs-EXOs could dramatically boost granulosa cell proliferation and phosphorylated PI3K and AKT expression levels. Additionally, the beneficial impact on multiplication and the antagonistic influence on apoptosis found in GCs were clearly reduced when the PI3K/AKT system was blocked (Liu et al., 2020c). In a rat POF model, application of miR-126-3p-hUCMSCs- EXOs elevated E2 and AMH concentrations, raised body and female reproductive organs masses and follicle numbers, and decreased FSH (Qu et al., 2022).

*In vitro,* EXOs administration dramatically increased the activity of granulosa cells caused by 4-vinylcyclohexene diepoxide (VCD) while inhibiting apoptosis (Song et al., 2023). Menstrual stem cells (MenSCs) stimulated the division of granulosa cells, as well as prevented follicle apoptosis. In a rat model of POI, MenSCs-EXOs transplantation successfully increased follicle formation and augmented estrous cycle and normal levels of sex hormones, leading to a better live birth outcome (Zhang et al., 2021b).

Regarding the assessment of long-lasting impacts, MSCs-administered mice revealed that 60%–80% of the samples became pregnant in the second cycle of breeding. In contrast, mice that received EXOs became impotent again in the second cycle of reproduction (Park et al., 2023). BMMSCs-derived EXOs suppress degeneration of ovarian follicles in cyclophosphamide (CTX)-administered rats by transporting miR-144-5p, which could be transported to CTX-affected OGCs and reduce GC death (Yang et al., 2020a).

In POI rats, amniotic fluid EXOs transplantation may help ovarian function via activating the TGF-β/Smads signaling system (Nazdikbin Yamchi et al., 2023). In order to compare the fertility restoration, parallel breeding studies were also carried out. Comparing the MSCs-treated and EXOs-treated groups to the untreated POI mice, the former showed restored blood hormone levels and estrous cycles. After treatment, the group treated with MSCs had a pregnancy rate of 60%–100%, whereas the group treated with EXOs had a pregnancy rate of 30%–50%. Remarkably, in terms of long-term impacts, the mice that received MSCs continuously maintained a 60%–80% conception rate over a second breeding cycle, whereas the group treated with EXOs relapsed into infertility during the same period (Park et al., 2023).

Thirty women with reduced ovarian reserve who refused the egg donation process participated in a prospective, randomized, comparative research by Navarro et al. There were three trial groups, each with ten patients: the first received saline solution, the second PRP, and the third platelet-derived autologous EXOs. Women in the autologous Exosome group showed improvements in female reproductive factors such as FSH, LH, Estradiol, Anti-Müllerian hormone, and antral follicle count, in addition to edema of more oocytes acquired in Metaphase II, higher fertilization percentage, frozen embryos, and confirmed pregnancies. Autologous EXOs for ovarian biostimulation may provide a safe and effective treatment for lowering indicators of poor ovarian reserve (Navarro et al., 2025). In conclusion, research has shown the clear advantages of exosome therapies for the management of POI and in regenerative medicine. Nevertheless, there are not enough preclinical studies showing exosome therapy’s effectiveness and safety for POI.

### 6.4 Ashermann syndrome (AS)

It is a gynecological illness that was originally reported by Israeli physician Joseph Asherman in 1948 (Khan, 2023). It is a complex illness that involves the partial or total loss of the uterus and/or cervical canals. It is a hotly debated issue due to its significant impact on both reproductive outcomes and gynecological disorders (Liu et al., 2020a). According to recent findings, damage to the endometrium is the primary driver of intrauterine adhesion development (He et al., 2022; Dong et al., 2025). If neglected, these adhesions can produce several manifestations, from mild to catastrophic (Tarafdari et al., 2023).

Recovering the uterine cavity’s dimensions and form, limiting adhesion recurrence, encouraging the endometrium’s regeneration and repair, and reestablishing regular reproductive processes are the goals of treatment for Asherman syndrome. Numerous surgical procedures have been described within the past century (Saribas et al., 2020; Khan, 2023). Following surgery, the uterine cavity can be restored and the endometrium can be regenerated, allowing all three patients to resume regular menstruation (Tan et al., 2021). However, patients’ postoperative hysterosalpingography (HSG) findings showed persistent endometrial scarring. In situations when the uterus is completely obliterated and there are no markers to help the surgeons separate the cavity walls, the researchers concluded that this procedure has shown promising outcomes in restoring the integrity of the uterus (Katre et al., 2024). However, to confirm the effectiveness of this method, many patients must undergo longer-term follow-up evaluations. This therapy should only be used in the most severe circumstances, and patients should have received counseling on the risks of scar rupture during subsequent pregnancies, the chance for bleeding with a hysterectomy, and the difficulties of a laparotomy (Zhang et al., 2023c; Tsiampa et al., 2024).

Newton et al. (1989) and Chapman and Chapman (1996) have reported on laser vaporization surgery, employing Nd-YAG and KTP lasers. In the latter technique, the necrosis depth has been reported to be low, ranging from 1 to 2 mm. Although the technique has been employed in outpatient settings with CO2 distension, it is only applicable to individuals who have filmy intrauterine adhesions. Nowadays, hysteroscopic adhesiolysis seldom ever uses this technique (Echeng et al., 2024). While Asherman’s syndrome was first recorded over a century ago, and several preventative measures have been developed, a viable non-invasive treatment for preventing adhesion recurrence must be presented and proven (Table 4; Ghajari et al., 2023).

**TABLE 4:** Exosomes benefit in Ashermann syndrome therapy.

Type of EXOs	Cargo	Preparation technique	Size	Model	Outcomes	Reference
MSCs/MSCs-EXOs		Ultra-centrifugation	120–400 nm in diameter	*In vivo*: Rats	Minimized concentrations of proinflammatory TNFα and boosted the release of anti-inflammatory IL-10, as well as endometrial receptivity cytokines VEGF and LIF.	Mansouri-Kivaj et al. (2023)
uterine-derived MSCs-EXOS		Immunoprecipitation	40–100 nm	*In vivo*: Rats	Enhanced vascularization and proliferation in uterine tissue, as well as reduced fibrosis faster than MSCs.	Saribas et al. (2020)
BMSCs-EXOs		Immunoprecipitation	130 ± 11 nm.	*In vivo*: Rabbits	TGF-b1 can reverse the endothelial-mesenchymal transition (EMT) in rabbits.	Yao et al. (2019b)
ADSC-EXOs- loaded PEG-Ag hydrogel	ADSC-EXOs	Differential centrifugation	50–100 nm in size	*In vitro*: HUVEC *In vivo*: Rats	*In vitro*: improved their angiogenic potential. *In vivo*: promoted endometrial regeneration	Lin et al. (2021b)
ADSC-EXOs		Differential ultracentrifugation	30–200 nm	*In vitro* *In vivo*: Rats	Restoring endometrium to normal shape, decreasing fibrotic mediators.	Zhao et al. (2020)
UCMSC-EXOs		Differential ultracentrifugation	50–150 nm	*In vitro:* endometrial stromal cells (ESC)	Alleviated TGFβ1-induced endometrial fibrosis.	Li et al. (2023b)

ADSC-EXOs, Adipose stem cell-derived exosomes; BMSC, bone marrow mesenchymal stem cell; ESC, endometrial stromal cells; IL, interleukin; LIF, leukemia inhibitory factor; MSCs, Mesenchymal stem cells; PEG, poly ethylene glycol; TGF, transforming growth factor; UCMSC-EXOs, umbilical cord mesenchymal stem cell-derived exosomes; VEGF, vascular endothelial growth factor.

Numerous paracrine factors involved in angiogenesis and regeneration are found in EXOs. EXOs are very interesting in the field of regenerative medicine since they include a wide range of materials, including non-immunogenicity (Mansouri-Kivaj et al., 2023; Hou et al., 2025). EXOs miR-122 can boost endometrium rejuvenation and repair of conceiving capability in mice (Chen et al., 2023).

Although there are EV20K and EV110K subpopulations, EV110K populations are in fact often smaller than EV20K populations (Hosseinkhani et al., 2020). Mansouri-Kivaj et al. mechanically damaged adult rat uteri to construct an AS model. A homogenous population of BMMSCs, MSCs, or MSCs-derived EXOs subpopulations (EV20K and EV110K) was then administered to the mice right away. MSCs and EXOs transplantation helped to heal the endometrium and promote female fertility, most likely by inhibiting extensive fibrotic and inflammatory reactions, increasing endometrial cell division, and regulating mediators associated with endometrial receptivity. BMMSCs outperformed traditional MSCs in terms of reproductive functionality restoration. Furthermore, EV20K is more affordable and viable for the avoidance of AS than traditional EXOs (EV110K) (Mansouri-Kivaj et al., 2023).

MSCs and exosome therapies improved uterine tissue growth and vascularization. MSCs and EXOs treatment raised MMP-2 and MMP-9 transcription, but TIMP-2 concentrations dropped. MSCs and exosome treatments boosted multiplication and vascularization while decreasing fibrosis in the uterus, with better results in EXOs-treated groups (Saribas et al., 2020). BMMSCs-derived EXOs, like BMMSCs, can heal wounded endometrium and could counteract EMT in rabbit epithelial ECs generated by TGF-β1. BMMSCs-EXOs may stimulate endometrial healing via the TGF-β1/Smad system (Yao et al., 2019b). ADSCs-EXOs Laden PEG Hydrogel has a remarkable neovascularization-promoting impact, increasing HUVECs division and tube formation by 1.87 and 2.2 times. This platform enhanced vascularity and tissue regeneration while blocking fibrosis *in vitro* and *in vivo* (Lin et al., 2021b).

Adipose stem cells EXOs (ADSC-EXOs) therapy in the IUA model preserved typical uterine morphology, accelerated endometrial rejuvenation and restructuring of collagen, increased levels of integrin-β3, LIF, and VEGF, and increased responsiveness of the rejuvenated endometrium (Zhao et al., 2020). Umbilical cord stem cells EXOs (UCMSCs-EXOs) can stop endometrial cell fibrosis via modulating the miR-145-5p/ZEB2 axis, suggesting a possible innovative method to enhance endometrial healing (Li et al., 2023b).

Tan et al. extracted BMSC-Exo using the magnetic bead affinity technique and examined its biological makeup. In this study, the exosome-specific proteins CD9, CD63, and CD81 were expressed by BMMSCs-EXOs. The contents may be transported into the target cells by BMMSCs-Exo. Both *in vitro* and *in vivo*, BMMSCs-Exo can support endometrial healing. Overexpression of miR-29a in BMMSCs-Exo may decrease αSMA, Collagen I, SMAD2, and SMAD3 (Tan et al., 2020).

### 6.5 Preeclampsia

Preeclampsia is one of the “enigmatic obstetrical syndromes” in which several, sometimes interconnecting pathologic events activate a similar pathway that includes endothelial cell stimulation, intravascular inflammatory processes, and syncytiotrophoblast stress (MacDonald et al., 2022). Preeclampsia is a hypertensive pregnancy condition that, if detected and treated early, can significantly reduce the risk of fetal mortality (Lin et al., 2022).

Nowadays, invasive laboratory testing and clinical signs like proteinuria and hypertension are used to diagnose preeclampsia. Establishing preventative measures to lower the incidence and severity of preeclampsia (PE) and its related consequences requires the development of methods for earlier identification of people who are susceptible to PE (Verlohren and Dröge, 2022).

Kim et al. used maternal urine and urinary EXOs to study the function of soluble proteins and EXOs in noninvasively detecting preeclampsia. They found that urine as-is had larger quantities of soluble proteins than urinary EXOs, including placental growth factor (PlGF) and fms-like tyrosine kinase-1 (sFlt-1). The sensitivity of the sFlt-1/PlGF ratio proved to be 1.5 times greater in tests using urine-derived EXOs and 4.0-fold greater in urine testing when compared with commercial blood tests. Their research presents encouraging opportunities for the early and non-invasive detection of high-risk patients who may develop preeclampsia, enabling all-encompassing preventative care (Kim et al., 2024).

Salomon proposed that measuring the amount of placenta-derived EXOs in maternal blood, as well as the expression of hsa-miR-486-1-5p and hsa-miR-486-2-5p, might help us monitor asymptomatic women who are at risk of developing PE (Salomon et al., 2017). Because placental protein 13 is essential for initial placental growth and the modulation of maternal immunoreaction via T-cell and macrophage apoptosis, low quantities of this protein in EXOs may be significant for confirming the diagnosis of PE (Pillay et al., 2017). Additionally, syncytin-2, an immunosuppression protein generated from EXOs, can prevent T lymphocyte and NK cell activation via the Fas ligand and PD-L1 (Mincheva-Nilsson and Baranov, 2014). STBs often use exocytosis to create placenta-derived EXOs, which are then released into the mother’s bloodstream. By activating maternal lymphocytes (which leads to the detection of paternal placental antigens) and inducing the apoptosis of trophoblasts through exosome-driven secretion of FasL, STB-derived EXOs play a role in immunoregulation during pregnancy and contribute to the pathophysiology of PE (Mincheva-Nilsson, 2021; Jaremek et al., 2021; Yu et al., 2022).

**FIGURE 6:**
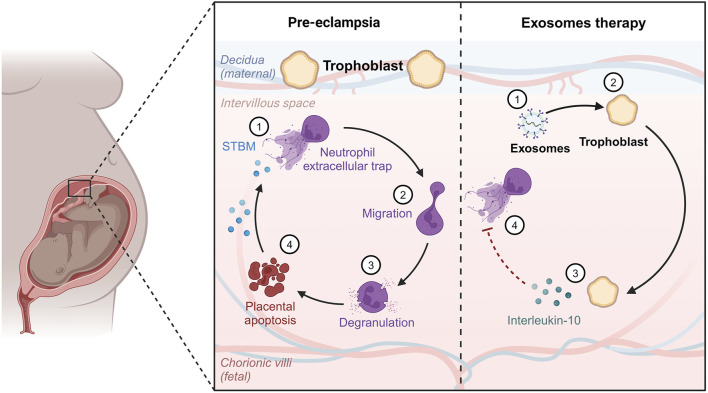
Exosomes therapy in pre-eclamptic placenta.

EXOs play a critical part in fetoplacental development in normal pregnancies (Figure 6; Tantengco et al., 2021). In this regard, Tang et al. effectively collected HucMSCs-EXOs which could partly reverse Soluble fms-like tyrosine kinase-1 (sFlt-1) triggered HUVECs dysfunction *in vitro*. The addition of HUCMSCs-EXOs partially restored the reduced eNOS protein expression seen in OV-sFlt-1-HUVECs. Endothelium cellular migration may be facilitated by MSCs-EXO’s delivery of matrix metalloproteinase 2 (MMP2). It has been shown that EXOs MMP2 stimulates endothelial angiogenesis through the VEGF/Erk1/2 signaling system (Tang et al., 2019).

Versican (VCAN), which is abundantly expressed in tissues with metabolic activity, can drive angiogenesis and may rely on interactions with VEGF to affect the ECM’s assembly (Sagae et al., 2023). These proteins deposited in HUC-MSCs-EXOs were taken up by the vascular endothelium and stimulated angiogenesis, relocation, and cell division to repair compromised vascular tissues in animals that resembled preeclampsia. In mice, HUCMSCs-EXOs alleviated hypertension and improved fetal birth weight; furthermore, preeclamptic dams injected with these regenerated extensive placental vascularity (Chang et al., 2023).

In a different investigation, it was shown that EXOs miR-139-5p from HUCMSCs-EXOs accelerated trophoblast invasion and migration, activated the ERK/MMP-2 pathway, and blocked trophoblast apoptosis by decreasing protein tyrosine phosphatase expression, all of which improved PE manifestations in rats (Liu et al., 2020b). Furthermore, research found that the placenta of PE patients had greater amounts of Notch2, TIM3, and mTORC1 and decreased expression of miR-18b. By releasing microRNA-18b, which inhibits trophoblasts’ production of Notch2, HUC-MSCs-EXOs stimulated trophoblast motility. They also used HUCMSCs-EXOs in a rat model of PE and discovered that it helped pregnant animals with PE symptoms (Yang et al., 2021b). Additionally, it was discovered that by delivering miR-101 to trophoblasts and suppressing BRD4 expression, HUCMSCs-EXOs stimulated trophoblast recruitment and penetration. Under hypoxic circumstances, MSCs-EXOs stimulated trophoblast migration and invasion as well as autophagy and trophoblast multiplication (Cui et al., 2020). In addition to suppressing trophoblast inflammation in hypoxic circumstances, Jiang et al. found that HUC-MSCs-EXOs enhanced the growth, migration, and spreading of hypoxic trophoblasts and decreased FSTL3 expression via transferring miR140-5p (Jiang et al., 2022). Amniotic MSCs-derived EXOs promote trophoblast proliferation by blocking the EZH2/mTOR signaling pathway, which in turn enhances autophagy in trophoblasts (Chu et al., 2020).

Even though EXOs have much promise for therapeutic uses, their restricted capacity to target in animal trials results in issues such as short half-lives and decreased therapeutic effectiveness. By directly altering EXOs without requiring cell modification, biochemical engineering provides a quicker, easier, and more efficient method to increase certain exosome secretion (Xu et al., 2021).

King et al. investigated the possibilities of tumor-homing peptide iRGD, which attaches to the placental surface in mice and humans in a selective manner without obstructing normal development. As a result, iRDG-EXOs may have essential proteins or genes that target the placenta preferentially and are essential for treating PE (King et al., 2016). In addition to improving our knowledge of the pathophysiology of preeclampsia, additional investigation into particular chemicals found in EXOs makes it possible to find more sensitive and precise biomarkers for the start of the condition. It has potential as a therapy for preeclampsia as well.

### 6.6 Gynecological cancers

Gynecological carcinomas are cancers of the female reproductive tract, which includes the uterus, ovary, fallopian tube, and placenta (Kataki et al., 2023). These illnesses constitute an international health concern for women (Sun et al., 2024). Gynecological malignancies are predicted to cause around 17 million disability-adjusted life years (DALYs), accounting for one-sixth of all cancer-related DALYs in women (Kocarnik et al., 2022).

Ganesh et al. treated HeLa cells with Allyl Isothiocyanate (AITC) and looked at the effect of miR16-enriched EXOs on human fibrosarcoma HT1080 cells. When EXOs were grown with fibroblasts, miR-16 transcription rose within the cells. AITC-exposed HeLa EXOs raised the Bax/Bcl2 ratio while decreasing PCNA, HIF-1α, SDF-1α, IL-6, and p22phox expression in fibroblasts. Knocking down miR16 in fibroblasts reduced AITC-induced increases in the Bax/Bcl2 ratio while restoring production of VEGF, PCNA, HIF-1α, SDF-1α, IL-6, and p22phox. These findings highlight the promise of AITC-mediated EXOs miR16 enrichment as an efficient strategy to suppress cancer growth and progression, as well as a novel possibility for the treatment of cancer (Ganesh et al., 2025).

#### 6.6.1 Ovarian cancer (OC)

**FIGURE 7:**
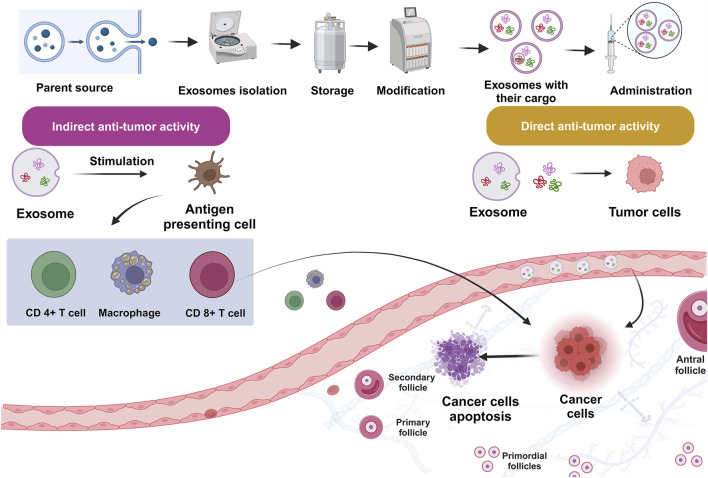
Exosomes therapy in management of ovarian cancer.

OC is the primary cause of mortality among females who receive a diagnosis of gynecological cancer (Menon et al., 2021). In addition, it is the seventh leading cause of death among women worldwide (Gaona-Luviano et al., 2020). EXOs have also been found to influence the tumor immunological milieu and subsequent immune responses, including presenting antigens, movement, metastasis, and tumor infiltration (Guo et al., 2022). Despite accurate diagnosis and early treatment approaches, the outcome for OC patients remains poor since chemotherapy’s efficiency is restricted by resistance and off-site effects (Cabasag et al., 2022). EXOs are widely regarded as reliable carriers thanks to their capacity to circumvent current pharmacokinetic issues. EXOs, unlike other nano vectors, may bypass the endosomal and lysosomal routes and transfer their cargos straight into the cytoplasm of target cells (Figure 7; Table 5; Li and Wang, 2017).

**TABLE 5:** Exosomes in management of ovarian cancer.

Type of exosomes	Cargo	Preparation metho	Dimensions	Model	Outcomes	Reference
miR-199a-3p-EXOs		Ultracentrifugation and density gradient ultracentrifugation	100 nm	*In vitro*: OC cell lines (CaOV3; SKOV3; OVCAR3)	Inhibited cell proliferation and invasion significantly reduced peritoneal spread	Kobayashi et al. (2020)
ThP-1 cells EXOs/IDEM	DOXO	Ultracentrifugation	EXOs: 112 ± 14 nm IDEM 177 ± 19 nm.	*In vitro*: SKOV-3 ovarian cancer cells	Targeted cytoxicity	Pisano et al. (2020)
eNK-EXOs		Differential ultracentrifugation	∼80 nm	*In vitro*: SKOV3 cells	Selective cytotoxicity and anti-proliferative impact.	Luo et al. (2023a)
HENPs	TP and miR497	Ultracentrifugation and Ultrafiltration	104 ± 11 nm	*In vitro*: SKOV3 cells *In vivo*: Mice	Significantly enhancing tumor cell apoptosis without any negative effects *in vivo*.	Li et al. (2022c)
OCSCs-EXOS- miR-4516		Ultra-high-speed centrifugation	30–150 nm	*In vitro*: SKOV3/DDP *In vivo*: mice		Pan et al. (2024a)
MSCs-EXOs	miR-424	High-speed centrifugation	30–120 nm	*In vitro*: SKOV-3, HO8910, A2780 and HUVECs.	Blocked ovarian cancer cell growth, immigration, and infiltration.	Li et al. (2021a)
hUC-MSC-derived EXOs miR-146a		Differential ultra centrifugation	∼100 nm	*In vitro*: A2780 and SKOV3	Reduced ovarian cancer cell proliferation and resistance to chemotherapy.	Qiu et al. (2020)
ADSCs -EXOs		Immuno precipitation	70 nm and 100 nm	*In vitro*: A2780 and SKOV-3	Reduced in viability and proliferation.	Reza et al. (2016)

ADSCs, Adipose stem cells; eNK-EXOs, exosomes derived from expanded natural killer cells; EXOs, Exosomes; HENPs, Hybrid exosomes nanoparticles; HucMSCs, human umbilical cord mesenchymal stem cells; HUVECs, Human umbilical vein endothelial cells; IDEM, immune derived exosome mimetics; MiRNAs, Micro ribonucleic acids; MSCs, Mesenchymal stem cells; OV, ovarian cancer; OCSCs, ovarian cancer stem cells; TP, triptolide.

The majority of OC patients respond sensitively to the recommended platinum and paclitaxel (PTX) treatment. Regretfully, the majority of patients die from increasingly chemotherapy-resistant illness and relapse within 6–12 months (Cho and Shih, 2009). In pharmaceutical-resistant human ovarian cancer cells, the lysosomal proteins ATPase copper-transporting alpha and beta (ATP7A and ATP7B), which are potential CIS-export carriers, can increase the EXOs transfer of platinum (Safaei et al., 2005).

Berry bioactive agents show potential for cancer prevention and treatment. Aqil et al. investigated the inhibitory actions of berry anthocyanidins (Anthos) on the division of ovarian cancer cells. EXOs Anthos (EXOs Anthos) had a much higher anti-proliferative efficacy against ovarian cancerous cell growth and reduced tumor development more effectively than other groups. Paclitaxel (PAC) is often effective in treating patients with cisplatin-resistant cancers. Finally, they found that the amalgamation of Anthos and PAC lowered PgP levels in OVCA432 cells in a dosage-dependent way. The amalgamation of ECOs@PAC and EXOs @Anthos demonstrated considerably increased anticancer efficacy against A2780 tumor xenografts (Aqil et al., 2017).

MiR-199a-3p-Exo significantly reduced peritoneal spread in the OC mouse model and decreased c-Met transcription, ERK phosphorylation, and MMP2 levels in cancers (Kobayashi et al., 2020). Pisano et al. presented immunologically derived EXOs, Mimetics (IDEM) from monocytic cells as an innovative technique for targeting and killing ovarian cancerous cells. Drug uptake by IDEM was better than natural EXOs, revealing IDEM’s advantage in decreasing negative reactions while increasing cytotoxicity in the targeted tissues by administering a lesser dose of the chemotherapy (Pisano et al., 2020). Moreover, eNK-EXOs can be selectively absorbed by SKOV3 cells and are cytotoxic to OC cells. Additionally, eNK-EXOs loaded with cisplatin enhance OC cells’ responsiveness to cisplatin’s anti-proliferative impact. In addition, they could stimulate NK cells from the inhibitory tumor micro-environment (Luo et al., 2023a).

MSCs-derived EXOs transport miR-424, which downregulates MYB, hence inhibiting ovarian cancer development and angiogenesis. Thus, this work provides a possible predictive indicator and treatment approach for ovarian carcinoma (Li et al., 2021a). HUCMSCs-EXOs inhibited cell proliferation and chemo resistance in OC (Qiu et al., 2020). In addition, hAMSCs-derived EXOs increased apoptosis signaling by increasing several pro-apoptotic mediators, while minimizing the anti-apoptotic protein BCL2. More precisely, cancerous cells showed decreased survivability after being treated with fresh or protease-digested EXOs (Reza et al., 2016).

EXOs have lately been exploited as medication delivery vehicles due to their inherent benefits. To enhance patient outcomes with platinum-resistant ovarian cancer, innovative medication delivery strategies are required. Sonication disrupted the EXOs’ membrane, allowing for great loading efficiency. Loading cisplatin into M2 EXOs significantly improved its cytotoxicity in chemo-resistant A2780/DDP cells (1.7×) and pharmaceutical-responsive A2780 cells (1.4×) (Zhang et al., 2020c).

More studies are needed to ascertain the diagnostic sensitivity and selectivity of exosome analysis and miRNA expression profiling in early-stage ovarian cancer, despite some studies suggesting its diagnostic promise (Berkel and Cacan, 2021). For instance, Todeschini et al. examined two cohorts of 168 patients with stage III–IV HGSOC and 65 healthy controls. They showed that miR-1246 has clinical potential as a diagnostic biomarker for High-grade serous ovarian carcinoma (HGSOC), as evidenced by the significantly higher expression of miR-1246 in serum samples of HGSOC patients than in healthy individuals. In order to find potential diagnostic miRNAs, this work employed a unique microarray data normalization technique. RT-qPCR was then utilized to validate the signatures. According to the study, HGSOC patients had considerable overexpression of miR-1246, miR-595, and miR-2278. MiR-1246 had the best detection capability, with an 87% diagnostic sensitivity, 77% specificity, 84% accuracy, and 0.89 AUC (Todeschini et al., 2017).

Likewise, there have been suggestions regarding the clinical potential of EXOs in ovarian cancer. Zhang et al. examined the function of four exosome proteins Lipopolysaccharide Binding Protein (LPB), Fibrinogen Gamma Chain (FGG), Fibrinogen Alpha Chain (FGA), and Gelsolin (GSN) as diagnostic biomarkers by comparing plasma samples from 40 stage III or IV EOC patients to 40 healthy controls (Zhang et al., 2019b). According to this study, the ovarian cancer group had considerably lower levels of FGG and LBP and significantly higher levels of FGA and GSN. With an AUC of 0.8459, FGA had the best diagnostic sensitivity of the four choices. In a different research, Schwich et al. found that the plasma circulating EXOs of 78 EOC patients (63 stage III-IV and 7 stage I-II) had seven times higher HLA-G levels (mean 14.3 ng/mL) than healthy controls (1.9 ng/mL) (Schwich et al., 2019). As a result, research on miRNA and exosome analysis has been focused on patients with advanced ovarian cancer thus far; further studies are required to clarify their diagnostic value for early-stage illness.

Since EXOs cannot reproduce and are not mutagenic, they are a safer option for biological carriers than viral vectors or cell therapy. Therefore, there are not many regulatory worries about toxicity or the emergence of neoplasia. Experiments using *in vivo* EXOs therapy have shown little to no harm (Elsharkasy et al., 2020). EXOs generated from HEK293T cells are not harmful when given to mice systemically (Zhu et al., 2017), while siRNA-engineered EXOs remain non-toxic even after being given to animals repeatedly (Kamerkar et al., 2017). Exosome safety is demonstrated by these investigations as well as several other tests. Nonetheless, there are major differences between complement activation-related pseudoallergy (CARPA) in big animals and small animals (rodents) (Dézsi et al., 2014), Exosome safety is demonstrated by these investigations as well as several other tests. However, safety trials in rodents require cautious interpretation since the CARPA in small animals (rodents) differs fundamentally from that in big mammals. When administered as an intramuscular vaccination, serum-derived EXOs from virus-infected pigs do not exhibit any CARPA-related toxicities (Montaner-Tarbes et al., 2018).

#### 6.6.2 Cervical cancer

Cervical cancer is one of the most frequent female carcinomas and the leading cause of cancer-associated mortality in females globally (Castle et al., 2021). As a prominent therapeutic technique in cervical cancer, chemotherapy can increase the survival rate of patients by forcing cancerous cells to undergo apoptosis (Shelley et al., 2021). However, due to the action of chemotherapy medications, cancerous cells eventually develop resistance against chemotherapy (Sherer et al., 2022).

Numerous processes enable cancer cells to withstand the cytostatic and cytotoxic effects of medications, which leads to drug resistance in these cells. The primary cause of this is the membrane protein of the ATP-binding cartridges, which can extract harmful substances from the intracellular medium (Chen et al., 2020). The varied activity of cytochrome P450, which is brought on by different genetic variations of the CYP gene family, is another factor contributing to drug resistance (Ding et al., 2018). These genetic variations are connected to cancer cells’ efficient drug metabolism, which lessens the cytotoxic effects of medications (Abbas et al., 2022). Furthermore, the majority of anticancer medications target DNA impairment, which is repaired via various cancer’s efficient mechanisms. Vesicles have been implicated in the failure of cytostatic therapies, according to studies (Bhuia et al., 2023; Shi et al., 2024). The microenvironment of cancerous lesions is rich in EXOs, which have a role in the invasion, metastasis, angiogenesis, and treatment resistance of these malignancies (Mashouri et al., 2019; Sahebi et al., 2020). EXOs miR-651, produced from cancer, specifically targeted ATG3 and inhibited cisplatin resistance, suggesting that it may be an effective therapy.

Initiation of cancer cell ferroptosis has been advocated as a therapy for a variety of types of cancers. Tumor-associated macrophages (TAMs) serve an essential function in increasing tumor malignancy and therapeutic resistance (Deng et al., 2022). Luo et al. observed that TAMs-derived EXOs carrying miR-660-5p into cervical cancer cells might inhibit arachidonate 15-lipoxygenase (ALOX15) transcription and hence attenuate ferroptosis. The process is then hampered by this miR-660-5p, which suppresses the production of ALOX15 in cancer cells, a crucial enzyme involved in ferroptosis. The paper also emphasizes the findings’ clinical significance, indicating that modifying the expression of miR-660-5p or focusing on TAMs may be viable treatment approaches for cervical cancer (Luo et al., 2023b).

It is well known that microRNAs (miRNAs) play a significant role in the development of CC. It was discovered that the plasma EXOs of CC patients had decreased levels of MiR-423-3p. EXOs have been linked to macrophage polarization, and exo-miRNAs have been shown to be putative modulators of cancer development. EXOs miR-423-3p can reduce tumor growth and CC cell development by blocking macrophage M2 polarization. By targeting cyclin-dependent kinase 4 (CDK4) mRNA, miR-423-3p can control macrophage M2 polarization. It also suppresses the phosphorylation of the signal transduction and activation of transcription 3 (STAT3) via CDK4 to reduce the production of interleukin 6 (IL-6) (Yan et al., 2022b).

#### 6.6.3 Endometrial cancer

As stated by Global Cancer Statistics 2020, endometrial cancer (EC) is the second most prevalent malignancy of the female genital tract and the sixth most prevalent female cancer, with significantly greater rates of incidence in developed countries than in developing countries (Sung et al., 2021). EC is mostly an illness of postmenopausal women, with an average age of onset of 65 (Karkia et al., 2025).

Through EXOs, endometrial cancer cells can transfer short regulatory RNAs to endometrial fibroblasts (Bian et al., 2024). EXOs produced from endometrial cancer cells contained EXOs miR-133a, which was transferable to normal endometrial cells (Shi et al., 2020). By controlling the miR-381-3p/E2F transcription factor 3 (E2F3) axis, EXOs lncRNA deleted in lymphocytic leukemia1 (DLEU1) generated from endometrial malignancies enhanced the migrative and invasive capabilities of endometrial cancerous cells (Jia et al., 2020). However, the MSCs EXOs miR-499 decreased tumor development and angiogenesis *in vivo* and prevented the division of endometrial cancer cells and tube development of endothelial cells *in vitro* (Jing et al., 2020).

**FIGURE 8:**
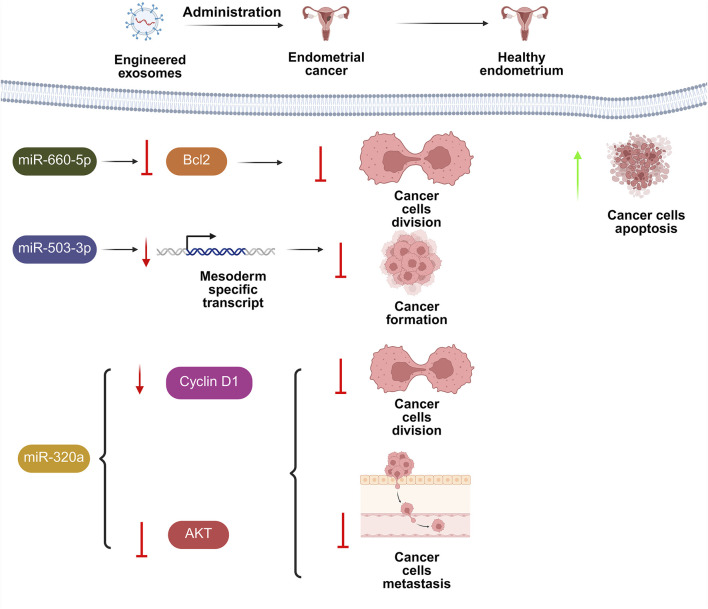
Application of engineered exosomes in management of endometrial cancer.

Cancer-associated fibroblasts’ EXOs lncRNA NEAT1 promotes the growth of endometrial cancer through the STAT3/YKL-40 signaling pathway controlled by miR-26a/b-5p (Fan et al., 2021). In conclusion, the impact of EXOs released by various cell types on the development of endometrial cancer is diverse. EXOs, which demonstrated promise in cancer treatment since they act as drug delivery vehicles and may influence tumor growth. They can transport therapeutic materials, including RNA, proteins, and medicines, targeting endometrial cancerous cells and potentially improving the efficacy of current treatments while reducing adverse effects (Figure 8; Song et al., 2021).

HUCMSCs-derived EXOs are regarded as ideal instruments for target-based treatments. HUCMSCs can convey engineered EXOs overexpressing tumor-suppressor miRNAs to EC cells, inhibiting their growth. Utilizing this technique, Li et al. targeted EC cells with miR-302a overexpressing EXOs, which inhibited their division and migration by lowering cyclin D1 levels and deactivating the AKT signaling (Li et al., 2019). Additionally, these EXOs decreased the levels of mesoderm-specific transcript (MEST) in EC cells, inhibiting tumor formation (Pan et al., 2022).

The most common cause of death from endometrial cancer (EC) is still metastasis. EXOs have been used in treatment plans due to their biological role and regenerative qualities. Numerous malignancies have reduced SERPINA5 expression, which is associated with invasion and migration of tumor cells (Fan et al., 2022; Yang et al., 2024a). Exogenous SERPINA5 loading of EXOs may be a unique treatment method for metastatic EC, according to Song et al., who also observed that low levels of SERPINA5 transcription are associated with decreased survival outcomes in EC (Song et al., 2022).

The lining of the uterus is impacted by endometrial cancer. EXOs are a promising option for treating cancer because of their endogenous action, intrinsic targeting, and capacity to interact with the host defense system (Rodolakis et al., 2023). These advantages suggest that MSCs’ EXOs laden with paclitaxel and carboplatin may function similarly to immune cells in the fight against cancer.

In comparison to normal endometrium, Ma et al. discovered that carboplatin (Car)/paclitaxel (Pac)@MSCs EXOs downregulated endometrial cancer (EC) cells. The effects of varying dosages of Car-Pac@EXOs on ECC-1 and HEC-1A EC cells were investigated *in vitro*. EXOs from Car-Pac@ MSCs caused apoptosis in EC cells. By decreasing MMP-2 expression through Rac1/NF-κB signaling, the formulation of Car-Pac@ MSCs EXOs decreased EC cell migration and invasion. The findings suggested that Car-Pac@ MSCs EXOs might be a useful tool for EC diagnosis and therapy (Ma et al., 2024).

## 7 Limitations in contemporary exosomes investigations

Notwithstanding the growing technological focus in the field of EXOs, investigations on EXOs in female reproductive health vary with regard to accuracy and systematic aspects (Yu et al., 2021; Liu et al., 2023b; Shen et al., 2023).

### 7.1 Isolation techniques

Isolation approaches differ in terms of their capacity to distinguish soluble elements from EXOs and the dimensions of the EXOs they can isolate (Le Gall et al., 2020). The use of varied methodologies resulted in inconsistencies in the size and components of EXOs evaluated in numerous EXOs investigations to date (Zhang et al., 2018). The viscosity and protein content of various fluids differ, requiring particular procedures depending on the biofluid or tissue under examination (Zebrowska et al., 2022). While a variety of solutions exist for harmonizing reconstruction and selectivity, research has thus far been impeded by a lack of approach standardization, which is crucial for reducing artifacts (Li et al., 2022d; Lopez de las Hazas et al., 2021). While differential ultracentrifugation is generally regarded as the hallmark of exosome separation, it has an enormous burden and frequently contains proteins and lipoproteins (Kverneland et al., 2023). While advances may be made by hyphenating distinct approaches of separation, such as ultracentrifugation and immunoaffinity capture, which draw on the capabilities of both the physical and biological worlds, it is important to consider the added workload and expense (Ciftci et al., 2023; Jang et al., 2023). Similarly, ultrafiltration has limits, despite being a common exosome separation method. For example, ultrafiltration is susceptible to obstruction and blockage, resulting in a shorter membrane lifespan and worse separation efficiency (Gao et al., 2022). EXOs can also bind to the membranes and become inaccessible for subsequent analyses, leading to decreased productivity and occasionally misinterpretation of outcomes from tests (Gao et al., 2022).

Furthermore, size-based separation of EXOs is complicated by the existence of a significant amount of nanoparticles (some non-vesicular) that are identical in dimensions to EXOs. SEC can produce extremely pure EXOs, but it requires specially designed machinery and is not easily scaled (Lu et al., 2021b). Since SEC is commonly conducted utilizing gravitational flow, vesicle shape and functionality are essentially intact, as is exosome biological activity. Furthermore, the SEC has remarkable repeatability. Nevertheless, its long run time restricts its capacity for large-scale scenarios (Shu et al., 2021).

Immunoaffinity capture is a powerful tool for separating EXOs of a given source as well as subpopulations of EXOs. However, as a fledgling area, the finest EXOs tags have yet to be developed (Pei et al., 2024). Given that only a portion of EXOs encoding the antibody-recognized protein is collected, yields are typically lower but considerably greater in purity than those separated using exosome physical features (Zhang et al., 2021a).

Underestimations and inaccurate findings may also result from disparities in antigen expression and regulation as the disease grows. Furthermore, the antigenic epitope can be inhibited or disguised. Although exosome precipitation is simple and exosome isolation can be completed in a single step, inconsistent yields, time-consuming sampling and cleaning processes, and a shortage of a suitable targeted isolation technique undoubtedly compromise the high quality of the separated EXOs, compromising subsequent analyses (Wang et al., 2021; Fernandes et al., 2025).

EXOs have been found to co-precipitate with other cellular components, including other extracellular vesicles, protein clumps, and even extremely abundant proteins, in a variety of biofluids such as plasma and serum. Furthermore, the varying viscosity and sample matrix need distinct exosome precipitation quality requirements, compromising precipitation technique harmonization. Despite tremendous advancements, none of the first-generation microfluidic devices are suitable for clinical trials due to difficulties like adaptability, assurance, and consistency (Huang et al., 2021; Cao et al., 2025).

Furthermore, some of the instruments have lengthy specimen pretreatments, while others developed for use with clean specimens have extremely low isolation efficacy. So far, all microfluidic devices produced use a single exosome quantification method, which results in limited yield or selectivity. Furthermore, their limited processing capabilities may impair further analysis due to inadequate levels of proteins and nucleic acids in the separated EXOs. As a result, contemporary exosome separation methods, despite significant advancements over the last decade, have brought an entirely novel array of obstacles to scientists in the area (Shirejini and Inci, 2022).

Another critical difficulty that should be addressed is the integration of exosome separation techniques into downstream analysis, which will eliminate the requirement of handling exosome extraction and subsequent examination independently. With integration, exosome analyses take a shorter period and include fewer stages, significantly enhancing the productivity and precision of exosome separation and analysis (Zhao et al., 2025).

### 7.2 Challenges in standardizing EXOs quantification

The literature describes a variety of approaches for isolating and quantifying EXOs (Miron and Zhang, 2024; Wu et al., 2021b). However, insufficient agreement on a ‘gold standard’ has emerged. Additionally, most researchers working in the domain of exosome investigation acknowledge that a uniform method of separation is essential for achieving greater comparability between findings and investigations (Wu et al., 2021b).

Fluorescence-activated cell sorting (FACS) is the most widely used method for exosome analysis 3. FACS offers the advantage of allowing cells from diverse sources to be compared in a single step using fluorescent labeling. FACS is not sufficiently accurate to distinguish particles smaller than 0.5 µm, but EXOs are typically between 30 and 120 nm in diameter (5), making it difficult to determine their dimensions (Akinduro et al., 2025).

Other methods for analyzing exosome particle size and shape include scanning electron microscopy (SEM) and transmission electron microscopy (Sharma et al., 2023). Nevertheless, both SEM and TEM have the drawback of taking time to prepare samples, requiring laborious stages, and posing some danger of artifact creation. Neither approach is appropriate for increased sampling rates and characterization of thousands of individual particles from a single sample. Furthermore, performing statistical analyses for clinical daily routines in which samples must frequently be examined concurrently or within a brief amount of time is problematic (Shi et al., 2023). Newer methods, such as ambient SEM, currently enable us to examine EXOs without requiring extensive preparation. These current approaches remain rather cumbersome for assessing large volume solutions harboring EXOs to evaluate their average quantity and size distribution.

Nanoparticle-tracking analysis (NTA) is another extremely accurate approach for detecting and analyzing EXOs. This strategy makes use of two distinct physical concepts. Initially, when a laser beam is used for radiating particulates, the light dispersed by them is measured (Santos-Coquillat et al., 2022). The second phenomenon is commonly referred to as Brownian motion, which states that the dispersion of particles in a liquid solution is inversely correlated to their sizes. This method is very effective for examining particles with an average diameter of below 100 nm (Cordero et al., 2025).

The dimension and concentration are measured using the ZetaView Brownian and Electrophoresis Motion Video Analysis Microscope. This is a partially computerized desktop nanoparticle monitoring equipment for liquid samples (also known as the particle monitoring analyzer). It comprises a particle detector and a laptop loaded with data processing software. This approach works equally well with complex biological specimens and homogenous inorganic particle suspensions. A laser scattering microscope equipped with a video camera is used to identify particles and track their course (Sausset et al., 2023).

While determining the best method for analyzing separated EXOs is one issue, another is effectively isolating EXOs from various media like blood, urine, or cell cultures. So far, several approaches have been published, including ultracentrifugation, commercial kits (for example, Exoquick), magnetic beads for antigen segregation, and ultrafiltration stages (Capriglione et al., 2022).

Filipe et al. contrasted NTA and DLS to analyze polystyrene beads with established diameters. Diameters of used beads (60, 100, 200, 400, and 1,000 nm) appeared comparable for both approaches; however, larger particles were measured somewhat larger using DLS (e.g., 1,056 nm [DLS] against 989 nm [NTA]. A 100/400 nm bead mixture was spiked with 1,000 nm beads to assess the impact of a tiny amount of bigger particles. DLS observed a peak at 750 nm, which appeared to be an average of the three sizes, but NTA was capable of resolving peaks at 106/420 and 997 nm, providing an improved understanding of the combination (Filipe et al., 2010).

Van der Pol and coworkers examined the particle size arrangement of EXOs from urine and polystyrene beads using TEM, flow cytometry, NTA, and resistive pulse sensing (RPS). Polystyrene beads and urinary EXOs were analyzed. Forward scatter (FSC) was linked to the dimension of vesicles utilizing beads of identified dimensions and the Mie theory. Remarkably, each approach produced a varied range of sizes and content for the same vesicle specimen. These disparities were mostly caused by variances in the minimally detected vesicle measurements, which were 70–90 nm for NTA, 70–100 nm for RPS, 150–190 nm for the specialist flow cytometer, and 270–600 nm for ordinary flow cytometry (Van der Pol et al., 2014).

Maas et al. examined EXOs sorting employing three distinct detection approaches: NTA, RPS, and an optically tuned excellent quality FCM. They examined both EXOs and artificial analogues like liposomes of established sizes. They discovered more disparities in quantification for liposomes than for EXOs using all three approaches. When fluorescence-based activation was used, however, RPS and flow cytometry (FCM) produced equivalent EEXOs quantification findings. This was most likely owing to changes in the quantity of fluorophore molecules associated with EXOs vs. liposomes caused by varying sizes or labeling efficiency (Maas et al., 2015).

Additionally, van der Pol et al. discovered that EV measurement by FCM was 15 times lower than NTA and RPS. These disparate results could potentially be attributed to changes in the EXOs purifying procedure, FCM equipment, threshold triggering mechanism, and kind of EXOs evaluated. These findings highlight the significance of standardization and the execution of additional meticulous research like this (Van der Pol et al., 2014).

### 7.3 Cell culture

Appropriate culturing, division, and differentiation methods are critical for successful therapy results. As a result, choosing the best culture procedure and methodologies for MSCs cultivation is critical. Culture medium, cell count, and the environmental factors, including soluble O2 and CO2 levels, pH, and temperature, all have an impact on study outcomes (Clément et al., 2022).

In investigations that extract EXOs from the culture medium, EXOs extracted from body fluids utilized in the media may contaminate the results. To reduce impurities, it is advised to incubate cells for EXOs investigations in serum-free medium (Ahmadian et al., 2024). If this is not achievable, it is critical to separate EXOs supplied to the culturing media for control (Takagi et al., 2021).

EXOs generated from cells cultivated in standard cell culture dishes vs. two-chamber bioreactors had comparable shape, size distribution, and surface indicators. Nevertheless, the resulting quantity of EXOs was more than 100 times higher in bioreactors than in dishes, and the metabolomic data indicated substantial changes (Palviainen et al., 2019). For optimal development, the different kinds of cells demand a distinct medium composition, as well as seeding volume, passage frequency, and medium replenishment. Aside from adjusting sample purity, Patel et al. demonstrated that using varied cell counts may possess a direct influence on exosome cargo and activities (Patel et al., 2017). To ensure the repeatability and consistency of the amounts and payload of separated EXOs, the same cell seeding process must be established and followed. Utilizing a comparable passage of producing cells improves the consistency of exosome quality (Xu et al., 2024; Salehpour et al., 2024).

### 7.4 Storage

The storage of EXOs is an important factor affecting the outcomes of EXOs investigations. Freezing materials at −70 °C prevents crystal formation, reduces cryo-precipitation, and preserves EXOs (Lee et al., 2016). Furthermore, it is recommended that the aliquots be frozen and thawed only once before analysis (Maroto et al., 2017). To acquire trustworthy findings, select the EXOs separation technique based on the cargo to be tested. It is also critical to carry out replications and compensate for numerous comparisons (Willms et al., 2016).

Moreover, repetitive freeze-thaw cycles may reduce the amount of exosome indicators, perhaps due to membrane damage. The short shelf life of liquid treatments filled with EXOs, along with severe freezing requirements, continues to hamper their clinical adoption. As a result, several investigations have turned attention to the production of solid agents, notably freeze-dried powder incorporating EXOs (An et al., 2023).

In addition to the proprietary freeze-drying process, a common strategy in experiments is to gather EXOs utilizing the previously outlined separation method. Following that, a freeze dryer is used to manufacture freeze-dried EXOs in accordance with a widely recognized “freezing step program,” which usually comprises generic procedures including freezing, vacuum, sublimation, and drying (Dehghani P. et al., 2024). This method preserves the proteins and vesicle configuration of EXOs, resulting in consistent biophysical characteristics even when maintained at −20 °C or ambient temperature (Popowski et al., 2022). Scientists have tried adding a variety of lyoprotectants, such as sucrose, trehalose, mannitol, and other analogous compounds, to minimize lyophilization destruction and preserve the quality and dimensions of EXOs. However, the possible effects of freeze-drying methods on biomolecules such as miRNAs need to be further investigated.

### 7.5 Standardization of functional assays

EXOs have much potential as a means of transport for proteins, RNAs, and tiny molecules in medicine because of their native origin and biocompatibility. However, there are still many issues with their bioavailability, systemic stability, and pharmacokinetics. Research indicates that most of the intravenously administered EXOs are quickly removed from circulation by the mononuclear phagocyte system in a matter of minutes, thus reducing their therapeutic range (Honda et al., 2025).

The kind of donor cell, surface ligands, and delivery technique can all have a substantial impact on the biodistribution of EXOs. Techniques, including surface modifications, PEGylation, or fusion with targeted peptides, are being investigated to extend their circulation duration. However, these methods still require thorough safety evaluation and additional improvement. Furthermore, following distribution, EXOs experience physiological alterations that are still unclear and pose serious translational hazards, including morphological reconstruction, cargo disintegration, or fusion with undesired cells (Nakase, 2021).

EXOs’ dual immunomodulatory role adds to the complexity of the issue. In order to optimize the effectiveness of therapy, the competing immune reactions must be carefully handled, which emphasizes the necessity of exact management of the source and composition of EXOs in medicinal preparations (Li et al., 2022e).

Upgraded genetic designs, including inducible knock-in systems and CRISPR/Cas9-mediated lineage tracing, are crucial for comprehending the complexities of exosome synthesis and cargo packing *in vivo* and for overcoming the existing obstacles in exosome exploration. Integrating multi-omics techniques may assist in discovering biochemical signatures that describe distinct exosome subpopulations (Aslan et al., 2024).

Recent developments like barcoded RNA sequencing and single-vesicle analysis tools have greatly improved our capacity to examine exosome polymorphism in previously unheard-of depth. Furthermore, early cancer identification and monitoring have been greatly enhanced by the combination of EXOs RNA characterization and circulating tumor DNA (ctDNA) analysis, which represents a substantial advancement in personalized oncology. Additionally, the use of machine learning and AI-driven data integration holds significant potential for improving the precision of therapeutic prognosis and speeding up the discovery of EXOs biomarkers (Galbiati et al., 2021).

To reach their maximum therapeutic capacity, EXOs-based technologies must overcome a number of significant obstacles. These involve problems with delivery stability, biogenesis, separation techniques, and a better comprehension of their intricate biological function. It will take multidisciplinary creativity to overcome these challenges, but doing so may open the door to turning exosome-based technologies into revolutionary instruments for next-generation diagnostics and individualized therapies (Li et al., 2022a; Zhou Y. et al., 2023; Wang et al., 2022a).

## 8 Clinical translation of exosomes

EXOs increased wound healing in preclinical models and encouraged tissue regeneration by transferring bioactive molecules (del Pozo-Acebo et al., 2021). Their impacts were achieved via regulating inflammation, angiogenesis, cellular division, and matrix production (Chen et al., 2022). EXOs provide an attractive cell-free treatment for tissue engineering. However, obstacles remain in scaling isolation, understanding processes, and applying this technique in human trials (Akhlaghpasand et al., 2024). Addressing these issues will allow for effective clinical implementation of EXOs for personalized medical applications. Establishing therapeutic uses with exosome technology has become a prominent area of research in recent years (Pak et al., 2023). Their application as medicinal carriers has sparked a lot of curiosity and financial investment. To demonstrate this, the total amount of clinical trials employing EXOs has increased sevenfold over the previous 5 years, with targeted illness areas such as cancer, inflammatory conditions, and immunotherapy (Dhodapkar et al., 2025).

Another significant concern is the stability and lifespan of EXOs in bodily fluids. Getting enough EXOs is the biggest obstacle. An initial amount of 10–100 µg of EXOs is needed for this. However, the amount obtained from 1 mL of culture media is often less than 1 μg, which is the issue (Charoenviriyakul et al., 2017; Yamashita et al., 2016). Exosome quality varies as well; when extracted from biological fluids, they are frequently marked by poor performance and impurities (Zheng et al., 2022). The culture media’s volume, composition, cell passage, and viability all have an impact on exosome retrieval. Standardizing procedures and optimizing manufacturing are crucial for obtaining EXOs (Zhu et al., 2021).

Cultures in bioreactors are utilized to generate a significant number of EXOs; brief cultures can yield even ten times larger numbers (Lee et al., 2023). Physical, chemical, and biological stress are among the disorders that affect exosome formation. Another potential source of contamination is the nutrition media (Clément et al., 2022). Although serum-free circumstances put cells under much stress and change the production of EXOs, EXOs obtained from culture media incorporating serum possess few contaminants (Wang et al., 2025).

Exosome usage in regenerative medicine lowers the possibility of undesirable side effects linked to cell transplantation (Tu et al., 2021). Additionally, such substances are therapeutic modulators because they may alter target molecules in recipient cells, such as by reducing inflammation. EXOs can be harmed during separation and purification, which is a drawback of employing them (Emam et al., 2021).

Despite a number of obstacles, the development of EXOs as medication transporters is moving forward quickly (Zhao et al., 2024). The primary challenges for EXOs’ delivery technologies to reach clinical stages are their quick elimination from the bloodstream and their poor targeting capability. Naturally, a number of engineering techniques have been established to produce engineered EXOs with improved efficacy and sensitivity (Choi et al., 2025).

As far as we know, there is no current clinical trial employing exosome-based treatment in the management of female infertility disorders. However, there is a specimen-collection research posted on clinicaltrials.gov assessing the influence of EXOs on clinical effects in advanced ovarian cancer (Dorayappan et al., 2016). In the first phase I clinical trial (NCT01159288), the scientists administered autologous dendritic cells EXOs as a vaccine against metastatic melanoma and demonstrated their safety. Nevertheless, they did not see substantial CD4^+^  or CD8^+^  T cell responses. It is still necessary to investigate the mechanistic processes behind vaccination antigen dispersion (Escudier et al., 2005). Additionally, the first-in-human clinical study utilizing allogeneic, platelet-derived EXOs as a possible treatment for delayed wound healing was carried out by Johnson et al. In this study, we show that platelet EXOs (pEXOs) of clinical grade may be effectively separated from active platelets using Ligand-based Exosome Affinity Purification (LEAP) chromatography while maintaining the parent cell’s capacity for regeneration. LEAP-isolated pEXOs carry vital proteins involved in wound healing processes, such as insulin growth factor (IGF) and transforming growth factor beta (TGF-ß), and exhibit the anticipated biophysical characteristics of EXOs populations. According to *in vitro* research, pEXOs enhance the angiogenic potential of dermal endothelial cells and promote the migration and proliferation of dermal fibroblasts, indicating their capacity to repair wounds. The ERK and Akt signaling pathways in recipient cells are activated by pEXOs therapy. They showed that injections of LEAP-purified pEXOs demonstrated acceptable safety in their phase I clinical trial of healthy volunteer adults, which was primarily conducted to evaluate safety with regard to wound healing (Plexoval II study, ACTRN12620000944932). Following administration of a single dosage of pEXOs, all wounds closed quickly and entirely. The results of this investigation demonstrate that pEXOs produced using the LEAP process can be safely administered to humans as an alternative therapy for wound healing, and they call for additional research in clinical trials created specifically to evaluate the therapeutic effectiveness in patients with retarded or interrupted wound healing (Johnson et al., 2023).

The clinical applications of EXOs are still in their early stages; future research will help to identify cost-effective and time-efficient approaches for massive exosome synthesis. In fact, EXOs can transport relevant medicinal products used for disease management (Chandran et al., 2025). It is essential to determine suitable approaches for additional personalizing EXOs as pharmaceutical transporters with a substantial carrying capability, outstanding selectivity, biocompatibility, and minimal immunogenicity (Huo et al., 2025). In addition, the separation, categorization, and purification of EXOs must be standardized to ensure that EXOs can be used clinically (Cordero et al., 2025). Without a question, EXOs are an exciting development in the realm of nanomedicine and may hold the key to solving a number of current medical problems (Chen Z. et al., 2024). Despite encouraging first findings, few studies demonstrate the superiority of EXOs in delivering FDA-approved nanomedicine (such as liposomes); hence, more research on EXOs as therapeutic agents and pharmaceutical carriers is unavoidable in this setting.

## 9 Future research directions

Reproductive health is intimately linked to individual standards of living. Women are very interested in understanding the mechanics of reproductive aging and how to halt this process (Liu et al., 2025). Exosome therapy has emerged as a potential star in gynecological research, but there are still several practical issues that impede its clinical implementation (Park et al., 2024).

To enhance the clinical use of EXOs, collaboration across disciplines and fields is required, ranging from fundamental to clinical, medicine to engineering (Jin et al., 2023). EXOs play a crucial role in several biological processes, and their significance in reproductive system problems in females is becoming more widely recognized (Atia et al., 2025; Zhao et al., 2022). EXOs, as a key regulator of interaction between cells, have a significant influence on the mitigation of infertility (Freger et al., 2021; Lin et al., 2024).

Infertility studies regarding exosome roles, underlying processes, and therapeutic capacity are still in their early stages, and many concerns remain unanswered. Given the increasing technological breakthroughs, there is discussion regarding the possible application of exosome-based treatments for infertility (Wang et al., 2024d).

While stem cell-based treatments have proved extremely effective in treating tissue regeneration and reproductive system problems, they are also subject to a number of limitations, including immunogenicity, undesired differentiations, and ethical concerns (Xie et al., 2024).

EXOs have the same functions as MSCs, but they possess the added benefits of focused administration, minimal antigenicity and immunological rejection, and great repair potential (Cai et al., 2022; Wang et al., 2024d; Yang et al., 2020b). As previously stated, EXOs have distinctive characteristics and an outstanding therapeutic effectiveness, making them an intriguing therapy option for infertile women (Park et al., 2024).

Although the use of EXOs is becoming more widespread, achieving optimal therapeutic results remains difficult. EXOs undoubtedly play an important part in female fertility. The high therapeutic capacity of EXOs in female reproductive disorders has paved the way for further research (Hadidi et al., 2023). In accordance with the presented findings, using these nano-therapeutics as a cell-free method can overcome several technical issues with cell-based treatments (Abdelnaby et al., 2024).

Currently, integrating EXOs with designed polymers has proven to be highly beneficial in improving exosome selectivity and reliability. Liang et al., for example, used EXOs produced from decidual stromal cells (DSCs) in sodium alginate hydrogel as a new therapeutic method for promoting endometrial rejuvenation and restoring fertility. The researchers discovered that injecting DSCs-derived EXOs (DSCs-EXOs)/SAH into the uterine cavity could stimulate uterine angiogenesis, trigger mesenchymal-to-epithelial transformation (MET), encourage collagen deposition, favor endometrial regeneration, increase endometrial responsiveness, and give rise to fertility restoration (Liang et al., 2024).

EXOs are at a turning point in therapeutic applications. Considering their recent identification as crucial participants in physiology and disease, the utilization of these tiny vesicles in therapeutic applications is quickly growing (Navarro et al., 2025; Zhang et al., 2025; Vaiciuleviciute et al., 2025).

The major therapeutic uses in cancer and inflammation take advantage of the exosome’s involvement in immune system modulation and its utilization as a vehicle for targeted medication delivery. Numerous scholarly papers, in addition to extremely valuable investments in pharmaceutical research focused on EXOs, reveal widespread and cross-sector interest in these biotechnological advances (Wang et al., 2024a; Wang et al., 2024d; Taravat et al., 2024; Li et al., 2024b).

Several techniques for increasing the therapeutic effectiveness of EXOs are being researched. The regulatory environment is improving to enable safe and effective clinical trials. Enhanced scaling-up solutions are achieved to address the limits associated with production and characterization procedures. Thus, while still in its earliest stages, the exosome area is rapidly maturing for the greater good of patients (Liang et al., 2024).

Exosome-containing scaffolds have proven to be far more successful in mending injured tissues than scaffolding or EXOs alone. The immediate disposal of these scaffolds will likely be employed at the bedside for better and quicker tissue restoration. Although there are a number of techniques for isolating and characterizing EXOs, some can be too costly or time-consuming for researchers (Wu et al., 2024). Therefore, it is necessary to overcome the current problems in order to expand the practical usage of EXOs. Several avenues of research might be useful in expanding the comprehension of exosome-based medicines and their prospective uses, including the following: 1 Additional research is needed to standardize and validate exosome-based products. The regulatory environment for exosome-based therapeutics requires additional development and clarification. 2 Exosome-mediated delivery requires a thorough understanding of its biological mechanics. Approaches for monitoring the fate of EXOs require further research. EXOs should be investigated in conjunction with other medicinal agents. The creation of tailored therapeutics based on patient-derived EXOs should be researched.

Besides these fields of inquiry, there are numerous more possible uses of EXOs that need further examination, as the follows: 1 EXOs have the potential to cure several disorders in regenerative medicine. 2 Exosome-based gene therapy has the potential to address several genetic problems. 3 EXOs in vaccine development have the potential to treat several ailments, notably infectious disorders and cancer. 4 EXOs in diagnostics might be utilized to diagnose a variety of disorders.

In general, our comprehension of the biological functions of EXOs is inadequate, and further research is required. Nonetheless, the utilization of EXOs in complicated clinical settings in the near future is not surprising.

## 10 Concluding remarks

Reproductive wellness is closely related to individual standards of living. Women are very interested in understanding the mechanics of fertility disorders and how to tackle their issues. Exosome therapy offers several theranostics potentials in experimental studies, but there are still several practical issues that impede their clinical translation. To enhance the clinical administration of EXOs, collaboration across disciplines and fields is required, ranging from fundamental to clinical, medicine to engineering. As a result, in this article, we completely explain the roles and fundamental processes of exosome treatment in treating female infertility issues.

Animal investigations have shown that exosome treatment can fight apoptosis and promote regeneration which consequently results in counteracting of reproductive aging. However, clear clinical proof is now absent, and further basic study is needed to investigate their bioactivity. Given the similar clinical hallmarks of inflammation and fibrosis, exosome treatment established for heart disease may be useful to other disciplines, including reproductive aging. Furthermore, there are significant uncertainties associated with the exosome manufacturing process.

We present a comprehensive description of the complete process, from exosome separation, filtration, and sourcing to agent preservation, applications, and customization. Quality control procedures for EXOs and their metabolites are thoroughly reviewed. This detailed explanation seeks to provide prospective investigators with a clear grasp of the limits of current methodologies, allowing them to make educated decisions throughout the exosome research process. Furthermore, we discuss alternative remedies to present problems. The combination of many modern innovations with EXOs have shown limitless possibilities for progression, and it is set to increase their potentials in personalized medicine.

While the use of EXOs is becoming more and more popular, there are still several obstacles in the way of achieving satisfactory therapeutic results. EXOs undoubtedly have a big impact on female fertility, egg implantation, and embryo development. Further research has been made possible by the outstanding therapeutic effectiveness of EXOs in conditions associated with female reproduction. The published results indicate that some technical issues with cell-based treatments can be resolved by using these nanotherapeutics as a cell-free method. At the moment, exosome durability and targeting capacity have been greatly improved by integrating them with synthetic polymer-based biomaterials. More research is required since there is currently a dearth of information on the pharmacological specifics of EXOs and exosome-loaded biomaterials. However, it is not at all surprising that EXOs will be used in complicated clinical diseases in the years to come.

## Glossary

ADSC-EXOs: Adipose stem cell-derived exosomes
AEC: Amniotic epithelial cells
AFC: Antral follicle count
AFSC-EXOs: Amniotic fluid stem cell-derived exosomes
AITC: Allyl isothiocyanate
ALOX15m: Arachidonate 15-lipoxygenase
AMH: Anti-müllerian hormone
AMH: Anti-müllerian hormone
AMSCs: Amniotic mesenchymal stem cells
AS: Asherman syndrome
ATPS: Aqueous two-phase system
BBB: Blood-brain barrier
BMI: Body mass index
BMMSCs: Bone marrow mesenchymal stem cells
CARPA: Complement activation-related pseudoallergy
C-BMSCs: Canine bone marrow mesenchymal stem cells
CCN2: Cellular communication network-2
CCs: Cumulus cells
CD: Cluster of differentiation
CDK4: Cyclin-dependent kinase 4
COX: Cyclo-oxygenase
CS: Collagen scaffold
CTF1: Cardiotrophin-1
CTX: Cyclophosphamide
DALYs: Disability-adjusted life years
DBCO: Dibenzocyclooctyne
DDSs: Drug delivery systems
DEP: Dielectrophoretic
DEX: Dextran
DLEU1: Deleted in lymphocytic leukemia1
DSCs: Decidual stromal cells
E2: Estradiol
EC: Endometrial cancer
ECM: Extracellular matrix
EE: Ectopic endometrium
EECs: Ectopic endometrium cells
EESCs: Ectopic endometrial stromal cells
EMS: Endometriosis
ENK-EXOs: Exosomes derived from expanded natural killer cells
ESCs: Endometrial stem cells
EUE: Eutopic endometrium
EVs: Extracellular vesicles
EXOs: Exosomes
EXOs-Anthos: EXOs anthocyanidins
EXOs-HP: Mesenchymal stem cell-derived exosomes encapsulated in heparin-poloxamer hydrogel
FACS: Fluorescence activated cell sorting
FCM: Flow cytometry
FDA: Food and drug agency
TFF: Tangential flow filtration
FF: Follicular fluid
FGA: Fibrinogen alpha chain
FGG: Fibrinogen gamma chain
FSH: Follicle-stimulating hormone
GALA: Glu-Ala-Leu-Ala
GPX1: Glutathione peroxidase 1
GSN: Gelsolin
HEnSCs: human endometrial stromal cells
HENPs: Hybrid exosomes nanoparticles;
HIF-1α: Hypoxia-inducible factor1-alpha
HO-1: Heme oxygenase-1
HUCMSCs: Human umbilical cord mesenchymal stem cells
HUVECs: Human umbilical vein endothelial cells
I/R: Ischemia-reperfusion
IDEM: Immune derived exosome mimetics
IGF: Insulin growth factor
IL: Interleukin
IUA: Intra uterine adhesions
LATS1: Large tumor suppressor kinase 1
LEAP: Ligand-based exosome affinity purification
LH: Luteinizing hormone
LIF: Leukemia inhibitory factor
LOXL2: Lysyl oxidase homolog 2
LPB: Binding protein
MDE: Mammalian derived exosomes
MenSCs: Menstrual blood stromal cells
MEST: Mesoderm-specific transcript
MHC: Major histocompatibility complex
MiRNAs: Micro ribonucleicacids
MMP2: Matrix metalloproteinase 2
MnSOD: Manganese-containing superoxide dismutase
MSCs: Mesenchymal stem cells
NF-kB: Nuclear factor kappa B
NK: Natural killer
NP: Nucleus pulposus
Nrf2: Nuclear factor erythroid 2-related factor 2
NTA: Nanoparticle-tracking analysis
OCSCs: Ovarian cancer stem cells
OGCs: Ovarian granulosa cells
AITC: Allyl isothiocyanate
PAC: Paclitaxel
PBMCs: Peripheral blood mononuclear cells
PCNA: Proliferating cell nuclear antigen
PCOS: Polycystic ovary syndrome
pEXOs: Platelet exosomes
PD: Parkinson’s disease
PEG: Poly ethylene Glycol
PEN1: Penetration
PI3K: Phosphoinositide-3-kinase
PI3Ks: Phosphoinositide 3-kinases
PlGF: Placental growth factor
pMφ: Peritoneal macrophages
PODXL: Podocalyxin-like
POI: Primary ovarian insufficiency
PPARγ: Peroxisome proliferator activated receptor gamma
PRP: Platelet rich plasma
PTEN: Phosphatase and tensin homolog deleted on chromosome 10
PTr2: Pig trophoblast cells
SASP: Senescence-connected secretory phenotype
SDF-1α: Stromal cell derived factor-1 alpha
SEC: Size-exclusion chromatography
SEM: Scanning electron microscopy
sFlt-1: Fms-like tyrosine kinase-1
SOD1: Superoxide dismutase 1
STAT3: Signal transduction and activation of transcription 3
T: Testosterone
TAMs: Tumor associated macrophages
TEM: Transmission electron microscopy
TGF: Transforming growth factor
THP: Tamm horsfall protein
TP: Triptolide
TrxR2: Thioredoxin reductase
UC-MSCs: Umbilical cord derived mesenchymal stem cells
VCAN: Versican
VCD: Vinylcyclohexene diepoxide
VEGF: Vascular endothelial growth factor

## References

[B1] Aare M. Bagde A. Nathani A. Rishi A. K. Singh M. (2024). Enhanced oral bioavailability and *in vitro* evaluation of cannabidiol camel milk-derived exosome formulation in resistant MDA-MB-231 and MDA-MB-468 breast cancer cells. Int. J. Pharm. 663, 124375. 10.1016/j.ijpharm.2024.124375 38914353 PMC12326876

[B2] Abbas M. Kushwaha V. S. Srivastava K. Banerjee M. (2022). Understanding role of DNA repair and cytochrome p-450 gene polymorphisms in cervical cancer patient treated with concomitant chemoradiation. Br. J. Biomed. Sci. 79, 10120. 10.3389/bjbs.2021.10120 35996502 PMC8915685

[B3] Abdelnaby E. A. Abdallah A. N. Anwar I. M. El‐Tookhy O. S. Shamaa A. A. (2024). The therapeutic effect of stem cell‐derived exosomes in the treatment of chronic endometritis as assessed by histopathological, doppler and hormonal expression in Arabian mares. Equine Veterinary Educ. 36, 347–356. 10.1111/eve.13888

[B4] Abla K. K. Mehanna M. M. (2022). Freeze-drying: a flourishing strategy to fabricate stable pharmaceutical and biological products. Int. J. Pharm. 628, 122233. 10.1016/j.ijpharm.2022.122233 36183914

[B5] Abusamra A. J. Zhong Z. Zheng X. Li M. Ichim T. E. Chin J. L. (2005). Tumor exosomes expressing fas ligand mediate CD8+ T-cell apoptosis. Blood Cells, Mol. Dis. 35, 169–173. 10.1016/j.bcmd.2005.07.001 16081306

[B6] Ahmadian S. Jafari N. Tamadon A. ghaffarzadeh A. Rahbarghazi R. Mahdipour M. (2024). Different storage and freezing protocols for extracellular vesicles: a systematic review. a Syst. Rev. 15, 453. 10.1186/s13287-024-04005-7 39593194 PMC11600612

[B7] Ahmadieh-Yazdi A. Karimi M. Afkhami E. Hajizadeh-Tafti F. Kuchakzadeh F. Yang P. (2024). Unveiling therapeutic potential: adipose tissue-derived mesenchymal stem cells and their exosomes in the management of diabetes mellitus, wound healing, and chronic ulcers. Biochem. Pharmacol. 226, 116399. 10.1016/j.bcp.2024.116399 38944396

[B8] Ahmed W. Mushtaq A. Ali S. Khan N. Liang Y. Duan L. (2024). Engineering approaches for exosome cargo loading and targeted delivery: biological *versus* chemical perspectives. ACS Biomaterials Sci. and Eng. 10, 5960–5976. 10.1021/acsbiomaterials.4c00856 38940421

[B9] Akhlaghpasand M. Tavanaei R. Hosseinpoor M. Yazdani K. O. Soleimani A. Zoshk M. Y. (2024). Safety and potential effects of intrathecal injection of allogeneic human umbilical cord mesenchymal stem cell-derived exosomes in complete subacute spinal cord injury: a first-in-human, single-arm, open-label, phase I clinical trial. Stem Cell Res. and Ther. 15, 264. 10.1186/s13287-024-03868-0 39183334 PMC11346059

[B10] Akinduro O. Kumar S. Chen Y. Thomas B. Hassan Q. Sims B. (2025). Human breast milk-derived exosomes attenuate lipopolysaccharide-induced activation in microglia. J. Neuroinflammation 22, 41. 10.1186/s12974-025-03345-2 39955566 PMC11830176

[B11] Alesi S. Ee C. Moran L. J. Rao V. Mousa A. (2022). Nutritional supplements and complementary therapies in polycystic ovary syndrome. Adv. Nutr. 13, 1243–1266. 10.1093/advances/nmab141 34970669 PMC9340985

[B12] Alesi S. Teede H. Enticott J. De Silva K. Mousa A. (2024). Blood-based inflammatory markers in female infertility: evidence from Mendelian randomization analysis. 10.1016/j.xfss.2024.11.001 39542215

[B14] An S. Anwar K. Ashraf M. Lee H. Jung R. Koganti R. (2023). Wound-healing effects of mesenchymal stromal cell secretome in the cornea and the role of exosomes. Pharmaceutics 15, 1486. 10.3390/pharmaceutics15051486 37242728 PMC10221647

[B15] Andreescu M. (2023). The impact of the use of immunosuppressive treatment after an embryo transfer in increasing the rate of live birth. Front. Med. 10, 1167876. 10.3389/fmed.2023.1167876 37441690 PMC10333755

[B16] Andronico F. Battaglia R. Ragusa M. Barbagallo D. Purrello M. Di Pietro C. (2019). Extracellular vesicles in human oogenesis and implantation. Int. J. Mol. Sci. 20, 2162. 10.3390/ijms20092162 31052401 PMC6539954

[B17] Aqil F. Jeyabalan J. Agrawal A. K. Kyakulaga A.-H. Munagala R. Parker L. (2017). Exosomal delivery of berry anthocyanidins for the management of ovarian cancer. Food Funct. 8, 4100–4107. 10.1039/c7fo00882a 28991298

[B18] Ara I. Maqbool M. Zehravi M. (2022). Psychic consequences of infertility on couples: a short commentary. arXiv 3, 114–119. 10.1515/openhe-2022-0022

[B19] ArteK. S.ChenM.PatilC. D.HuangY.QuL.ZhouQ. (2025). Recent advances in drying and development of solid formulations for stable mRNA and siRNA lipid nanoparticles. J. Pharm. Sci. 114, 805–815. 10.1016/j.xphs.2024.12.013 39694272

[B20] Aslan C. Zolbanin N. M. Faraji F. Jafari R. (2024). Exosomes for CRISPR-Cas9 delivery: the cutting edge in genome editing. Mol. Biotechnol. 66, 3092–3116. 10.1007/s12033-023-00932-7 38012525

[B21] Atia G. A. Abdal Dayem A. Taher E. S. Alghonemy W. Y. Cho S.-G. Aldarmahi A. A. (2025). Urine-derived stem cells: a sustainable resource for advancing personalized medicine and dental regeneration. Front. Bioeng. Biotechnol. 13, 1571066. 10.3389/fbioe.2025.1571066 40357329 PMC12066649

[B22] Bai K. Li X. Zhong J. Ng E. H. Yeung W. S. Lee C.-L. (2021). Placenta-derived exosomes as a modulator in maternal immune tolerance during pregnancy. Front. Immunol. 12, 671093. 10.3389/fimmu.2021.671093 34046039 PMC8144714

[B23] Bai L. Gong J. Guo Y. Li Y. Huang H. liu X. (2022). Construction of a ceRNA network in polycystic ovary syndrome (PCOS) driven by exosomal lncRNA. Front. Genet. 13, 979924. 10.3389/fgene.2022.979924 36406137 PMC9672461

[B24] Bai K. Li J. Lin L. Zhang Q. Zhong J. Liu X. (2023). Placenta exosomal miRNA-30d-5p facilitates decidual macrophage polarization by targeting HDAC9. J. Leukoc. Biol. 113, 434–444. 10.1093/jleuko/qiad022 36821782

[B25] Bala R. Singh V. Rajender S. Singh K. (2021). Environment, lifestyle, and female infertility. Reprod. Sci. 28, 617–638. 10.1007/s43032-020-00279-3 32748224

[B26] Balaj L. Atai N. A. Chen W. Mu D. Tannous B. A. Breakefield X. O. (2015). Heparin affinity purification of extracellular vesicles. Sci. Rep. 5, 10266. 10.1038/srep10266 25988257 PMC4437317

[B27] Barreiro K. Dwivedi O. P. Leparc G. Rolser M. Delic D. Forsblom C. (2020). Comparison of urinary extracellular vesicle isolation methods for transcriptomic biomarker research in diabetic kidney disease. J. Extracell. Vesicles 10, e12038. 10.1002/jev2.12038 33437407 PMC7789228

[B28] Berdiaki A. Vergadi E. Makrygiannakis F. Vrekoussis T. Makrigiannakis A. (2024). Title: repeated implantation failure is associated with increased Th17/Treg cell ratio, during the secretory phase of the human endometrium. J. Reprod. Immunol. 161, 104170. 10.1016/j.jri.2023.104170 38011769

[B29] Berkel C. Cacan E. (2021). Transcriptomic analysis reveals tumor stage-or grade-dependent expression of miRNAs in serous ovarian cancer. Hum. Cell 34, 862–877. 10.1007/s13577-021-00486-3 33576947

[B30] Bernardi S. Farina M. (2021). Exosomes and extracellular vesicles in myeloid neoplasia: the multiple and complex roles played by these “magic bullets”. Biology 10, 105. 10.3390/biology10020105 33540594 PMC7912829

[B31] Bethi C. M. S. Kumar M. N. Kalarikkal S. P. Narayanan J. Sundaram G. M. (2025). Fenugreek-derived exosome-like nanovesicles containing bioavailable phytoferritin for the management of iron deficiency anemia. Food Chem. 490, 145088. 10.1016/j.foodchem.2025.145088 40499430

[B32] Bhadra M. Sachan M. (2024). An overview of challenges associated with exosomal miRNA isolation toward liquid biopsy-based ovarian cancer detection. Heliyon 10, e30328. 10.1016/j.heliyon.2024.e30328 38707279 PMC11068823

[B33] Bhardwaj J. K. Panchal H. Saraf P. (2021). Ameliorating effects of natural antioxidant compounds on female infertility: a review. a Rev. 28, 1227–1256. 10.1007/s43032-020-00312-5 32935256

[B34] Bhat A. Yadav J. Thakur K. Aggarwal N. Chhokar A. Tripathi T. (2022). Transcriptome analysis of cervical cancer exosomes and detection of HPVE6* I transcripts in exosomal RNA. Bmc Cancer 22, 164. 10.1186/s12885-022-09262-4 35148692 PMC8840784

[B35] Bhuia M. S. Chowdhury R. Sonia F. A. Kamli H. Shaikh A. El‐Nashar H. A. S. (2023). Anticancer potential of the plant‐derived saponin gracillin: a comprehensive review of mechanistic approaches. Chem. and Biodivers. 20, e202300847. 10.1002/cbdv.202300847 37547969

[B36] Bian Y. Chang X. Hu X. Li B. Song Y. Hu Z. (2024). Exosomal CTHRC1 from cancer-associated fibroblasts facilitates endometrial cancer progression *via* ITGB3/FAK signaling pathway. Heliyon 10, e35727. 10.1016/j.heliyon.2024.e35727 39229506 PMC11369458

[B37] Björk E. Israelsson P. Nagaev I. Nagaeva O. Lundin E. Ottander U. (2024). Endometriotic tissue–derived exosomes downregulate NKG2D-mediated cytotoxicity and promote apoptosis: mechanisms for survival of ectopic endometrial tissue in endometriosis. J. Immunol. 213, 567–576. 10.4049/jimmunol.2300781 38984872 PMC11335327

[B38] Bowers E. C. Hassanin A. A. Ramos K. S. (2020). *In vitro* models of exosome biology and toxicology: new frontiers in biomedical research. Toxicol. Vitro 64, 104462. 10.1016/j.tiv.2019.02.016 31628015 PMC6814514

[B39] Cabasag C. J. Fagan P. J. Ferlay J. Vignat J. Laversanne M. Liu L. (2022). Ovarian cancer today and tomorrow: a global assessment by world region and human development index using GLOBOCAN 2020. Int. J. Cancer 151, 1535–1541. 10.1002/ijc.34002 35322413

[B40] Cai J.-H. Sun Y.-T. Bao S. (2022). HucMSCs-exosomes containing miR-21 promoted estrogen production in ovarian granulosa cells *via* LATS1-mediated phosphorylation of LOXL2 and YAP. General Comp. Endocrinol. 321, 114015. 10.1016/j.ygcen.2022.114015 35271888

[B41] Cao M. Zhao Y. Chen T. Zhao Z. Zhang B. Yuan C. (2022). Adipose mesenchymal stem cell-derived exosomal microRNAs ameliorate polycystic ovary syndrome by protecting against metabolic disturbances, Biomaterials 288, 121739. 10.1016/j.biomaterials.2022.121739 35987860

[B42] Cao Y. Qin Y. Cheng Q. Zhong J. Han B. Li Y. (2025). Bifunctional nanomaterial enabled high-specific isolation of urinary exosomes for cervical cancer metabolomics analysis and biomarker discovery. Talanta 285, 127280. 10.1016/j.talanta.2024.127280 39613490

[B43] Capriglione F. Verrienti A. Celano M. Maggisano V. sponziello M. Pecce V. (2022). Analysis of serum microRNA in exosomal vehicles of papillary thyroid cancer. Endocrine 75, 185–193. 10.1007/s12020-021-02847-2 34378123

[B44] Castle P. E. Einstein M. H. Sahasrabuddhe V. V. (2021). Cervical cancer prevention and control in women living with human immunodeficiency virus. CA a cancer J. Clin. 71, 505–526. 10.3322/caac.21696 34499351 PMC10054840

[B45] Cervelló I. Santamaría X. Miyazaki K. Maruyama T. Simón C. (2015). Cell therapy and tissue engineering from and toward the uterus. Seminars reproductive Med. 33, 366–372. 10.1055/s-0035-1559581 26285168

[B46] Chandran N. S. Bhupendrabhai M. N. Tan T. T. Zhang B. lim S. K. Choo A. B. H. (2025). A phase 1, open-label study to determine safety and tolerability of the topical application of mesenchymal stem/stromal cell (MSC) exosome ointment to treat psoriasis in healthy volunteers. Cytotherapy 27, 633–641. 10.1016/j.jcyt.2025.01.007 39918488

[B47] Chang X. He Q. Wei M. Jia L. Wei Y. Bian Y. (2023). Human umbilical cord mesenchymal stem cell derived exosomes (HUCMSC-exos) recovery soluble fms-like tyrosine kinase-1 (sFlt-1)-induced endothelial dysfunction in preeclampsia. Eur. J. Med. Res. 28, 277. 10.1186/s40001-023-01182-8 37559150 PMC10413730

[B48] Chapman R. Chapman K. (1996). The value of two stage laser treatment for severe Asherman's syndrome. BJOG An Int. J. Obstetrics and Gynaecol. 103, 1256–1258. 10.1111/j.1471-0528.1996.tb09641.x 8968248

[B49] Charoenviriyakul C. Takahashi Y. Morishita M. Matsumoto A. Nishikawa M. Takakura Y. (2017). Cell type-specific and common characteristics of exosomes derived from mouse cell lines: yield, physicochemical properties, and pharmacokinetics. Eur. J. Pharm. Sci. 96, 316–322. 10.1016/j.ejps.2016.10.009 27720897

[B50] Chen C. Skog J. Hsu C.-H. Lessard R. T. Balaj L. Wurdinger T. (2010). Microfluidic isolation and transcriptome analysis of serum microvesicles. Lab a Chip 10, 505–511. 10.1039/b916199f 20126692 PMC3136803

[B51] Chen X. Zhou Y. Yu J. (2019). Exosome-like nanoparticles from ginger rhizomes inhibited NLRP3 inflammasome activation. Mol. Pharm. 16, 2690–2699. 10.1021/acs.molpharmaceut.9b00246 31038962

[B52] Chen Z. Ling K. Zhu Y. Deng L. Li Y. Liang Z. (2020). Rucaparib antagonize multidrug resistance in cervical cancer cells through blocking the function of ABC transporters. Gene 759, 145000. 10.1016/j.gene.2020.145000 32717310

[B53] Chen F. Zou L. Dai Y. Sun J. Chen C. Zhang Y. (2021). Prognostic plasma exosomal microRNA biomarkers in patients with substance use disorders presenting comorbid with anxiety and depression. Sci. Rep. 11, 6271. 10.1038/s41598-021-84501-5 33737514 PMC7973758

[B54] Chen Q. Che C. Liu J. Gong Z. Si M. Yang S. (2022). Construction of an exosome-functionalized graphene oxide based composite bionic smart drug delivery system and its anticancer activity. Nanotechnology 33, 175101. 10.1088/1361-6528/ac49bf 35008083

[B55] Chen S. Ma Y. Qiu X. Liu M. Zhang P. Wei C. (2023). MicroRNA-122-5p alleviates endometrial fibrosis *via* inhibiting the TGF-β/SMAD pathway in Asherman's syndrome. Reprod. Biomed. Online 47, 103253. 10.1016/j.rbmo.2023.06.008 37677924

[B56] Chen T.-Y. Huang T.-Y. Chung Y.-Y. Lin W.-C. Lin H.-Y. Chiu H.-C. (2024a). Exosomes derived from Polygonum multiflorum-treated human dental pulp stem cells (hDPSCs): new approach in regenerative medicine. J. Drug Deliv. Sci. Technol. 99, 105941. 10.1016/j.jddst.2024.105941

[B57] Chen Z. Xiong M. Tian J. Song D. Duan S. Zhang L. (2024b). Encapsulation and assessment of therapeutic cargo in engineered exosomes: a systematic review. J. Nanobiotechnology 22, 18. 10.1186/s12951-023-02259-6 38172932 PMC10765779

[B58] Chen S. Lin X. Liu L. Fu J. Lu X. Niu C. (2025). Human umbilical cord mesenchymal stem cell-derived exosomes repair raloxifene-induced damage in endometrial stromal cells *via* autophagy activation. Reprod. Toxicol. 137, 108997. 10.1016/j.reprotox.2025.108997 40652993

[B59] Cheng Q. Dai Z. Smbatyan G. Epstein A. L. Lenz H.-J. Zhang Y. (2022). Eliciting anti-cancer immunity by genetically engineered multifunctional exosomes. Mol. Ther. 30, 3066–3077. 10.1016/j.ymthe.2022.06.013 35746867 PMC9481992

[B60] Cheng B. Luo T. Wu Y. Hu J. Yang C. Wu J. (2025). Urinary exosomal FAM153C-RPL19 chimeric RNA as a diagnostic and prognostic biomarker for prostate cancer in Chinese patients. Cancer Lett. 631, 217938. 10.1016/j.canlet.2025.217938 40684841

[B61] Chernyshev V. S. Rachamadugu R. Tseng Y. H. Belnap D. M. Jia Y. Branch K. J. (2015). Size and shape characterization of hydrated and desiccated exosomes. Anal. Bioanal. Chem. 407, 3285–3301. 10.1007/s00216-015-8535-3 25821114

[B62] Chiarello D. I. Salsoso R. Toledo F. Mate A. Vázquez C. M. Sobrevia L. (2018). Foetoplacental communication *via* extracellular vesicles in normal pregnancy and preeclampsia. Mol. Asp. Med. 60, 69–80. 10.1016/j.mam.2017.12.002 29222068

[B63] Cho K. R. Shih I.-M. (2009). Ovarian cancer. Annu. Rev. pathology Mech. Dis. 4, 287–313. 10.1146/annurev.pathol.4.110807.092246 18842102 PMC2679364

[B64] Cho S. Jo W. Heo Y. Kang J. Y. Kwak R. Park J. (2016). Isolation of extracellular vesicle from blood plasma using electrophoretic migration through porous membrane. Sensors Actuators B Chem. 233, 289–297. 10.1016/j.snb.2016.04.091

[B65] Choi J. Y. Park S. Shim J. S. Park H. J. Kuh S. U. Jeong Y. (2025). Explainable artificial intelligence-driven prostate cancer screening using exosomal multi-marker based dual-gate FET biosensor. Biosens. Bioelectron. 267, 116773. 10.1016/j.bios.2024.116773 39277920

[B66] Chu Y. Chen W. Peng W. Liu Y. Xu L. Zuo J. (2020). Amnion-derived mesenchymal stem cell exosomes-mediated autophagy promotes the survival of trophoblasts under hypoxia through mTOR pathway by the downregulation of EZH2. Front. cell Dev. Biol. 8, 545852. 10.3389/fcell.2020.545852 33304896 PMC7693549

[B67] Chu M. Wang H. Bian L. Huang J. Wu D. Zhang R. (2022). Nebulization therapy with umbilical cord mesenchymal stem cell-derived exosomes for COVID-19 pneumonia. Stem cell Rev. Rep. 18, 2152–2163. 10.1007/s12015-022-10398-w 35665467 PMC9166932

[B68] Ciftci E. Bozbeyoglu N. Gursel I. Korkusuz F. Bakan Misirlioglu F. Korkusuz P. (2023). Comparative analysis of magnetically activated cell sorting and ultracentrifugation methods for exosome isolation. PloS one 18, e0282238. 10.1371/journal.pone.0282238 36854030 PMC9974127

[B69] Clayton A. Court J. Navabi H. Adams M. Mason M. D. Hobot J. A. (2001). Analysis of antigen presenting cell derived exosomes, based on immuno-magnetic isolation and flow cytometry. J. Immunol. methods 247, 163–174. 10.1016/s0022-1759(00)00321-5 11150547

[B70] Clément V. Roy V. Paré B. Goulet C. R. Deschênes L. T. Berthod F. (2022). Tridimensional cell culture of dermal fibroblasts promotes exosome-mediated secretion of extracellular matrix proteins. Sci. Rep. 12, 19786. 10.1038/s41598-022-23433-0 36396670 PMC9672399

[B71] Convery N. Gadegaard N. (2019). 30 years of microfluidics. Micro Nano Eng. 2, 76–91. 10.1016/j.mne.2019.01.003

[B72] Cordero L. Domingo J. C. Mengual E. S.-V. Pinto H. (2025). Autologous platelet-rich plasma Exosome quantification after two thermo-photobiomodulation protocols with different fluences. J. Photochem. Photobiol. 29, 100267. 10.1016/j.jpap.2025.100267

[B73] Cui X.-S. Sun S.-C. Kang Y.-K. Kim N.-H. (2013). Involvement of microRNA-335-5p in cytoskeleton dynamics in mouse oocytes. Reproduction, Fertil. Dev. 25, 691–699. 10.1071/RD12138 22950940

[B74] Cui J. Chen X. Lin S. Li L. Fan J. Hou H. (2020). MiR-101-containing extracellular vesicles bind to BRD4 and enhance proliferation and migration of trophoblasts in preeclampsia. Stem Cell Res. and Ther. 11, 231. 10.1186/s13287-020-01720-9 32527308 PMC7291671

[B75] Dabi Y. Ebanga L. Favier A. Kolanska K. Puchar A. Jayot A. (2024). Discoid excision for colorectal endometriosis associated infertility: a balance between fertility outcomes and complication rates. J. Gynecol. Obstet. Hum. Reprod. 53, 102723. 10.1016/j.jogoh.2024.102723 38211693

[B76] Dalmizrak A. Dalmizrak O. (2022). Mesenchymal stem cell-derived exosomes as new tools for delivery of miRNAs in the treatment of cancer. Front. Bioeng. Biotechnol. 10, 956563. 10.3389/fbioe.2022.956563 36225602 PMC9548561

[B77] Davies R. T. Kim J. Jang S. C. Choi E.-J. Gho Y. S. Park J. (2012). Microfluidic filtration system to isolate extracellular vesicles from blood. Lab a Chip 12, 5202–5210. 10.1039/c2lc41006k 23111789

[B78] De Godoy M. A. Saraiva L. M. De Carvalho L. R. P. Vasconcelos-Dos-Santos A. Beiral H. J. V. Ramos A. B. (2018). Mesenchymal stem cells and cell-derived extracellular vesicles protect hippocampal neurons from oxidative stress and synapse damage induced by amyloid-β oligomers. J. Biol. Chem. 293, 1957–1975. 10.1074/jbc.M117.807180 29284679 PMC5808759

[B79] Dehghani L. Owliaee I. Sadeghian F. Shojaeian A. (2024a). The therapeutic potential of human umbilical cord mesenchymal stromal cells derived exosomes for wound healing: harnessing exosomes as a cell-free therapy. J. Stem Cells Regen. Med. 20, 14–23. 10.46582/jsrm.2003003 39044811 PMC11262847

[B80] Dehghani P. Varshosaz J. Mirian M. Minaiyan M. Kazemi M. Bodaghi M. (2024b). Keratinocyte exosomes for topical delivery of tofacitinib in treatment of psoriasis: an *in vitro/in vivo* study in animal model of psoriasis. Pharm. Res. 41, 263–279. 10.1007/s11095-023-03648-0 38263341 PMC10879239

[B81] del Pozo-Acebo L. López De Las Hazas M. C. Tomé-Carneiro J. Gil-Cabrerizo P. San-Cristobal R. Busto R. (2021). Bovine milk-derived exosomes as a drug delivery vehicle for miRNA-based therapy. Int. J. Mol. Sci. 22, 1105. 10.3390/ijms22031105 33499350 PMC7865385

[B82] Deng J. Zhou M. Liao T. Kuang W. Xia H. Yin Z. (2022). Targeting cancer cell ferroptosis to reverse immune checkpoint inhibitor therapy resistance. Front. cell Dev. Biol. 10, 818453. 10.3389/fcell.2022.818453 35399527 PMC8988234

[B83] Desdín-Micó G. Mittelbrunn M. (2017). Role of exosomes in the protection of cellular homeostasis. Cell adh. Migr. 11, 127–134. 10.1080/19336918.2016.1251000 27875097 PMC5351736

[B84] Dézsi L. Fülöp T. Mészáros T. Szénási G. Urbanics R. Vázsonyi C. (2014). Features of complement activation-related pseudoallergy to liposomes with different surface charge and PEGylation: comparison of the porcine and rat responses. J. Control. release 195, 2–10. 10.1016/j.jconrel.2014.08.009 25148822

[B85] Dhodapkar R. M. Jung E. Lee S. Y. (2025). An eye on extracellular vesicles: trends and clinical translations in vision research. Ophthalmol. Sci. 5, 100619. 10.1016/j.xops.2024.100619 39584184 PMC11585720

[B86] Ding B. Sun W. Han S. Cai Y. Ren M. Shen Y. (2018). Cytochrome P450 1A1 gene polymorphisms and cervical cancer risk: a systematic review and meta-analysis. Medicine 97, e0210. 10.1097/MD.0000000000010210 29595663 PMC5895380

[B87] Ding C. Zhu L. Shen H. Lu J. Zou Q. Huang C. (2020). Exosomal miRNA-17-5p derived from human umbilical cord mesenchymal stem cells improves ovarian function in premature ovarian insufficiency by regulating SIRT7. Stem Cells 38, 1137–1148. 10.1002/stem.3204 32442343

[B88] Dong E. Zhou Z. Chen T. Zhang B. Yin Y. Wu X. (2025). Single-cell sequencing uncovers disrupted stromal-macrophage communication as a driver of intrauterine adhesion progression. Commun. Biol. 8, 1194. 10.1038/s42003-025-08634-3 40790346 PMC12340122

[B89] Donoso‐Quezada J. Ayala‐Mar S. González‐Valdez J. (2021a). The role of lipids in exosome biology and intercellular communication: function, analytics and applications. Traffic 22, 204–220. 10.1111/tra.12803 34053166 PMC8361711

[B90] Donoso‐Quezada J. Ayala‐Mar S. González‐Valdez J. J. T. (2021b). The role of lipids in exosome biology and intercellular communication: function. Anal. Appl. 22, 204–220. 10.1111/tra.12803 34053166 PMC8361711

[B91] Dorayappan K. D. P. Wallbillich J. J. Cohn D. E. Selvendiran K. (2016). The biological significance and clinical applications of exosomes in ovarian cancer. Gynecol. Oncol. 142, 199–205. 10.1016/j.ygyno.2016.03.036 27058839 PMC4917458

[B92] Driedonks T. Jiang L. Carlson B. Han Z. Liu G. Queen S. E. (2022). Pharmacokinetics and biodistribution of extracellular vesicles administered intravenously and intranasally to Macaca nemestrina. J. Extracell. Biol. 1, e59. 10.1002/jex2.59 36591537 PMC9799283

[B93] D’anca M. Fenoglio C. Serpente M. Arosio B. Cesari M. Scarpini E. A. (2019). Exosome determinants of physiological aging and age-related neurodegenerative diseases. Front. Aging Neurosci. 11, 232. 10.3389/fnagi.2019.00232 31555123 PMC6722391

[B94] Echeng N. Burrell D. Zaluski K. (2024). Optimizing operative hysteroscopy in the office setting: updated techniques and technology. Top. Obstetrics and Gynecol. 44, 1–7. 10.1097/01.pgo.0000998088.06847.5a

[B95] El Fekih R. Franzen K. Hurley J. Haynes B. C. Merhej T. Alghamdi A. (2025). An exosomal mRNA urine test for detection and risk stratification of human kidney transplant rejection. Kidney Int. Rep. 10, 1131–1142. 10.1016/j.ekir.2025.01.036 40303229 PMC12034883

[B96] Elmallah M. I. Y. Ortega‐Deballon P. Hermite L. Barros J. P. Gobbo J. Garrido C. (2022). Lipidomic profiling of exosomes from colorectal cancer cells and patients reveals potential biomarkers. Mol. Oncol. 16, 2710–2718. 10.1002/1878-0261.13223 35524452 PMC9298677

[B97] Elsharkasy O. M. Nordin J. Z. Hagey D. W. de Jong O. G. Schiffelers R. M. Andaloussi S. E. L. (2020). Extracellular vesicles as drug delivery systems: why and how? Adv. drug Deliv. Rev. 159, 332–343. 10.1016/j.addr.2020.04.004 32305351

[B98] Emam S. E. Elsadek N. E. Lila A. S. A. Takata H. Kawaguchi Y. Shimizu T. (2021). Anti-PEG IgM production and accelerated blood clearance phenomenon after the administration of PEGylated exosomes in mice. J. Control. Release 334, 327–334. 10.1016/j.jconrel.2021.05.001 33957196

[B99] Emami F. Keihan Shokooh M. Mostafavi Yazdi S. J. (2023). Recent progress in drying technologies for improving the stability and delivery efficiency of biopharmaceuticals. J. Pharm. Investigation 53, 35–57. 10.1007/s40005-022-00610-x 36568503 PMC9768793

[B100] Escudier B. Dorval T. Chaput N. André F. Caby M.-P. Novault S. (2005). Vaccination of metastatic melanoma patients with autologous dendritic cell (DC) derived-exosomes: results of thefirst phase I clinical trial. J. Transl. Med. 3, 10–13. 10.1186/1479-5876-3-10 15740633 PMC554765

[B101] Fabbri M. Paone A. Calore F. Galli R. Gaudio E. Santhanam R. (2012). MicroRNAs bind to toll-like receptors to induce prometastatic inflammatory response. Proc. Natl. Acad. Sci. U. S. A. 109, E2110–E2116. 10.1073/pnas.1209414109 22753494 PMC3412003

[B102] Fan J.-T. Zhou Z.-Y. Luo Y.-L. Luo Q. Chen S.-B. Zhao J.-C. (2021). Exosomal lncRNA NEAT1 from cancer-associated fibroblasts facilitates endometrial cancer progression *via* miR-26a/b-5p-mediated STAT3/YKL-40 signaling pathway. Neoplasia 23, 692–703. 10.1016/j.neo.2021.05.004 34153644 PMC8233173

[B103] Fan M. Xiong X. Han L. Zhang L. Gao S. Liu L. (2022). SERPINA5 promotes tumour cell proliferation by modulating the PI3K/AKT/mTOR signalling pathway in gastric cancer. J. Cell. Mol. Med. 26, 4837–4846. 10.1111/jcmm.17514 36000536 PMC9465189

[B104] Fang Y. Q. Zhang H. K. Wei Q. Q. Li Y. H. (2024). Brown adipose tissue‐derived exosomes improve polycystic ovary syndrome in mice *via* STAT3/GPX4 signaling pathway. FASEB J. 38, e70062. 10.1096/fj.202401346R 39305125

[B105] Farzanehpour M. Miri A. Alvanegh A. G. Gouvarchinghaleh H. E. (2023). Viral vectors, exosomes, and vexosomes: potential armamentarium for delivering CRISPR/cas to cancer cells. Biochem. Pharmacol. 212, 115555. 10.1016/j.bcp.2023.115555 37075815

[B106] Fazeli A. Godakumara K. (2024). The evolving roles of extracellular vesicles in embryo-maternal communication. Commun. Biol. 7, 754. 10.1038/s42003-024-06442-9 38906986 PMC11192758

[B107] Feng J. Wang J. Zhang Y. Zhang Y. Jia L. Zhang D. (2021). The efficacy of complementary and alternative medicine in the treatment of female infertility. Evidence‐Based Complementary Altern. Med. 2021, 6634309. 10.1155/2021/6634309 33986820 PMC8093064

[B108] Fernandes R. P. Ruiz A. B. Bezemer S. Detmers F. Hermans P. Peixoto C. (2025). Targeted isolation of extracellular vesicles from cell culture supernatant using immuno-affinity chromatography. Sep. Purif. Technol. 358, 130312. 10.1016/j.seppur.2024.130312

[B109] Fernández L. Castro I. Arroyo R. Alba C. Beltrán D. Rodríguez J. M. (2021). Application of Ligilactobacillus salivarius CECT5713 to achieve term pregnancies in women with repetitive abortion or infertility of unknown origin by microbiological and immunological modulation of the vaginal ecosystem. Nutrients 13, 162. 10.3390/nu13010162 33419054 PMC7825435

[B110] Filipe V. Hawe A. Jiskoot W. (2010). Critical evaluation of nanoparticle tracking analysis (NTA) by NanoSight for the measurement of nanoparticles and protein aggregates. Pharm. Res. 27, 796–810. 10.1007/s11095-010-0073-2 20204471 PMC2852530

[B111] Fordjour F. K. Guo C. Ai Y. Daaboul G. G. Gould S. J. (2022). A shared, stochastic pathway mediates exosome protein budding along plasma and endosome membranes. J. Biol. Chem. 298, 102394. 10.1016/j.jbc.2022.102394 35988652 PMC9512851

[B112] Freger S. Leonardi M. Foster W. G. (2021). Exosomes and their cargo are important regulators of cell function in endometriosis. Reprod. Biomed. Online 43, 370–378. 10.1016/j.rbmo.2021.05.022 34272164

[B113] Galbiati S. Damin F. Brambilla D. Ferraro L. Soriani N. Ferretti A. M. (2021). Small EVs-associated DNA as complementary biomarker to circulating tumor DNA in plasma of metastatic colorectal cancer patients. Pharmaceuticals 14, 128. 10.3390/ph14020128 33562158 PMC7915475

[B114] Ganesh G. V. Gayathri B. Jayasuriya R. Ramkumar K. M. (2025). Exosomal miR16 induced by allyl isothiocyanate (AITC) inhibits tumor growth in cervical cancer *via* modulation of apoptotic and inflammatory pathways. Archives Biochem. Biophysics 770, 110446. 10.1016/j.abb.2025.110446 40315946

[B115] Gao M. Cai J. Zitkovsky H. S. Chen B. Guo L. (2022). Comparison of yield, purity, and functional properties of large-volume exosome isolation using ultrafiltration and polymer-based precipitation. Plastic Reconstr. Surg. 149, 638–649. 10.1097/PRS.0000000000008830 35196679

[B116] Gaona-Luviano P. Medina-Gaona L. A. Magaña-Pérez K. (2020). Epidemiology of ovarian cancer. arXiv 9, 47–47. 32648448 10.21037/cco-20-34

[B117] Garaeva L. Kamyshinsky R. Kil Y. Varfolomeeva E. Verlov N. Komarova E. (2021). Delivery of functional exogenous proteins by plant-derived vesicles to human cells *in vitro* . Sci. Rep. 11, 6489. 10.1038/s41598-021-85833-y 33753795 PMC7985202

[B118] Ghafouri-Fard S. Niazi V. Hussen B. M. Omrani M. D. Taheri M. Basiri A. J. F. I. C. (2021). The emerging role of exosomes in the treatment of human disorders with a special focus on mesenchymal stem cells-derived exosomes. Front. Cell Dev. Biol. 9, 653296. 10.3389/fcell.2021.653296 34307345 PMC8293617

[B119] Ghahremani-Nasab M. Babaie S. Bazdar S. Paiva-Santos A. C. Del Bakhshayesh M. R. Akbari-Gharalari N. (2025). Infertility treatment using polysaccharides-based hydrogels: new strategies in tissue engineering and regenerative medicine. J. Nanobiotechnology 23, 162. 10.1186/s12951-025-03267-4 40033394 PMC11877900

[B120] Ghajari G. Heydari A. Ghorbani M. (2023). Mesenchymal stem cell-based therapy and female infertility: limitations and advances. Curr. Stem Cell Res. Ther. 18, 322–338. 10.2174/1574888X17666220511142930 35546752

[B121] Ghasroldasht M. M. Park H.-S. Ali F. L. Beckman A. Mohammadi M. Hafner N. (2025). Adapted exosomes for addressing chemotherapy-induced premature ovarian insufficiency. Stem Cell Rev. Rep. 21, 779–796. 10.1007/s12015-024-10820-5 39921838

[B122] Göhner C. Plösch T. Faas M. M. (2017). Immune-modulatory effects of syncytiotrophoblast extracellular vesicles in pregnancy and preeclampsia. Placenta 60, S41–S51. 10.1016/j.placenta.2017.06.004 28647398

[B123] Grange C. Tapparo M. Collino F. Vitillo L. Damasco C. Deregibus M. C. (2011). Microvesicles released from human renal cancer stem cells stimulate angiogenesis and formation of lung premetastatic niche. Cancer Res. 71, 5346–5356. 10.1158/0008-5472.CAN-11-0241 21670082

[B124] Gul R. Bashir H. Sarfraz M. Shaikh A. J. Jardan Y. A. B. Hussain Z. (2024). Human plasma derived exosomes: impact of active and passive drug loading approaches on drug delivery. Saudi Pharm. J. 32, 102096. 10.1016/j.jsps.2024.102096 38757071 PMC11097067

[B125] Guo S. Perets N. Betzer O. Ben-Shaul S. Sheinin A. Michaelevski I. (2019). Intranasal delivery of mesenchymal stem cell derived exosomes loaded with phosphatase and tensin homolog siRNA repairs complete spinal cord injury. ACS nano 13, 10015–10028. 10.1021/acsnano.9b01892 31454225

[B126] Guo W. Qiao T. Dong B. Li T. Liu Q. Xu X. (2022). The effect of hypoxia-induced exosomes on anti-tumor immunity and its implication for immunotherapy. Front. Immunol. 13, 915985. 10.3389/fimmu.2022.915985 35812406 PMC9257077

[B127] Gurung S. Greening D. Catt S. Salamonsen L. Evans J. (2020). Exosomes and soluble secretome from hormone-treated endometrial epithelial cells direct embryo implantation. Mol. Hum. Reprod. 26, 510–520. 10.1093/molehr/gaaa034 32402079

[B128] Hadidi M. Karimabadi K. Ghanbari E. Rezakhani L. Khazaei M. (2023). Stem cells and exosomes: as biological agents in the diagnosis and treatment of polycystic ovary syndrome (PCOS). Front. Endocrinol. 14, 1269266. 10.3389/fendo.2023.1269266 37964963 PMC10642184

[B129] Hajipour H. Farzadi L. Roshangar L. Latifi Z. Kahroba H. Shahnazi V. (2021). A human chorionic gonadotropin (hCG) delivery platform using engineered uterine exosomes to improve endometrial receptivity. Life Sci. 275, 119351. 10.1016/j.lfs.2021.119351 33737084

[B130] Han Q. Du Y. (2020). Advances in the application of biomimetic endometrium interfaces for uterine bioengineering in female infertility. Front. Bioeng. Biotechnol. 8, 153. 10.3389/fbioe.2020.00153 32181248 PMC7059418

[B131] Han J. Wu T. Jin J. Li Z. Cheng W. Dai X. (2022). Exosome-like nanovesicles derived from Phellinus linteus inhibit Mical2 expression through cross-kingdom regulation and inhibit ultraviolet-induced skin aging. J. nanobiotechnology 20, 455. 10.1186/s12951-022-01657-6 36271377 PMC9587628

[B132] He L. Liu L. Li T. Zhuang D. Dai J. Wang B. (2021). Exploring the imbalance of periodontitis immune system from the cellular to molecular level. Front. Genet. 12, 653209. 10.3389/fgene.2021.653209 33841510 PMC8033214

[B133] He W. Zhu X. Xin A. Zhang H. Sun Y. Xu H. (2022). Long-term maintenance of human endometrial epithelial stem cells and their therapeutic effects on intrauterine adhesion. Cell and Biosci. 12, 175. 10.1186/s13578-022-00905-4 36258228 PMC9580151

[B134] He X. Wang Y. Liu Z. Weng Y. Chen S. Pan Q. (2023). Osteoporosis treatment using stem cell-derived exosomes: a systematic review and meta-analysis of preclinical studies. Stem cell Res. and Ther. 14, 72. 10.1186/s13287-023-03317-4 37038180 PMC10088147

[B135] Hendijani F. (2017). Explant culture: an advantageous method for isolation of mesenchymal stem cells from human tissues. Cell Prolif. 50, e12334. 10.1111/cpr.12334 28144997 PMC6529062

[B136] Honda T. Kawabori M. Fujimura M. (2025). Intra-arterial administration of stem cells and exosomes for central nervous system disease. Int. J. Mol. Sci. 26, 7405. 10.3390/ijms26157405 40806534 PMC12347363

[B137] Hosseinkhani B. Van Den Akker N. M. S. Molin D. G. M. Michiels L. (2020). (sub) populations of extracellular vesicles released by TNF-α–triggered human endothelial cells promote vascular inflammation and monocyte migration. J. Extracell. vesicles 9, 1801153. 10.1080/20013078.2020.1801153 32944190 PMC7480596

[B138] Hou Z. Yang T. Xu D. Fu J. Tang H. Zhao J. (2025). hUC-MSCs loaded collagen scaffold for refractory thin endometrium caused by asherman syndrome: a double-blind randomized controlled trial. Stem Cells Transl. Med. 14, szaf011. 10.1093/stcltm/szaf011 40371958 PMC12079654

[B139] Hromadnikova I. Kotlabova K. Ondrackova M. Pirkova P. Kestlerova A. Novotna V. (2015). Expression profile of C19MC microRNAs in placental tissue in pregnancy-related complications. DNA cell Biol. 34, 437–457. 10.1089/dna.2014.2687 25825993 PMC4486149

[B140] Hua R. Liu Q. Lian W. Kang T. T. Gao D. Huang C. (2022). Extracellular vesicles derived from endometrial epithelial cells deliver exogenous miR-92b-3p to affect the function of embryonic trophoblast cells *via* targeting TSC1 and DKK3. Reprod. Biol. Endocrinol. 20, 152. 10.1186/s12958-022-01023-z 36284344 PMC9594956

[B141] Huang S. Ji X. Jackson K. K. Lubman D. M. Ard M. B. Bruce T. F. (2021). Rapid separation of blood plasma exosomes from low-density lipoproteins *via* a hydrophobic interaction chromatography method on a polyester capillary-channeled polymer fiber phase. Anal. Chim. acta 1167, 338578. 10.1016/j.aca.2021.338578 34049630 PMC8164660

[B142] Huang Q.-Y. Chen S.-R. Chen J.-M. Shi Q.-Y. Lin S. J. R. B. (2022a). Therapeutic options for premature ovarian insufficiency: an updated review. Endocrinology 20, 28. 10.1186/s12958-022-00892-8 35120535 PMC8815154

[B143] Huang Y. Zhu L. Li H. Ye J. Lin N. Chen M. (2022b). Endometriosis derived exosomal miR-301a-3p mediates macrophage polarization *via* regulating PTEN-PI3K axis. Biomed. and Pharmacother. 147, 112680. 10.1016/j.biopha.2022.112680 35124383

[B144] Hung W.-T. Hong X. Christenson L. K. Mcginnis L. K. (2015). Extracellular vesicles from bovine follicular fluid support cumulus expansion. Biol. Reprod. 93 (117), 117–119. 10.1095/biolreprod.115.132977 26423123 PMC4712005

[B145] Huo C. Zhao Y. Song M. Guo Y. Li S. Tan W. (2025). Three-dimensional and serum-free culture in fixed-bed bioreactor enhance exosome production by affecting the cytoskeleton through integrin β1 and RAC1. Biochem. Eng. J. 220, 109750. 10.1016/j.bej.2025.109750

[B146] Jahanbani Y. Davaran S. Ghahremani-Nasab M. Aghebati-Maleki L. Yousefi M. (2020). Scaffold-based tissue engineering approaches in treating infertility. Life Sci. 240, 117066. 10.1016/j.lfs.2019.117066 31738881

[B147] Jang J. Jeong H. Jang E. Kim E. Yoon Y. Jang S. (2023). Isolation of high-purity and high-stability exosomes from ginseng. Front. Plant Sci. 13, 1064412. 10.3389/fpls.2022.1064412 36714697 PMC9878552

[B148] Jaremek A. Jeyarajah M. J. Jaju Bhattad G. Renaud S. J. (2021). Omics approaches to study formation and function of human placental syncytiotrophoblast. Front. Cell Dev. Biol. 9, 674162. 10.3389/fcell.2021.674162 34211975 PMC8240757

[B149] Ji J. Wang H. Yuan M. Li J. Song X. Lin K. (2024). Exosomes from ectopic endometrial stromal cells promote M2 macrophage polarization by delivering miR-146a-5p. Int. Immunopharmacol. 128, 111573. 10.1016/j.intimp.2024.111573 38278065

[B150] Jia J. Guo S. Zhang D. Tian X. Xie X. (2020). Exosomal-lncRNA DLEU1 accelerates the proliferation, migration, and invasion of endometrial carcinoma cells by regulating microRNA-E2F3. OncoTargets Ther. 13, 8651–8663. 10.2147/OTT.S262661 32904666 PMC7457553

[B151] Jiang Y. Luo T. Xia Q. Tian J. Yang J. (2022). microRNA-140-5p from human umbilical cord mesenchymal stem cells–released exosomes suppresses preeclampsia development. Funct. and Integr. Genomics 22, 813–824. 10.1007/s10142-022-00848-6 35484307

[B152] Jiang X. Zhang Z. Hou M. Yang X. Cui L. (2023). Plasma exosomes and contained MiRNAs affect the reproductive phenotype in polycystic ovary syndrome. FASEB J. 37, e22960. 10.1096/fj.202201940RR 37335566

[B153] Jin X. Dai Y. Xin L. Ye Z. Chen J. He Q. (2023). ADSC-Derived exosomes-coupled decellularized matrix for endometrial regeneration and fertility restoration. Mater. Today Bio 23, 100857. 10.1016/j.mtbio.2023.100857 38075259 PMC10709080

[B154] Jing L. Hua X. Yuanna D. Rukun Z. Junjun M. (2020). Exosomal miR-499a-5p inhibits endometrial cancer growth and metastasis *via* targeting VAV3. Cancer Manag. Res. 12, 13541–13552. 10.2147/CMAR.S283747 33408524 PMC7781017

[B155] Johnson J. Law S. Q. K. Shojaee M. Hall A. S. Bhuiyan S. Lim M. B. L. (2023). First‐in‐human clinical trial of allogeneic, platelet‐derived extracellular vesicles as a potential therapeutic for delayed wound healing. J. Extracell. vesicles 12, 12332. 10.1002/jev2.12332 37353884 PMC10290200

[B156] Johnstone R. M. (2020). The transferrin receptor in Red blood cell membranes (Boca Raton: CRC Press). 10.1201/9781003066118

[B157] Jokhio S. Peng I. Peng C.-A. (2024). Extracellular vesicles isolated from *Arabidopsis thaliana* leaves reveal characteristics of Mammalian exosomes. 1-9. 10.1007/s00709-024-01954-x 38683390

[B158] Ju W. Pan K. Zhang Q. Wang Y. Zhao S. Zhang J. (2025). Differential expression of microRNA in follicular fluid-derived extracellular vesicles and mRNA in granulosa cells of patients with polycystic ovary syndrome and insulin resistance. Reprod. Biomed. Online, 105027. 10.1016/j.rbmo.2025.105027 40907075

[B159] Jung H. J. Suh Y. (2015). Regulation of IGF-1 signaling by microRNAs. Front. Genet. 5, 472. 10.3389/fgene.2014.00472 25628647 PMC4292735

[B160] Kamerkar S. Lebleu V. S. Sugimoto H. Yang S. Ruivo C. F. Melo S. A. (2017). Exosomes facilitate therapeutic targeting of oncogenic KRAS in pancreatic cancer. Nature 546, 498–503. 10.1038/nature22341 28607485 PMC5538883

[B161] Kanwar S. S. Dunlay C. J. Simeone D. M. Nagrath S. (2014). Microfluidic device (ExoChip) for on-chip isolation, quantification and characterization of circulating exosomes. Lab a Chip 14, 1891–1900. 10.1039/c4lc00136b 24722878 PMC4134440

[B162] Karkia R. Maccarthy G. Payne A. Karteris E. Pazoki R. Chatterjee J. (2025). The association between metabolic syndrome and the risk of endometrial cancer in pre-and post-menopausal women: a UK biobank study. J. Clin. Med. 14, 751. 10.3390/jcm14030751 39941422 PMC11818266

[B163] Kataki A. C. Tiwari P. Thilagavthi R. Krishnatreya M. (2023). Epidemiology of gynaecological cancers. Fundam. Gynaecol. Malignancy, 1–8. 10.1007/978-981-19-5860-1_1

[B164] Katre D. Choudhary N. Anjankar A. P. More A. Dakre S. (2024). The role of GnRH agonist therapy and hysterosalpingography (HSG) in diagnosing and treating tubal factor infertility. J. Pharm. Bioallied Sci. 16, S4013–S4016. 10.4103/jpbs.jpbs_1400_24 39926785 PMC11805295

[B165] Khan Z. (2023). Etiology, risk factors, and management of asherman syndrome. Obstetrics and Gynecol. 142, 543–554. 10.1097/AOG.0000000000005309 37490750

[B166] Kim J. Shin H. Kim J. Kim J. Park J. (2015). Isolation of high-purity extracellular vesicles by extracting proteins using aqueous two-phase system. PloS one 10, e0129760. 10.1371/journal.pone.0129760 26090684 PMC4475045

[B167] Kim H. J. Kim G. Lee J. Lee Y. Kim J.-H. (2022a). Secretome of stem cells: roles of extracellular vesicles in diseases, stemness, differentiation, and reprogramming. Tissue Eng. Regen. Med. 19, 19–33. 10.1007/s13770-021-00406-4 34817808 PMC8782975

[B168] Kim J. Lee Y.-H. Wang J. Kim Y. K. Kwon I. K. (2022b). Isolation and characterization of ginseng-derived exosome-like nanoparticles with sucrose cushioning followed by ultracentrifugation. SN Appl. Sci. 4, 63. 10.1007/s42452-022-04943-y

[B169] Kim T. Choodinatha H. K. Kim K. S. Shin K. Kim H. J. Park J. Y. (2024). Understanding the role of soluble proteins and exosomes in non-invasive urine-based diagnosis of preeclampsia. Sci. Rep. 14, 24117. 10.1038/s41598-024-75080-2 39406891 PMC11482518

[B170] King A. Ndifon C. Lui S. Widdows K. Kotamraju V. R. Agemy L. (2016). Tumor-homing peptides as tools for targeted delivery of payloads to the placenta. Sci. Adv. 2, e1600349. 10.1126/sciadv.1600349 27386551 PMC4928982

[B171] Klymiuk M. C. Balz N. Elashry M. I. Wenisch S. Arnhold S. (2024). Effect of storage conditions on the quality of equine and canine mesenchymal stem cell derived nanoparticles including extracellular vesicles for research and therapy. Discov. Nano 19, 80–14. 10.1186/s11671-024-04026-4 38700790 PMC11068712

[B172] Kobayashi M. Sawada K. Miyamoto M. Shimizu A. Yamamoto M. Kinose Y. (2020). Exploring the potential of engineered exosomes as delivery systems for tumor-suppressor microRNA replacement therapy in ovarian cancer. Biochem. Biophys. Res. Commun. 527, 153–161. 10.1016/j.bbrc.2020.04.076 32446360

[B173] Kocarnik J. M. Compton K. Dean F. E. Fu W. Gaw B. L. Harvey J. D. (2022). Cancer incidence, mortality, years of life lost, years lived with disability, and disability-adjusted life years for 29 cancer groups from 2010 to 2019: a systematic analysis for the global burden of disease study 2019. JAMA Oncol. 8, 420–444. 10.1001/jamaoncol.2021.6987 34967848 PMC8719276

[B174] Koike H. Harada M. Kusamoto A. Xu Z. Tanaka T. Sakaguchi N. (2023). Roles of endoplasmic reticulum stress in the pathophysiology of polycystic ovary syndrome. Front. Endocrinol. 14, 1124405. 10.3389/fendo.2023.1124405 36875481 PMC9975510

[B175] Konoshenko M. Y. Lekchnov E. A. Vlassov A. V. Laktionov P. P. (2018). Isolation of extracellular vesicles: general methodologies and latest trends. Biomed. Res. Int. 2018, 8545347. 10.1155/2018/8545347 29662902 PMC5831698

[B176] Kowal J. Tkach M. Biology M. (2019). Dendritic cell extracellular vesicles. Int. Rev. Cell Mol. Biol. 349, 213–249. 10.1016/bs.ircmb.2019.08.005 31759432

[B177] Kumar D. N. Chaudhuri A. Kumar D. Singh S. Agrawal A. K. (2023a). Impact of the drug loading method on the drug distribution and biological efficacy of exosomes. Aaps Pharmscitech 24, 166. 10.1208/s12249-023-02624-6 37552397

[B178] Kumar M. N. Kalarikkal S. P. Bethi C. M. S. Singh S. N. Narayanan J. Sundaram G. M. (2023b). An eco-friendly one-pot extraction process for curcumin and its bioenhancer, piperine, from edible plants in exosome-like nanovesicles. Green Chem. 25, 6472–6488. 10.1039/d3gc01287e

[B179] Kverneland A. H. Østergaard O. Emdal K. B. Svane I. M. Olsen J. V. (2023). Differential ultracentrifugation enables deep plasma proteomics through enrichment of extracellular vesicles. Proteomics 23, 2200039. 10.1002/pmic.202200039 36398564

[B180] Kwizera E. A. Sun M. White A. M. Li J. He X. (2021). Methods of generating dielectrophoretic force for microfluidic manipulation of bioparticles. ACS biomaterials Sci. and Eng. 7, 2043–2063. 10.1021/acsbiomaterials.1c00083 33871975 PMC8205986

[B181] Le Gall L. Ouandaogo Z. G. Anakor E. Connolly O. Butler Browne G. Laine J. (2020). Optimized method for extraction of exosomes from human primary muscle cells. Skelet. Muscle 10, 20–13. 10.1186/s13395-020-00238-1 32641118 PMC7341622

[B182] Lee M. Ban J.-J. Im W. Kim M. (2016). Influence of storage condition on exosome recovery. Biotechnol. Bioprocess Eng. 21, 299–304. 10.1007/s12257-015-0781-x

[B183] Lee D.-H. Yun D. W. Kim Y. H. Im G.-B. Hyun J. Park H. S. (2023). Various three-dimensional culture methods and cell types for exosome production. Tissue Eng. Regen. Med. 20, 621–635. 10.1007/s13770-023-00551-y 37269439 PMC10313642

[B184] Lee C.-L. Vu C.-A. Vu V.-T. Cheng C.-M. Chang Y. Chen W.-Y. (2024). Integrating sulfobetaine methacrylate hydrogel-modified cellulose triacetate filter into a zwitterionized tandem membrane system for exosome isolation. ACS Appl. Polym. Mater. 6, 2741–2751. 10.1021/acsapm.3c02949

[B185] Levy D. Jeyaram A. Born L. J. Chang K.-H. Abadchi S. N. Hsu A. T. W. (2023). Impact of storage conditions and duration on function of native and cargo-loaded mesenchymal stromal cell extracellular vesicles. Cytotherapy 25, 502–509. 10.1016/j.jcyt.2022.11.006 36513574 PMC10079553

[B186] Li X. Wang X. (2017). The emerging roles and therapeutic potential of exosomes in epithelial ovarian cancer. Mol. Cancer 16, 92–10. 10.1186/s12943-017-0659-y 28506269 PMC5433006

[B187] Li J. Ghazwani M. Zhang Y. Lu J. Li J. Fan J. (2013). miR-122 regulates collagen production *via* targeting hepatic stellate cells and suppressing P4HA1 expression. J. hepatology 58, 522–528. 10.1016/j.jhep.2012.11.011 23178710 PMC3619187

[B188] Li X. Liu L. L. Yao J. L. Wang K. Ai H. (2019). Human umbilical cord mesenchymal stem cell‐derived extracellular vesicles inhibit endometrial cancer cell proliferation and migration through delivery of exogenous miR‐302a. Stem cells Int. 2019, 8108576. 10.1155/2019/8108576 31001342 PMC6437733

[B189] Li P. Xin H. Lu L. (2021a). Extracellular vesicle-encapsulated microRNA-424 exerts inhibitory function in ovarian cancer by targeting MYB. J. Transl. Med. 19, 4–17. 10.1186/s12967-020-02652-x 33407591 PMC7786507

[B190] Li Z. Zhang M. Zheng J. Tian Y. Zhang H. Tan Y. (2021b). Human umbilical cord mesenchymal stem cell-derived exosomes improve ovarian function and proliferation of premature ovarian insufficiency by regulating the hippo signaling pathway. Front. Endocrinol. 12, 711902. 10.3389/fendo.2021.711902 34456868 PMC8397419

[B191] Li C. Li X. Shi Z. Wu P. Fu J. Tang J. (2022a). Exosomes from LPS-Preconditioned bone marrow MSCs accelerated peripheral nerve regeneration *via* M2 macrophage polarization: involvement of TSG-6/NF-κB/NLRP3 signaling pathway. Exp. Neurol. 356, 114139. 10.1016/j.expneurol.2022.114139 35690131

[B192] Li D. Yao X. Yue J. Fang Y. Cao G. Midgley A. C. (2022b). Advances in bioactivity of microRNAs of plant-derived exosome-like nanoparticles and milk-derived extracellular vesicles. J. Agric. Food Chem. 70, 6285–6299. 10.1021/acs.jafc.2c00631 35583385

[B193] Li L. He D. Guo Q. Zhang Z. Ru D. Wang L. (2022c). Exosome-liposome hybrid nanoparticle codelivery of TP and miR497 conspicuously overcomes chemoresistant ovarian cancer. J. Nanobiotechnology 20, 50. 10.1186/s12951-022-01264-5 35078498 PMC8787930

[B194] Li W.-J. Chen H. Tong M.-L. Niu J.-J. Zhu X.-Z. Lin L.-R. (2022d). Comparison of the yield and purity of plasma exosomes extracted by ultracentrifugation, precipitation, and membrane-based approaches. Open Chem. 20, 182–191. 10.1515/chem-2022-0139

[B195] Li Y. Wu J. Li E. Xiao Z. Lei J. Zhou F. (2022e). TP53 mutation detected in circulating exosomal DNA is associated with prognosis of patients with hepatocellular carcinoma. Cancer Biol. and Ther. 23, 439–445. 10.1080/15384047.2022.2094666 35921289 PMC9354767

[B196] Li Q. Hu W. Huang Q. Yang J. Li B. Ma K. (2023a). MiR146a-loaded engineered exosomes released from silk fibroin patch promote diabetic wound healing by targeting IRAK1. Signal Transduct. Target. Ther. 8, 62. 10.1038/s41392-022-01263-w 36775818 PMC9922687

[B197] Li X. Duan H. Wang S. Lv C.-X. (2023b). Umbilical cord mesenchymal stem cell-derived exosomes reverse endometrial fibrosis by the miR-145-5p/ZEB2 axis in intrauterine adhesions. Reprod. Biomed. Online 46, 234–243. 10.1016/j.rbmo.2022.05.018 36567149

[B198] Li L. An J. Wang Y. Liu L. Wang Y. Zhang X. (2024a). Exosomes derived from mesenchymal stem cells increase the viability of damaged endometrial cells *via* the miR-99b-5p/PCSK9 axis. Stem Cells Dev. 33, 290–305. 10.1089/scd.2023.0259 38573013

[B199] Li Q. Zhang Z. Shi W. Li Z. Xiao Y. Zhang J. (2024b). Drug-free *in vitro* activation combined with ADSCs-derived exosomes restores ovarian function of rats with premature ovarian insufficiency. J. Ovarian Res. 17, 158. 10.1186/s13048-024-01475-4 39085868 PMC11290131

[B200] Li Y. Zhang H. Cai C. Mao J. Li N. Huang D. (2024c). Microfluidic encapsulation of exosomes derived from lipopolysaccharide‐treated mesenchymal stem cells in hyaluronic acid methacryloyl to restore ovarian function in mice. Adv. Healthc. Mater. 13, 2303068. 10.1002/adhm.202303068 37972286

[B201] Liang X. Zhang L. Wang S. Han Q. Zhao R. C. (2016). Exosomes secreted by mesenchymal stem cells promote endothelial cell angiogenesis by transferring miR-125a. J. Cell Sci. 129, 2182–2189. 10.1242/jcs.170373 27252357

[B202] Liang S. Xu H. Ye B.-C. (2021). Membrane-decorated exosomes for combination drug delivery and improved glioma therapy. Langmuir. 38, 299–308. 10.1021/acs.langmuir.1c02500 34936368

[B203] Liang Y. Shuai Q. Zhang X. Jin S. Guo Y. Yu Z. (2024). Incorporation of decidual stromal cells derived exosomes in sodium alginate hydrogel as an innovative therapeutic strategy for advancing endometrial regeneration and reinstating fertility. Adv. Healthc. Mater. 13, 2303674. 10.1002/adhm.202303674 38315148

[B204] Liao B. Qiao J. Pang Y. (2021). Central regulation of PCOS: abnormal neuronal-reproductive-metabolic circuits in PCOS pathophysiology. Front. Endocrinol. 12, 667422. 10.3389/fendo.2021.667422 34122341 PMC8194358

[B205] Lin B. Lei Y. Wang J. Zhu L. Wu Y. Zhang H. (2021a). Microfluidic‐based exosome analysis for liquid biopsy. Small Methods 5, 2001131. 10.1002/smtd.202001131 34927834

[B206] Lin J. Wang Z. Huang J. Tang S. Saiding Q. Zhu Q. (2021b). Microenvironment‐protected exosome‐hydrogel for facilitating endometrial regeneration, fertility restoration, and live birth of offspring. Small 17, 2007235. 10.1002/smll.202007235 33590681

[B207] Lin L. Huai J. Li B. Zhu Y. Juan J. Zhang M. (2022). A randomized controlled trial of low-dose aspirin for the prevention of preeclampsia in women at high risk in China. Am. J. Obstet. Gynecol. 226, 251.e1–251.e12. 10.1016/j.ajog.2021.08.004 34389292

[B208] Lin Y. Li Y. Chen P. Zhang Y. Sun J. Sun X. (2023). Exosome-based regimen rescues endometrial fibrosis in intrauterine adhesions *via* targeting clinical fibrosis biomarkers. Stem Cells Transl. Med. 12, 154–168. 10.1093/stcltm/szad007 36893290 PMC10021501

[B209] Lin X. Fang Y. Mi X. Fu J. Chen S. Wu M. (2024). Intrauterine injection of bioengineered hydrogel loaded exosomes derived from HUCM stem cells and spermidine prominently augments the pregnancy rate in thin endometrium rats. Regen. Ther. 27, 63–72. 10.1016/j.reth.2024.02.003 38525237 PMC10959642

[B210] Liu F. Hu S. Yang H. Li Z. Huang K. Su T. (2019). Hyaluronic acid hydrogel integrated with mesenchymal stem cell‐secretome to treat endometrial injury in a rat model of Asherman's syndrome. Adv. Healthc. Mater. 8, 1900411. 10.1002/adhm.201900411 31148407 PMC7045702

[B211] Liu D. Wang J. Zhao G. Jiang P. Song M. Ding H. (2020a). CSF1‐associated decrease in endometrial macrophages May contribute to Asherman's syndrome. Am. J. Reprod. Immunol. 83, e13191. 10.1111/aji.13191 31536655

[B212] Liu H. Wang F. Zhang Y. Xing Y. Wang Q. (2020b). Exosomal microRNA‐139‐5p from mesenchymal stem cells accelerates trophoblast cell invasion and migration by motivation of the ERK/MMP‐2 pathway *via* downregulation of protein tyrosine phosphatase. J. Obstetrics Gynaecol. Res. 46, 2561–2572. 10.1111/jog.14495 32945060 PMC7756315

[B213] Liu M. Qiu Y. Xue Z. Wu R. Li J. Niu X. (2020c). Small extracellular vesicles derived from embryonic stem cells restore ovarian function of premature ovarian failure through PI3K/AKT signaling pathway. Stem Cell Res. Ther. 11, 3–12. 10.1186/s13287-019-1508-2 31900201 PMC6942273

[B214] Liu Y. Wang Y. Lv Q. Li X. (2020d). Exosomes: from garbage bins to translational medicine. Int. J. Pharm. 583, 119333. 10.1016/j.ijpharm.2020.119333 32348800

[B215] Liu A. Lin D. Zhao H. Chen L. Cai B. Lin K. (2021). Optimized BMSC-derived osteoinductive exosomes immobilized in hierarchical scaffold *via* lyophilization for bone repair through Bmpr2/Acvr2b competitive receptor-activated Smad pathway. Biomaterials 272, 120718. 10.1016/j.biomaterials.2021.120718 33838528

[B216] Liu W. Chen M. Liu C. Wang L. Wei H. Zhang R. (2023a). Epg5 deficiency leads to primary ovarian insufficiency due to WT1 accumulation in mouse granulosa cells. Autophagy 19, 644–659. 10.1080/15548627.2022.2094671 35786405 PMC9851269

[B217] Liu Y. Yan L. Xixiang M. Wenpei X. (2023b). P-591 Follicular fluid-derived exosomes rejuvenate ovarian aging through miR-320a-3p-mediated FOXQ1 inhibition. Hum. Reprod. 38, dead093.921–921. 10.1093/humrep/dead093.921 PMC11749233 39872394

[B218] Liu F.-J. Zhang Y.-L. Wang X.-S. Zhao Y.-Q. Wang H.-W. (2024a). Role of molybdenum in ameliorating busulfan-induced infertility in female mice. J. Trace Elem. Med. Biol. 86, 127546. 10.1016/j.jtemb.2024.127546 39418757

[B219] Liu H. Zhang X. Zhang M. Zhang S. Li J. Zhang Y. (2024b). Mesenchymal stem cell derived exosomes repair uterine injury by targeting transforming growth factor-β signaling. ACS nano 18, 3509–3519. 10.1021/acsnano.3c10884 38241636

[B220] Liu Z. Zhou Q. Zan J. Tian J. Zhang Y. Wu F. (2025). Proteomic analysis of human follicular fluid-derived exosomes reveals that insufficient folliculogenesis in aging women is associated with infertility. Mol. and Cell. Proteomics 24, 100930. 10.1016/j.mcpro.2025.100930 40024376 PMC11994977

[B221] Lopez de Las Hazas M.-C. Gil-Zamorano J. Cofán M. Mantilla-Escalante D. C. Garcia-Ruiz A. Del Pozo-Acebo L. (2021). One-year dietary supplementation with walnuts modifies exosomal miRNA in elderly subjects. Eur. J. Nutr. 60, 1999–2011. 10.1007/s00394-020-02390-2 32979076

[B222] Lu M. Zhao X. Xing H. Xun Z. Zhu S. Lang L. (2018). Comparison of exosome-mimicking liposomes with conventional liposomes for intracellular delivery of siRNA. Int. J. Pharm. 550, 100–113. 10.1016/j.ijpharm.2018.08.040 30138707

[B223] Lu M. Dibernardo E. Parks E. Fox H. Zheng S.-Y. Wayne E. (2021a). The role of extracellular vesicles in the pathogenesis and treatment of autoimmune disorders. Front. Immunol. 12, 566299. 10.3389/fimmu.2021.566299 33732229 PMC7959789

[B224] Lu Y. Eguchi T. Sogawa C. Taha E. A. Tran M. T. Nara T. (2021b). Exosome-based molecular transfer activity of macrophage-like cells involves viability of oral carcinoma cells: size exclusion chromatography and concentration filter method. Cells 10, 1328. 10.3390/cells10061328 34071980 PMC8228134

[B225] Luo H. Zhou Y. Zhang J. Zhang Y. Long S. Lin X. (2023a). NK cell-derived exosomes enhance the anti-tumor effects against ovarian cancer by delivering cisplatin and reactivating NK cell functions. Front. Immunol. 13, 1087689. 10.3389/fimmu.2022.1087689 36741396 PMC9892755

[B226] Luo Y. Chen Y. Jin H. Hou B. Li H. Li X. (2023b). The suppression of cervical cancer ferroptosis by macrophages: the attenuation of ALOX15 in cancer cells by macrophages-derived exosomes. Acta Pharm. Sin. B 13, 2645–2662. 10.1016/j.apsb.2023.03.025 37425043 PMC10326300

[B227] Lv C. Yu W.-X. Wang Y. Yi D.-J. Zeng M.-H. Xiao H.-M. (2018). MiR-21 in extracellular vesicles contributes to the growth of fertilized eggs and embryo development in mice. Biosci. Rep. 38, BSR20180036. 10.1042/BSR20180036 29884767 PMC6117624

[B228] Lv C.-X. Duan H. Wang S. Gan L. Xu Q. J. R. S. (2020). Exosomes derived from human umbilical cord mesenchymal stem cells promote proliferation of allogeneic endometrial stromal cells. Reprod. Sci. 27, 1372–1381. 10.1007/s43032-020-00165-y 32006246

[B229] Ma J. Zhang J. Liu S. Gao S. XI H. Wang Z. (2024). Dual-drug-loaded MSCs-derived exosomal vesicles inhibit endometrial cancer cell proliferation by promoting apoptosis through the migration and invasion of Rac1/NF-κB/MMP2 signalling pathway. Biotechnol. Bioprocess Eng. 29, 551–563. 10.1007/s12257-024-00088-4

[B230] Maas S. L. N. de Vrij J. Van der Vlist E. J. Geragousian B. Van Bloois L. Mastrobattista E. (2015). Possibilities and limitations of current technologies for quantification of biological extracellular vesicles and synthetic mimics. J. Control. Release 200, 87–96. 10.1016/j.jconrel.2014.12.041 25555362 PMC4324667

[B231] Macdonald T. M. Walker S. P. Hannan N. J. Tong S. Tu'Uhevaha J. (2022). Clinical tools and biomarkers to predict preeclampsia. EBioMedicine 75, 103780. 10.1016/j.ebiom.2021.103780 34954654 PMC8718967

[B232] Machtinger R. Baccarelli A. A. Wu H. (2021). Extracellular vesicles and female reproduction. J. Assisted Reproduction Genet. 38, 549–557. 10.1007/s10815-020-02048-2 33471231 PMC7910356

[B233] Mansoori M. Solhjoo S. Palmerini M. G. Nematollahi-Mahani S. N. Ezzatabadipour M. (2024). Granulosa cell insight: unraveling the potential of menstrual blood-derived stem cells and their exosomes on mitochondrial mechanisms in polycystic ovary syndrome (PCOS). J. Ovarian Res. 17, 167. 10.1186/s13048-024-01484-3 39153978 PMC11330151

[B234] Mansouri-Kivaj N. Nazari A. Esfandiari F. Shekari F. Ghaffari M. Pakzad M. (2023). Homogenous subpopulation of human mesenchymal stem cells and their extracellular vesicles restore function of endometrium in an experimental rat model of Asherman syndrome. Stem Cell Res. and Ther. 14, 61. 10.1186/s13287-023-03279-7 37013655 PMC10071639

[B235] Mao Y. Liu P. Wei J. Xie Y. Zheng Q. Hu X. (2024). Exosomes derived from Umbilical cord mesenchymal stem cell promote hair regrowth in C57BL6 mice through upregulation of the RAS/ERK signaling pathway. J. Transl. Intern. Med. 12, 478–494. 10.1515/jtim-2024-0012 39513036 PMC11538887

[B236] Marchante M. Buigues A. Ramirez-Martin N. Martinez J. Pellicer N. Pellicer A. (2023). Single intraovarian dose of stem cell–and platelet-secreted factors mitigates age-related ovarian infertility in a murine model. GYNECOLOGY 228, 561.e1–561.e17. 10.1016/j.ajog.2023.01.018 36706857

[B237] Maroto R. Zhao Y. Jamaluddin M. Popov V. L. Wang H. Kalubowilage M. (2017). Effects of storage temperature on airway exosome integrity for diagnostic and functional analyses. J. Extracell. vesicles 6, 1359478. 10.1080/20013078.2017.1359478 28819550 PMC5556670

[B238] Martinez R. M. Liang L. Racowsky C. Dioni L. Mansur A. Adir M. (2018). Extracellular microRNAs profile in human follicular fluid and IVF outcomes. Sci. Rep. 8, 17036. 10.1038/s41598-018-35379-3 30451969 PMC6242846

[B239] Mashouri L. Yousefi H. Aref A. R. Ahadi A. M. Molaei F. Alahari S. K. (2019). Exosomes: composition, biogenesis, and mechanisms in cancer metastasis and drug resistance. Mol. cancer 18, 75. 10.1186/s12943-019-0991-5 30940145 PMC6444571

[B240] Mehravar M. Salimi M. Kazemi M. Majidi M. Roshandel E. Nazarian H. (2025). Utilization of MSC-Derived extracellular vesicles and bioscaffolds in enhancing oocyte *in vitro* maturation and culture: a review. Reprod. Sci., 1–13. 10.1007/s43032-025-01889-5 40715792

[B241] Menon U. Gentry-Maharaj A. Burnell M. Singh N. Ryan A. Karpinskyj C. (2021). Ovarian cancer population screening and mortality after long-term follow-up in the UK Collaborative Trial of Ovarian cancer screening (UKCTOCS): a randomised controlled trial. arXiv 397, 2182–2193. 10.1016/S0140-6736(21)00731-5 33991479 PMC8192829

[B242] Merivaara A. Zini J. Koivunotko E. Valkonen S. Korhonen O. Fernandes F. M. (2021). Preservation of biomaterials and cells by freeze-drying: change of paradigm. J. Control. Release 336, 480–498. 10.1016/j.jconrel.2021.06.042 34214597

[B243] Mincheva-Nilsson L. (2021). Immunosuppressive protein signatures carried by syncytiotrophoblast-derived exosomes and their role in human pregnancy. Front. Immunol. 12, 717884. 10.3389/fimmu.2021.717884 34381459 PMC8350734

[B244] Mincheva‐Nilsson L. Baranov V. (2014). Placenta‐derived exosomes and syncytiotrophoblast microparticles and their role in human reproduction: immune modulation for pregnancy success. Am. J. reproductive Immunol. 72, 440–457. 10.1111/aji.12311 25164206

[B245] Miron R. J. Zhang Y. (2024). Understanding exosomes: part 1—Characterization, quantification and isolation techniques. Periodontol. 2000 94, 231–256. 10.1111/prd.12520 37740431

[B246] Moghadasi S. Elveny M. Rahman H. S. Suksatan W. Jalil A. T. Abdelbasset W. K. (2021). A paradigm shift in cell-free approach: the emerging role of MSCs-derived exosomes in regenerative medicine. J. Transl. Med. 19, 302–321. 10.1186/s12967-021-02980-6 34253242 PMC8273572

[B247] Moghaddam M. Z. Ansariniya H. Seifati S. M. Zare F. Fesahat F. (2022). Immunopathogenesis of endometriosis: an overview of the role of innate and adaptive immune cells and their mediators. Am. J. Reprod. Immunol. 87, e13537. 10.1111/aji.13537 35263479

[B248] Montaner-Tarbes S. Novell E. Tarancón V. Borrás F. E. Montoya M. Fraile L. (2018). Targeted-pig trial on safety and immunogenicity of serum-derived extracellular vesicles enriched fractions obtained from porcine respiratory and reproductive virus infections. Sci. Rep. 8, 17487. 10.1038/s41598-018-36141-5 30504834 PMC6269534

[B249] Morales-Prieto D. M. Favaro R. R. Markert U. R. (2020). Placental miRNAs in feto-maternal communication mediated by extracellular vesicles. Placenta 102, 27–33. 10.1016/j.placenta.2020.07.001 33218575

[B250] Moss L. D. Sode D. Patel R. Lui A. Hudson C. Patel N. A. (2021). Intranasal delivery of exosomes from human adipose derived stem cells at forty-eight hours post injury reduces motor and cognitive impairments following traumatic brain injury. Neurochem. Int. 150, 105173. 10.1016/j.neuint.2021.105173 34453976 PMC8511339

[B251] Mu J. Zhuang X. Wang Q. Jiang H. Deng Z. B. Wang B. (2014). Interspecies communication between plant and mouse gut host cells through edible plant derived exosome‐like nanoparticles. Mol. Nutr. and food Res. 58, 1561–1573. 10.1002/mnfr.201300729 24842810 PMC4851829

[B252] Musante L. (2024). Urinary extracellular vesicles isolated by hydrostatic filtration dialysis without tamm–horsfall protein interference for mass spectrometry analysis. Proteomics Mass Spectrom. Methods, 101–116. 10.1016/b978-0-323-90395-0.00013-9

[B253] Musante L. Tataruch D. Gu D. Benito-Martin A. Calzaferri G. Aherne S. (2014). A simplified method to recover urinary vesicles for clinical applications and sample banking. Sci. Rep. 4, 7532. 10.1038/srep07532 25532487 PMC4274508

[B254] Nakase I. (2021). Biofunctional peptide-modified extracellular vesicles enable effective intracellular delivery *via* the induction of macropinocytosis. Processes 9, 224. 10.3390/pr9020224

[B255] Natali L. Luna Pizarro G. Moyano S. de La Cruz-Thea B. Musso J. Rópolo A. S. (2023). The exosome-like vesicles of giardia assemblages A, B, and E are involved in the delivering of distinct small RNA from parasite to parasite. Int. J. Mol. Sci. 24, 9559. 10.3390/ijms24119559 37298511 PMC10253879

[B256] Navarro C. Cabrera P. T. Garrán A. T. (2025). Comparative analysis of the use of autologous exosomes and platelet-derived growth factors in women with premature ovarian insufficiency and infertility: a prospective, randomized, observational, analytical study. Regen. Ther. 30, 309–320. 10.1016/j.reth.2025.06.007 40678344 PMC12268344

[B257] Nazdikbin Yamchi N. Ahmadian S. Mobarak H. Amjadi F. Beheshti R. Tamadon A. (2023). Amniotic fluid-derived exosomes attenuated fibrotic changes in POI rats through modulation of the TGF-β/Smads signaling pathway. J. Ovarian Res. 16, 118. 10.1186/s13048-023-01214-1 37370156 PMC10294370

[B258] Nelson L. M. (2009). Clinical practice. Primary ovarian insufficiency. N. Engl. J. Med. 360, 606–614. 10.1056/NEJMcp0808697 19196677 PMC2762081

[B259] Neto A. Botelho M. Rodrigues A. Lamas S. Araújo B. Guimarães J. (2024). Metformin reverses infertility in a mouse model of endometriosis: unveiling disease pathways and implications for future clinical approaches. Reprod. Biomed. Online 50, 104474. 10.1016/j.rbmo.2024.104474 39847839

[B260] Newton J. R. Mackenzie W. E. Emens M. J. Jordan J. A. (1989). Division of uterine adhesions (Asherman's syndrome) with the Nd‐YAG laser. BJOG An Int. J. Obstetrics and Gynaecol. 96, 102–104. 10.1111/j.1471-0528.1989.tb01584.x 2923832

[B261] Nouri N. Shareghi-Oskoue O. Aghebati-Maleki L. Danaii S. Ahmadian Heris J. Soltani-Zangbar M. S. (2022). Role of miRNAs interference on ovarian functions and premature ovarian failure. Cell Commun. Signal. 20, 198. 10.1186/s12964-022-00992-3 36564840 PMC9783981

[B262] Ogunnaike M. Wang H. Zempleni J. (2021). Bovine mammary alveolar MAC-T cells afford a tool for studies of bovine milk exosomes in drug delivery. Int. J. Pharm. 610, 121263. 10.1016/j.ijpharm.2021.121263 34742829 PMC8665143

[B263] Ou X. Wang H. Tie H. Liao J. Luo Y. Huang W. (2023). Novel plant-derived exosome-like nanovesicles from catharanthus roseus: preparation, characterization, and immunostimulatory effect *via* TNF-α/NF-κB/PU. 1 axis. J. nanobiotechnology 21, 160. 10.1186/s12951-023-01919-x 37210530 PMC10199296

[B264] O’Brien K. Breyne K. Ughetto S. Laurent L. C. Breakefield X. O. (2020). RNA delivery by extracellular vesicles in Mammalian cells and its applications. Nat. Rev. Mol. Cell Biol. 21, 585–606. 10.1038/s41580-020-0251-y 32457507 PMC7249041

[B265] Pak H. Hadizadeh A. Heirani‐Tabasi A. Soleimani M. Asbagh R. A. Fazeli M. S. (2023). Safety and efficacy of injection of human placenta mesenchymal stem cells derived exosomes for treatment of complex perianal fistula in non‐Crohn's cases: clinical trial phase I. J. Gastroenterology Hepatology 38, 539–547. 10.1111/jgh.16110 36640153

[B266] Palmulli R. Couty M. Piontek M. C. Ponnaiah M. Dingli F. Verweij F. J. (2024). CD63 sorts cholesterol into endosomes for storage and distribution *via* exosomes. Nat. Cell Biol. 26, 1093–1109. 10.1038/s41556-024-01432-9 38886558

[B267] Palviainen M. Saari H. Kärkkäinen O. Pekkinen J. Auriola S. Yliperttula M. (2019). Metabolic signature of extracellular vesicles depends on the cell culture conditions. J. Extracell. vesicles 8, 1596669. 10.1080/20013078.2019.1596669 31007875 PMC6461113

[B268] Pan Y. Wang X. Li Y. Yan P. Zhang H. (2022). Human umbilical cord blood mesenchymal stem cells-derived exosomal microRNA-503-3p inhibits progression of human endometrial cancer cells through downregulating MEST. Cancer Gene Ther. 29, 1130–1139. 10.1038/s41417-021-00416-3 34997218

[B269] Pan X. Shi X. Zhang H. Chen Y. Zhou J. Shen F. (2024a). Exosomal miR-4516 derived from ovarian cancer stem cells enhanced cisplatin tolerance in ovarian cancer by inhibiting GAS7. Gene 927, 148738. 10.1016/j.gene.2024.148738 38955306

[B270] Pan Y. Pan C. Zhang C. (2024b). Unraveling the complexity of follicular fluid: insights into its composition, function, and clinical implications. J. Ovarian Res. 17, 237. 10.1186/s13048-024-01551-9 39593094 PMC11590415

[B271] Park H.-S. Chugh R. M. Seok J. Cetin E. Mohammed H. Siblini H. (2023). Comparison of the therapeutic effects between stem cells and exosomes in primary ovarian insufficiency: as promising as cells but different persistency and dosage. Stem cell Res. and Ther. 14, 165. 10.1186/s13287-023-03397-2 37340468 PMC10283237

[B272] Park H.-S. Seok J. Cetin E. Ghasroldasht M. M. Ali F. L. Mohammed H. (2024). Fertility protection: a novel approach using pretreatment with mesenchymal stem cell exosomes to prevent chemotherapy–induced ovarian damage in a mouse model. Am. J. Obstetrics Gynecol. 231, 111-e1–111.e18. 10.1016/j.ajog.2024.02.023 38378099

[B273] Patel D. B. Gray K. M. Santharam Y. Lamichhane T. N. Stroka K. M. Jay S. M. (2017). Impact of cell culture parameters on production and vascularization bioactivity of mesenchymal stem cell‐derived extracellular vesicles. Bioeng. and Transl. Med. 2, 170–179. 10.1002/btm2.10065 28932818 PMC5579732

[B274] Patel S. K. Valicherla G. R. Micklo A. C. Rohan L. C. (2021). Drug delivery strategies for management of women’s health issues in the upper genital tract. Adv. Drug Deliv. Rev. 177, 113955. 10.1016/j.addr.2021.113955 34481034

[B275] Pei S. Sun W. Han Q. Wang H. Liang Q. (2024). Bifunctional immunoaffinity magnetic nanoparticles for high-efficiency separation of exosomes based on host-guest interaction. Talanta 272, 125790. 10.1016/j.talanta.2024.125790 38382302

[B276] Pillay P. Moodley K. Moodley J. Mackraj I. (2017). Placenta-derived exosomes: potential biomarkers of preeclampsia. Int. J. nanomedicine 12, 8009–8023. 10.2147/IJN.S142732 29184401 PMC5673050

[B277] Pisano S. Pierini I. Gu J. Gazze A. Francis L. W. Gonzalez D. (2020). Immune (cell) derived exosome mimetics (IDEM) as a treatment for ovarian cancer. Front. Cell Dev. Biol. 8, 553576. 10.3389/fcell.2020.553576 33042993 PMC7528637

[B278] Popowski K. D. Moatti A. Scull G. Silkstone D. Lutz H. de Juan Abad B. L. (2022). Inhalable dry powder mRNA vaccines based on extracellular vesicles. Matter 5, 2960–2974. 10.1016/j.matt.2022.06.012 35847197 PMC9272513

[B279] Pourakbari R. Ahmadi H. Yousefi M. Aghebati-Maleki L. (2020). Cell therapy in female infertility-related diseases: emphasis on recurrent miscarriage and repeated implantation failure. Life Sci. 258, 118181. 10.1016/j.lfs.2020.118181 32763291

[B280] Pu X. Zhang L. Zhang P. Xu Y. Wang J. Zhao X. (2023). Human UC-MSC-derived exosomes facilitate ovarian renovation in rats with chemotherapy-induced premature ovarian insufficiency. Front. Endocrinol. 14, 1205901. 10.3389/fendo.2023.1205901 37564988 PMC10411896

[B281] Puhka M. Nordberg M. E. Valkonen S. Rannikko A. Kallioniemi O. Siljander P. (2017). KeepEX, a simple dilution protocol for improving extracellular vesicle yields from urine. Eur. J. Pharm. Sci. 98, 30–39. 10.1016/j.ejps.2016.10.021 27771514

[B282] Qin J. Sun M. Cheng J. Jiang H. Lv M. Jing J. (2024). Ultrasound-responsive hydrogel incorporated with TGF-β mimetic peptides for endometrium recovery to restore fertility. ACS Appl. Mater. Interfaces 16, 57963–57971. 10.1021/acsami.4c07290 39415495

[B283] Qin Z. Yu Q. Long Y. (2025). Unveiling the pathological landscape of intrauterine adhesion: mechanistic insights and exosome-biomaterial therapeutic innovations. Int. J. Nanomedicine 20, 9667–9694. 10.2147/IJN.S527637 40785949 PMC12335249

[B284] Qiu L. Wang J. Chen M. Chen F. Tu W. (2020). Exosomal microRNA-146a derived from mesenchymal stem cells increases the sensitivity of ovarian cancer cells to docetaxel and taxane *via* a LAMC2-mediated PI3K/Akt axis. Int. J. Mol. Med. 46, 609–620. 10.3892/ijmm.2020.4634 32626953 PMC7307828

[B285] Qu Q. Liu L. Cui Y. Liu H. Yi J. Bing W. (2022). miR-126-3p containing exosomes derived from human umbilical cord mesenchymal stem cells promote angiogenesis and attenuate ovarian granulosa cell apoptosis in a preclinical rat model of premature ovarian failure. Stem Cell Res. Ther. 13, 352. 10.1186/s13287-022-03056-y 35883161 PMC9327169

[B286] Rashidi M. Bijari S. Khazaei A. H. Shojaei-Ghahrizjani F. Rezakhani L. (2022). The role of milk-derived exosomes in the treatment of diseases. Front. Genet. 13, 1009338. 10.3389/fgene.2022.1009338 36338966 PMC9634108

[B287] Regente M. Corti-Monzón G. Maldonado A. M. Pinedo M. Jorrín J. de La Canal L. (2009). Vesicular fractions of sunflower apoplastic fluids are associated with potential exosome marker proteins. FEBS Lett. 583, 3363–3366. 10.1016/j.febslet.2009.09.041 19796642

[B288] Reza A. M. M. T. Choi Y.-J. Yasuda H. Kim J.-H. (2016). Human adipose mesenchymal stem cell-derived exosomal-miRNAs are critical factors for inducing anti-proliferation signalling to A2780 and SKOV-3 ovarian cancer cells. Sci. Rep. 6, 38498. 10.1038/srep38498 27929108 PMC5143979

[B289] Rodolakis A. Scambia G. Planchamp F. Acien M. Di Spiezio Sardo A. Farrugia M. (2023). ESGO/ESHRE/ESGE guidelines for the fertility-sparing treatment of patients with endometrial carcinoma. Hum. Reprod. open 2023, hoac057. 10.1093/hropen/hoac057 36756380 PMC9900425

[B290] Rodríguez-Eguren A. Gómez-Álvarez M. Francés-Herrero E. Romeu M. Ferrero H. Seli E. (2022). Human umbilical cord-based therapeutics: stem cells and blood derivatives for female reproductive medicine. Int. J. Mol. Sci. 23, 15942. 10.3390/ijms232415942 36555583 PMC9785531

[B291] Rupert D. L. Claudio V. Lässer C. Bally M. (2017). Methods for the physical characterization and quantification of extracellular vesicles in biological samples. Biochim. Biophys. Acta. Gen. Subj. 1861, 3164–3179. 10.1016/j.bbagen.2016.07.028 27495390

[B292] Rutter B. D. Innes R. W. (2020). Growing pains: addressing the pitfalls of plant extracellular vesicle research. New Phytol. 228, 1505–1510. 10.1111/nph.16725 32506490

[B293] Safaei R. Larson B. J. Cheng T. C. Gibson M. A. Otani S. Naerdemann W. (2005). Abnormal lysosomal trafficking and enhanced exosomal export of cisplatin in drug-resistant human ovarian carcinoma cells. Mol. cancer Ther. 4, 1595–1604. 10.1158/1535-7163.MCT-05-0102 16227410

[B294] Sagae Y. Horie A. Yanai A. Ohara T. Nakakita B. Kitawaki Y. (2023). Versican provides the provisional matrix for uterine spiral artery dilation and fetal growth. Matrix Biol. 115, 16–31. 10.1016/j.matbio.2022.11.004 36423736

[B295] Sahebi R. Langari H. Fathinezhad Z. Bahari Sani Z. Avan A. Ghayour Mobarhan M. (2020). Exosomes: new insights into cancer mechanisms. J. Cell. Biochem. 121, 7–16. 10.1002/jcb.29120 31701565

[B296] Salehpour A. Karimi Z. Ghasemi Zadeh M. Afshar M. Kameli A. Mooseli F. (2024). Therapeutic potential of mesenchymal stem cell-derived exosomes and miRNAs in neuronal regeneration and rejuvenation in neurological disorders: a mini review. Front. Cell. Neurosci. 18, 1427525. 10.3389/fncel.2024.1427525 39429946 PMC11486650

[B297] Salomon C. Guanzon D. Scholz-Romero K. Longo S. Correa P. Illanes S. E. (2017). Placental exosomes as early biomarker of preeclampsia: potential role of exosomal microRNAs across gestation. J. Clin. Endocrinol. and Metabolism 102, 3182–3194. 10.1210/jc.2017-00672 28531338

[B298] Santos‐Coquillat A. González M. I. Clemente‐Moragón A. González‐Arjona M. Albaladejo‐García V. Peinado H. (2022). Goat milk exosomes as natural nanoparticles for detecting inflammatory processes by optical imaging. Small 18, 2105421. 10.1002/smll.202105421 34854563

[B299] Sanz-Ros J. Romero-García N. Mas-Bargues C. Monleón D. Gordevicius J. Brooke R. T. (2022). Small extracellular vesicles from young adipose-derived stem cells prevent frailty, improve health span, and decrease epigenetic age in old mice. arXiv 8, eabq2226. 36260670 10.1126/sciadv.abq2226 PMC9581480

[B300] Saribas G. S. Ozogul C. Tiryaki M. Pinarli F. A. Kilic S. H. (2020). Effects of uterus derived mesenchymal stem cells and their exosomes on asherman’s syndrome. Acta Histochem. 122, 151465. 10.1016/j.acthis.2019.151465 31776004

[B301] Sausset R. Krupova Z. Guédon E. Peron S. Grangier A. Petit M. A. (2023). Comparison of interferometric light microscopy with nanoparticle tracking analysis for the study of extracellular vesicles and bacteriophages. J. Extracell. Biol. 2, e75. 10.1002/jex2.75 38938523 PMC11080698

[B302] Schwich E. Rebmann V. Horn P. A. Celik A. A. Bade-Döding C. Kimmig R. (2019). Vesicular-bound HLA-G as a predictive marker for disease progression in epithelial ovarian cancer. Cancers 11, 1106. 10.3390/cancers11081106 31382533 PMC6721594

[B303] Sengupta V. Sengupta S. Lazo A. Woods P. Nolan A. Bremer N. (2020). Exosomes derived from bone marrow mesenchymal stem cells as treatment for severe COVID-19. Stem cells Dev. 29, 747–754. 10.1089/scd.2020.0080 32380908 PMC7310206

[B304] Shaoyong W. Liu Y. Xu B. Pan B. Xianmi X. Wang Y. (2022). Exposure to BDE-47 causes female infertility risk and induces oxidative stress and lipotoxicity-mediated ovarian hormone secretion disruption in mice. Sci. Total Environ. 842, 156885. 10.1016/j.scitotenv.2022.156885 35752246

[B305] Sharma P. Dhamija R. K. Nag T. C. Roy A. Inampudi K. K. (2023). Different biofluids, small extracellular vesicles or exosomes: structural analysis in atherosclerotic cardiovascular disease using electron microscopy techniques. Microsc. Microanal. 29, 1168–1177. 10.1093/micmic/ozad025 37749667

[B306] Sheller-Miller S. Lei J. Saade G. Salomon C. Burd I. Menon R. (2016). Feto-maternal trafficking of exosomes in murine pregnancy models. Front. Pharmacol. 7, 432. 10.3389/fphar.2016.00432 27895585 PMC5108780

[B307] Shelley C. E. Otter S. J. Stewart A. J. Stewart A. J. (2021). Adaptive radiotherapy in the management of cervical cancer: review of strategies and clinical implementation. Clin. Oncol. 33, 579–590. 10.1016/j.clon.2021.06.007 34247890

[B308] Shen K.-Y. Dai X.-L. Li S. Huang F. Chen L.-Q. Luo P. (2023). Specific expression profile of follicular fluid-derived exosomal microRNAs in patients with diminished ovarian reserve. BMC Med. Genomics 16, 308. 10.1186/s12920-023-01756-9 38037065 PMC10688486

[B309] Sherer M. V. Kotha N. V. Williamson C. Mayadev J. (2022). Advances in immunotherapy for cervical cancer: recent developments and future directions. Int. J. Gynecol. Cancer 32, 281–287. 10.1136/ijgc-2021-002492 35256414

[B310] Shetgaonkar G. G. Marques S. M. Dcruz C. E. Vibhavari R. Kumar L. Shirodkar R. K. (2022). Exosomes as cell-derivative carriers in the diagnosis and treatment of central nervous system diseases. Drug Deliv. Transl. Res. 12, 1047–1079. 10.1007/s13346-021-01026-0 34365576 PMC8942947

[B311] Shi S. Tan Q. Feng F. Huang H. Liang J. Cao D. (2020). Identification of core genes in the progression of endometrial cancer and cancer cell-derived exosomes by an integrative analysis. Sci. Rep. 10, 9862. 10.1038/s41598-020-66872-3 32555395 PMC7299953

[B312] Shi W. Liu Y. Qiu X. Yang L. Lin G. (2023). Cancer-associated fibroblasts-derived exosome-mediated transfer of miR-345-5p promotes the progression of colorectal cancer by targeting CDKN1A. Carcinogenesis 44, 317–327. 10.1093/carcin/bgad014 37052230

[B313] Shi M. Chen Z. Gong H. Peng Z. Sun Q. Luo K. (2024). Luteolin, a flavone ingredient: anticancer mechanisms, combined medication strategy, pharmacokinetics, clinical trials, and pharmaceutical researches. Phytotherapy Res. 38, 880–911. 10.1002/ptr.8066 38088265

[B314] Shirejini S. Z. Inci F. (2022). The yin and yang of exosome isolation methods: conventional practice, microfluidics, and commercial kits. Biotechnol. Adv. 54, 107814. 10.1016/j.biotechadv.2021.107814 34389465

[B315] Shu S. L. Allen C. L. Benjamin-Davalos S. Koroleva M. Macfarland D. Minderman H. (2021). A rapid exosome isolation using ultrafiltration and size exclusion chromatography (REIUS) method for exosome isolation from melanoma cell lines. Melanoma methods Protoc. 2265, 289–304. 10.1007/978-1-0716-1205-7_22 33704723 PMC8865195

[B316] Si K. Dai Z. Li Z. Ye Z. Ding B. Feng S. (2023). Engineered exosome-mediated messenger RNA and single-chain variable fragment delivery for human chimeric antigen receptor T-cell engineering. Cytotherapy 25, 615–624. 10.1016/j.jcyt.2023.01.005 36828738

[B317] Siddiqui S. Riaz S. Ahmad R. Wani M. J. Hashmi M. A. Zofair S. F. F. (2023). Synergistic effect of chlorogenic acid and vitamin D3 (cholecalciferol) on *in-vitro* glycation May assist in prevention of polycystic ovarian syndrome (PCOS) progression-A biophysical, biochemical and *in-silico* study. Int. J. Biol. Macromol. 245, 125497. 10.1016/j.ijbiomac.2023.125497 37369258

[B318] Singh A. Behl T. Sehgal A. Singh S. Sharma N. Naqwi M. (2023). Exploring the role of exosomes in rheumatoid arthritis. Inflammopharmacology 31, 119–128. 10.1007/s10787-022-01100-0 36414831

[B319] Smyth T. Petrova K. Payton N. M. Persaud I. Redzic J. S. Graner M. W. (2014). Surface functionalization of exosomes using click chemistry. Bioconjugate Chem. 25, 1777–1784. 10.1021/bc500291r 25220352 PMC4198107

[B320] Smyth T. Kullberg M. Malik N. Smith-Jones P. Graner M. W. Anchordoquy T. J. (2015). Biodistribution and delivery efficiency of unmodified tumor-derived exosomes. J. Control. Release 199, 145–155. 10.1016/j.jconrel.2014.12.013 25523519 PMC4441346

[B321] Song C. Yao J. Cao C. Liang X. Huang J. Han Z. (2016). PPARγ is regulated by miR-27b-3p negatively and plays an important role in porcine oocyte maturation. Biochem. Biophysical Res. Commun. 479, 224–230. 10.1016/j.bbrc.2016.09.046 27638309

[B322] Song Z. Mao J. Barrero R. A. Wang P. Zhang F. Wang T. (2020). Development of a CD63 aptamer for efficient cancer immunochemistry and immunoaffinity-based exosome isolation. Molecules 25, 5585. 10.3390/molecules25235585 33261145 PMC7730289

[B323] Song Y. Wang M. Tong H. Tan Y. Hu X. Wang K. (2021). Plasma exosomes from endometrial cancer patients contain LGALS3BP to promote endometrial cancer progression. Oncogene 40, 633–646. 10.1038/s41388-020-01555-x 33208911

[B324] Song Y. Ye L. Tan Y. Tong H. Lv Z. Wan X. (2022). Therapeutic exosomes loaded with SERPINA5 attenuated endometrial cancer cell migration *via* the integrin β1/FAK signaling pathway. Cell. Oncol. 45, 861–872. 10.1007/s13402-022-00687-4 35951287 PMC12978094

[B325] Song A. Zhang S. Zhao X. Wu S. Qi X. Gao S. (2023). Exosomes derived from menstrual blood stromal cells ameliorated premature ovarian insufficiency and granulosa cell apoptosis by regulating SMAD3/AKT/MDM2/P53 pathway *via* delivery of thrombospondin-1. arXiv 166, 115319. 10.1016/j.biopha.2023.115319 37573658

[B326] Stanly C. Fiume I. Capasso G. Pocsfalvi G. (2016). Isolation of exosome-like vesicles from plants by ultracentrifugation on sucrose/deuterium oxide (D 2 O) density cushions. Methods Mol. Biol. 1459, 259–269. 10.1007/978-1-4939-3804-9_18 27665565

[B327] Staub E. Cao Q. Chen X.-M. Pollock C. (2025). Concentration of kidney markers and detection of exosomes in urine samples collected in cotton wool balls in preterm and term neonates. Pathology 57, 81–86. 10.1016/j.pathol.2024.07.006 39516170

[B328] Sun H. Li D. Yuan M. Li Q. Zhen Q. Li N. (2019). Macrophages alternatively activated by endometriosis-exosomes contribute to the development of lesions in mice. Mol. Hum. Reprod. 25, 5–16. 10.1093/molehr/gay049 30428082

[B329] Sun P. Zhang Y. Sun L. Sun N. Wang J. Ma H. (2023). Kisspeptin regulates the proliferation and apoptosis of ovary granulosa cells in polycystic ovary syndrome by modulating the PI3K/AKT/ERK signalling pathway. BMC Women's Health 23, 15. 10.1186/s12905-022-02154-6 36627631 PMC9832680

[B330] Sun P. Yu C. Yin L. Chen Y. Sun Z. Zhang T. (2024). Global, regional, and national burden of female cancers in women of child-bearing age, 1990–2021: analysis of data from the global burden of disease study 2021. EClinicalMedicine 74, 102713. 10.1016/j.eclinm.2024.102713 39050105 PMC11268131

[B331] Sung H. Ferlay J. Siegel R. L. Laversanne M. Soerjomataram I. Jemal A. (2021). Global cancer statistics 2020: GLOBOCAN estimates of incidence and mortality worldwide for 36 cancers in 185 countries. CA a cancer J. Clin. 71, 209–249. 10.3322/caac.21660 33538338

[B332] Suresh A. P. Kalarikkal S. P. Pullareddy B. Sundaram G. M. (2021). Low pH-based method to increase the yield of plant-derived nanoparticles from fresh ginger rhizomes. ACS omega 6, 17635–17641. 10.1021/acsomega.1c02162 34278148 PMC8280662

[B333] Takagi M. Jimbo S. Oda T. Goto Y. Fujiwara M. (2021). Polymer fraction including exosomes derived from Chinese hamster ovary cells promoted their growth during serum-free repeated batch culture. J. Biosci. Bioeng. 131, 183–189. 10.1016/j.jbiosc.2020.09.011 33051156

[B334] Tan Q. Xia D. Ying X. (2020). miR-29a in exosomes from bone marrow mesenchymal stem cells inhibit fibrosis during endometrial repair of intrauterine adhesion. Int. J. Stem Cells 13, 414–423. 10.15283/ijsc20049 33250449 PMC7691861

[B335] Tan J. Deng M. Xia M. Lai M. Pan W. Li Y. (2021). Comparison of hysterosalpingography with laparoscopy in the diagnosis of tubal factor of female infertility. Front. Med. 8, 720401. 10.3389/fmed.2021.720401 34778286 PMC8585930

[B336] Tanaka Y. Takahashi A. (2021). Senescence-associated extracellular vesicle release plays a role in senescence-associated secretory phenotype (SASP) in age-associated diseases. J. Biochem. 169, 147–153. 10.1093/jb/mvaa109 33002139

[B337] Tang H. He Y. Li L. Mao W. Chen X. Ni H. (2019). Exosomal MMP2 derived from mature osteoblasts promotes angiogenesis of endothelial cells *via* VEGF/Erk1/2 signaling pathway. Exp. cell Res. 383, 111541. 10.1016/j.yexcr.2019.111541 31369752

[B338] Tantengco O. A. G. Radnaa E. Shahin H. Kechichian T. Menon R. (2021). Cross talk: trafficking and functional impact of maternal exosomes at the feto-maternal interface under normal and pathologic states. Biol. reproduction 105, 1562–1576. 10.1093/biolre/ioab181 34554204

[B339] Tarafdari A. Vahdani F. G. Hadizadeh A. Khoshravesh S. Hadizadeh S. (2023). Spontaneous pregnancy in a case with concurrent uterine artery embolization induced transient premature ovarian failure and asherman syndrome: a case report. Oxf. Med. Case Rep. 2023, omad056. 10.1093/omcr/omad056 37377715 PMC10292642

[B340] Taravat M. Asadpour R. Jafari Jozani R. Fattahi A. Khordadmehr M. Hajipour H. (2024). Engineered exosome as a biological nanoplatform for drug delivery of rosmarinic acid to improve implantation in mice with induced endometritis. Syst. Biol. Reproductive Med. 70, 3–19. 10.1080/19396368.2024.2306420 38323586

[B341] Taylor D. D. Shah S. (2015). Methods of isolating extracellular vesicles impact down-stream analyses of their cargoes. Methods 87, 3–10. 10.1016/j.ymeth.2015.02.019 25766927

[B342] Taymour N. Haque M. A. Atia G. A. N. Mohamed S. Z. Rokaya D. Bajunaid S. M. (2024). Nanodiamond: a promising carbon‐based nanomaterial for therapeutic and regenerative dental applications. ChemistrySelect 9, e202401328. 10.1002/slct.202401328

[B343] Teng X. Wang Z. Wang X. (2024). Enhancing angiogenesis and inhibiting apoptosis: evaluating the therapeutic efficacy of bone marrow mesenchymal stem cell-derived exosomes in a DHEA-Induced PCOS mouse model. J. Ovarian Res. 17, 121. 10.1186/s13048-024-01445-w 38840218 PMC11151599

[B344] Tesarik J. Galán-Lázaro M. Mendoza-Tesarik R. (2021). Ovarian aging: molecular mechanisms and medical management. arXiv 22, 1371. 33573050 10.3390/ijms22031371 PMC7866420

[B345] Tian M.-Y. Hao D.-X. Liu Y. He J. Zhao Z.-H. Guo T.-Y. (2023). Milk exosomes: an oral drug delivery system with great application potential. Food Funct. 14, 1320–1337. 10.1039/d2fo02013k 36722924

[B346] Todeschini P. Salviato E. Paracchini L. Ferracin M. Petrillo M. Zanotti L. (2017). Circulating miRNA landscape identifies miR-1246 as promising diagnostic biomarker in high-grade serous ovarian carcinoma: a validation across two independent cohorts. Cancer Lett. 388, 320–327. 10.1016/j.canlet.2016.12.017 28017893

[B347] Tong M. Kleffmann T. Pradhan S. Johansson C. L. Desousa J. Stone P. R. (2016). Proteomic characterization of macro-micro-and nano-extracellular vesicles derived from the same first trimester placenta: relevance for feto-maternal communication. Hum. Reprod. 31, 687–699. 10.1093/humrep/dew004 26839151

[B348] Tong X. Xu Y. Zhang T. Deng C. Xun J. Sun D. (2023). Exosomes from CD133+ human urine-derived stem cells combined adhesive hydrogel facilitate rotator cuff healing by mediating bone marrow mesenchymal stem cells. J. Orthop. Transl. 39, 100–112. 10.1016/j.jot.2023.02.002 36879794 PMC9984782

[B349] Tsiampa E. Tsiampas K. Kapogiannis F. (2024). Perioperative and reproductive outcomes’ comparison of mini-laparotomy and laparoscopic myomectomy in the management of uterine leiomyomas: a systematic review. Archives Gynecol. obstetrics 309, 821–829. 10.1007/s00404-023-07168-5 37566224

[B350] Tu J. Luo X. Liu H. Zhang J. He M. (2021). Cancer spheroids derived exosomes reveal more molecular features relevant to progressed cancer. Biochem. biophysics Rep. 26, 101026. 10.1016/j.bbrep.2021.101026 34095553 PMC8167213

[B351] Tuscharoenporn T. Apaijai N. Charoenkwan K. Chattipakorn N. Chattipakorn S. C. (2025). Emerging roles of exosomes in diagnosis, prognosis, and therapeutic potential in ovarian cancer: a comprehensive review. Cancer Gene Ther. 32, 149–164. 10.1038/s41417-025-00871-2 39843770

[B352] Umair Z. Baek M.-O. Song J. An S. Chon S. J. Yoon M.-S. (2022). MicroRNA-4516 in urinary exosomes as a biomarker of premature ovarian insufficiency. Cells 11, 2797. 10.3390/cells11182797 36139370 PMC9497098

[B353] Vaiciuleviciute R. Brennan K. Uzieliene I. Pachaleva J. Kasilovskiene Z. Piesiniene L. (2025). Proteomic signature of menstrual blood mesenchymal stromal cells and their extracellular vesicles in women with unexplained infertility. Reprod. Biomed. Online 51, 104980. 10.1016/j.rbmo.2025.104980 40845786

[B354] Van de Wakker S. I. Van Oudheusden J. Mol E. A. Roefs M. T. Zheng W. Görgens A. (2022). Influence of short term storage conditions, concentration methods and excipients on extracellular vesicle recovery and function. Eur. J. Pharm. Biopharm. 170, 59–69. 10.1016/j.ejpb.2021.11.012 34864197

[B355] Van der Pol E. Coumans F. A. W. Grootemaat A. E. Harrison P. Sturk A. Van Leeuwen T. G. (2014). Particle size distribution of exosomes and microvesicles determined by transmission electron microscopy, flow cytometry, nanoparticle tracking analysis, and resistive pulse sensing. J. Thrombosis Haemostasis 12, 1182–1192. 10.1111/jth.12602 24818656

[B356] Van Deun J. Mestdagh P. Sormunen R. Cocquyt V. Vermaelen K. Vandesompele J. (2014). The impact of disparate isolation methods for extracellular vesicles on downstream RNA profiling. J. Extracell. vesicles 3, 24858. 10.3402/jev.v3.24858 25317274 PMC4169610

[B357] Verlohren S. Dröge L.-A. (2022). The diagnostic value of angiogenic and antiangiogenic factors in differential diagnosis of preeclampsia. Am. J. obstetrics Gynecol. 226, S1048–S1058. 10.1016/j.ajog.2020.09.046 33002498

[B358] Vilella F. Moreno-Moya J. M. Balaguer N. Grasso A. Herrero M. Martínez S. (2015). Hsa-miR-30d, secreted by the human endometrium, is taken up by the pre-implantation embryo and might modify its transcriptome. Development 142, 3210–3221. 10.1242/dev.124289 26395145

[B359] Wan J. He Z. Peng R. Wu X. Zhu Z. Cui J. (2023). Injectable photocrosslinking spherical hydrogel-encapsulated targeting peptide-modified engineered exosomes for osteoarthritis therapy. J. Nanobiotechnology 21, 284. 10.1186/s12951-023-02050-7 37605203 PMC10440922

[B361] Wang Y. Wang K. Hu Z. Zhou H. Zhang L. Wang H. (2018). MicroRNA-139-3p regulates osteoblast differentiation and apoptosis by targeting ELK1 and interacting with long noncoding RNA ODSM. Cell Death and Dis. 9, 1107. 10.1038/s41419-018-1153-1 30382082 PMC6208413

[B362] Wang J. Jayaprakasha G. K. Patil B. S. (2020). Untargeted chemometrics evaluation of the effect of juicing technique on phytochemical profiles and antioxidant activities in common vegetables. ACS Food Sci. and Technol. 1, 77–87. 10.1021/acsfoodscitech.0c00013

[B363] Wang Y.-T. Cai M.-D. Sun L.-L. Hua R.-N. (2021). A rapid and facile separation–detection integrated strategy for exosome profiling based on boronic acid-directed coupling immunoaffinity. Anal. Chem. 93, 16059–16067. 10.1021/acs.analchem.1c03643 34793122

[B364] Wang L. Cheng W. Zhu J. Li W. Li D. Yang X. (2022a). Electrospun nanoyarn and exosomes of adipose-derived stem cells for urethral regeneration: evaluations *in vitro* and *in vivo* . Colloids Surfaces B Biointerfaces 209, 112218. 10.1016/j.colsurfb.2021.112218 34801930

[B365] Wang X. Zhao X. Zhong Y. Shen J. An W. (2022b). Biomimetic exosomes: a new generation of drug delivery system. Front. Bioeng. Biotechnol. 10, 865682. 10.3389/fbioe.2022.865682 35677298 PMC9168598

[B366] Wang Y. Zhou C. Meng F. Hu Q. Ding Y. Wang X. (2022c). Ssc-miR-92b-3p regulates porcine trophoblast cell proliferation and migration *via* the PFKM gene. Int. J. Mol. Sci. 23, 16138. 10.3390/ijms232416138 36555776 PMC9784024

[B367] Wang S. Liu T. Nan N. Lu C. Liang M. Wang S. (2023). Exosomes from human umbilical cord mesenchymal stem cells facilitates injured endometrial restoring in early repair period through miR-202-3p mediating formation of ECM. Stem Cell Rev. Rep. 19, 1954–1964. 10.1007/s12015-023-10549-7 37226011

[B368] Wang L. Wang L. Wang R. Xu T. Wang J. Cui Z. (2024a). Endometrial stem cell-derived exosomes repair cisplatin-induced premature ovarian failure *via* hippo signaling pathway. Heliyon 10, e31639. 10.1016/j.heliyon.2024.e31639 38831834 PMC11145543

[B369] Wang W. Dong Y. Wang K. Sun H. Yu H. Ling B. (2024b). Dietary inflammatory index and female infertility: findings from NHANES survey. Front. Nutr. 11, 1391983. 10.3389/fnut.2024.1391983 39364152 PMC11446885

[B370] Wang W. Ren Y. Yu Q. Jiang L. Yu C. Yue Z. (2024c). Biodegradable exosome-engineered hydrogels for the prevention of peritoneal adhesions *via* anti-oxidation and anti-inflammation. Mater. Today Bio 29, 101312. 10.1016/j.mtbio.2024.101312 39525394 PMC11550211

[B371] Wang X. Zhang Z. Qi Y. Zhang Z. Zhang Y. Meng K. (2024d). Study of the uptake mechanism of two small extracellular vesicle subtypes by granulosa cells. Animal Reproduction Sci. 270, 107576. 10.1016/j.anireprosci.2024.107576 39178587

[B372] Wang C. Cheng F. Han Z. Yan B. Liao P. Yin Z. (2025). Human-induced pluripotent stem cell–derived neural stem cell exosomes improve blood–brain barrier function after intracerebral hemorrhage by activating astrocytes *via* PI3K/AKT/MCP-1 axis. Neural Regen. Res. 20, 518–532. 10.4103/NRR.NRR-D-23-01889 38819064 PMC11317932

[B373] Willms E. Johansson H. J. Mäger I. Lee Y. Blomberg K. E. M. Sadik M. (2016). Cells release subpopulations of exosomes with distinct molecular and biological properties. Sci. Rep. 6, 22519. 10.1038/srep22519 26931825 PMC4773763

[B374] Wu D. Lu P. Mi X. Miao J. (2018). Exosomal miR-214 from endometrial stromal cells inhibits endometriosis fibrosis. MHR Basic Sci. reproductive Med. 24, 357–365. 10.1093/molehr/gay019 29660008

[B375] Wu J.-Y. Li Y.-J. Hu X.-B. Huang S. Xiang D.-X. (2021a). Preservation of small extracellular vesicles for functional analysis and therapeutic applications: a comparative evaluation of storage conditions. Drug Deliv. 28, 162–170. 10.1080/10717544.2020.1869866 33427518 PMC7808382

[B376] Wu M. Chen Z. Xie Q. Xiao B. Zhou G. Chen G. (2021b). One-step quantification of salivary exosomes based on combined aptamer recognition and quantum dot signal amplification. Biosens. Bioelectron. 171, 112733. 10.1016/j.bios.2020.112733 33096430

[B377] Wu Y. Gu S. Cobb J. M. Dunn G. H. Muth T. A. Simchick C. J. (2024). E2-Loaded microcapsules and bone marrow–derived mesenchymal stem cells with injectable scaffolds for endometrial regeneration application. Tissue Eng. Part A 30, 115–130. 10.1089/ten.TEA.2023.0238 37930721

[B378] Xia C. Zeng Z. Fang B. Tao M. Gu C. Zheng L. (2019). Mesenchymal stem cell-derived exosomes ameliorate intervertebral disc degeneration *via* anti-oxidant and anti-inflammatory effects. Free Radic. Biol. Med. 143, 1–15. 10.1016/j.freeradbiomed.2019.07.026 31351174

[B379] Xian P. Hei Y. Wang R. Wang T. Yang J. Li J. (2019). Mesenchymal stem cell-derived exosomes as a nanotherapeutic agent for amelioration of inflammation-induced astrocyte alterations in mice. Theranostics 9, 5956–5975. 10.7150/thno.33872 31534531 PMC6735367

[B380] Xiang X. Chen J. Jiang T. Yan C. Kang Y. Zhang M. (2023). Milk-derived exosomes carrying siRNA-KEAP1 promote diabetic wound healing by improving oxidative stress. Drug Deliv. Transl. Res. 13, 2286–2296. 10.1007/s13346-023-01306-x 36749479 PMC9904251

[B381] Xie J. Yang Y. Zhuo A. Gao M. Tang L. Xiao Y. (2024). Exosomes derived from mesenchymal stem cells attenuate NLRP3-related pyroptosis in autoimmune premature ovarian insufficiency *via* the NF-κB pathway. Reprod. Biomed. Online 48, 103814. 10.1016/j.rbmo.2024.103814 38569224

[B382] Xin L. Lin X. Zhou F. Li C. Wang X. Yu H. (2020). A scaffold laden with mesenchymal stem cell-derived exosomes for promoting endometrium regeneration and fertility restoration through macrophage immunomodulation. Acta biomater. 113, 252–266. 10.1016/j.actbio.2020.06.029 32574858

[B383] Xiong Y. Xiong Y. Zhang H. Zhao Y. Han K. Zhang J. (2021). hPMSCs-derived exosomal miRNA-21 protects against aging-related oxidative damage of CD4+ T cells by targeting the PTEN/PI3K-Nrf2 axis. Front. Immunol. 12, 780897. 10.3389/fimmu.2021.780897 34887868 PMC8649962

[B384] Xiong J. Fan Y. Wang Y. Luo J. Chen T. Sun J. (2023). New signaling kid on the block in the endocrine system: the role of extracellular vesicles. Endocrinology 164, bqad099. 10.1210/endocr/bqad099 37378492

[B385] Xu Y.-W. Wang B. Ding C.-H. Li T. Gu F. Zhou C. (2011). Differentially expressed micoRNAs in human oocytes. J. assisted reproduction Genet. 28, 559–566. 10.1007/s10815-011-9590-0 21647640 PMC3158253

[B386] Xu M. Feng T. Liu B. Qiu F. Xu Y. Zhao Y. (2021). Engineered exosomes: desirable target-tracking characteristics for cerebrovascular and neurodegenerative disease therapies. Theranostics 11, 8926–8944. 10.7150/thno.62330 34522219 PMC8419041

[B387] Xu H. Li H. Zhang P. Gao Y. Ma H. Gao T. (2024). The functions of exosomes targeting astrocytes and astrocyte-derived exosomes targeting other cell types. Neural Regen. Res. 19, 1947–1953. 10.4103/1673-5374.390961 38227520 PMC11040311

[B388] Yamada T. Inoshima Y. Matsuda T. Ishiguro N. (2012). Comparison of methods for isolating exosomes from bovine milk. J. Veterinary Med. Sci. 74, 1523–1525. 10.1292/jvms.12-0032 22785357

[B389] Yamashita T. Takahashi Y. Nishikawa M. Takakura Y. (2016). Effect of exosome isolation methods on physicochemical properties of exosomes and clearance of exosomes from the blood circulation. Eur. J. Pharm. Biopharm. 98, 1–8. 10.1016/j.ejpb.2015.10.017 26545617

[B390] Yan Y. Jiang W. Tan Y. Zou S. Zhang H. Mao F. (2017). hucMSC exosome-derived GPX1 is required for the recovery of hepatic oxidant injury. Mol. Ther. 25, 465–479. 10.1016/j.ymthe.2016.11.019 28089078 PMC5368592

[B391] Yan F. Zhao Q. Li Y. Zheng Z. Kong X. Shu C. (2022a). The role of oxidative stress in ovarian aging: a review. J. Ovarian Res. 15, 100. 10.1186/s13048-022-01032-x 36050696 PMC9434839

[B392] Yan X. Zhang S. Jia J. Yang J. Song Y. Duan H. (2022b). Exosomal MiR-423–3p inhibits macrophage M2 polarization to suppress the malignant progression of cervical cancer. Pathology-Research Pract. 235, 153882. 10.1016/j.prp.2022.153882 35609397

[B393] Yan L. Lin P. Wu Z. Lu Z. Ma L. Dong X. (2023). Exosomal miRNA analysis provides new insights into exposure to nanoplastics and okadaic acid. Sci. Total Environ. 905, 167010. 10.1016/j.scitotenv.2023.167010 37722421

[B394] Yáñez-Mó M. Siljander P. R. M. Andreu Z. Bedina Zavec A. Borràs F. E. Buzas E. I. (2015). Biological properties of extracellular vesicles and their physiological functions. J. Extracell. vesicles 4, 27066. 10.3402/jev.v4.27066 25979354 PMC4433489

[B395] Yang M. Lin L. Sha C. Li T. Zhao D. Wei H. (2020a). Bone marrow mesenchymal stem cell-derived exosomal miR-144-5p improves rat ovarian function after chemotherapy-induced ovarian failure by targeting PTEN. Lab. Invest. 100, 342–352. 10.1038/s41374-019-0321-y 31537899

[B396] Yang W. Zhang J. Xu B. He Y. Liu W. Li J. (2020b). HucMSC-derived exosomes mitigate the age-related retardation of fertility in female mice. Mol. Ther. 28, 1200–1213. 10.1016/j.ymthe.2020.02.003 32097602 PMC7132622

[B397] Yang G. Yao G. Xu Z. Fan H. Liu X. He J. (2021a). Expression level of ADAMTS1 in granulosa cells of PCOS patients is related to granulosa cell function, oocyte quality, and embryo development. Front. cell Dev. Biol. 9, 647522. 10.3389/fcell.2021.647522 33912563 PMC8075003

[B398] Yang Z. Shan N. Deng Q. Wang Y. Hou Y. Mei J. (2021b). Extracellular vesicle‐derived microRNA‐18b ameliorates preeclampsia by enhancing trophoblast proliferation and migration *via* Notch2/TIM3/mTORC1 axis. J. Cell. Mol. Med. 25, 4583–4595. 10.1111/jcmm.16234 33835684 PMC8107107

[B399] Yang X. Peng Y. Wang Y.-E. Zheng Y. He Y. Pan J. (2023). Curcumae rhizoma Exosomes-like nanoparticles loaded astragalus components improve the absorption and enhance anti-tumor effect. J. Drug Deliv. Sci. Technol. 81, 104274. 10.1016/j.jddst.2023.104274

[B400] Yang J. Chen J. Zhang L. Zhou F. Cui X. Tian R. (2024a). Plasma proteome profiling reveals biomarkers of chemotherapy resistance in patients with advanced colorectal cancer. Quant. Biol. 12, 215–224. 10.1002/qub2.34 PMC12806364 41676369

[B401] Yang Y. Tang L. Xiao Y. Huang W. Gao M. Xie J. (2024b). miR-21-5p-loaded bone mesenchymal stem cell-derived exosomes repair ovarian function in autoimmune premature ovarian insufficiency by targeting MSX1. Reprod. Biomed. Online 48, 103815. 10.1016/j.rbmo.2024.103815 38582043

[B402] Yao J. Zheng J. Cai J. Zeng K. Zhou C. Zhang J. (2019a). Extracellular vesicles derived from human umbilical cord mesenchymal stem cells alleviate rat hepatic ischemia‐reperfusion injury by suppressing oxidative stress and neutrophil inflammatory response. FASEB J. 33, 1695–1710. 10.1096/fj.201800131RR 30226809

[B403] Yao Y. Chen R. Wang G. Zhang Y. Liu F. (2019b). Exosomes derived from mesenchymal stem cells reverse EMT *via* TGF-β1/Smad pathway and promote repair of damaged endometrium. Stem Cell Res. Ther. 10, 225–17. 10.1186/s13287-019-1332-8 31358049 PMC6664513

[B404] Ye S.-B. Li Z.-L. Luo D.-H. Huang B.-J. Chen Y.-S. Zhang X.-S. (2014). Tumor-derived exosomes promote tumor progression and T-cell dysfunction through the regulation of enriched exosomal microRNAs in human nasopharyngeal carcinoma. Oncotarget 5, 5439–5452. 10.18632/oncotarget.2118 24978137 PMC4170615

[B405] Ye Z. Wang S. Huang X. Chen P. Deng L. Li S. (2022). Plasma exosomal miRNAs associated with metabolism as early predictor of gestational diabetes mellitus. Diabetes 71, 2272–2283. 10.2337/db21-0909 35926094 PMC9630082

[B406] Yin Y. Cao S. Fu H. Fan X. Xiong J. Huang Q. (2020). A noncanonical role of NOD-Like receptor NLRP14 in PGCLC differentiation and spermatogenesis. Proc. Natl. Acad. Sci. U. S. A. 117, 22237–22248. 10.1073/pnas.2005533117 32839316 PMC7486727

[B407] Yu X. Odenthal M. Fries J. W. U. (2016). Exosomes as miRNA carriers: Formation–Function–Future. Int. J. Mol. Sci. 17, 2028. 10.3390/ijms17122028 27918449 PMC5187828

[B408] Yu L. Liu M. Wang Z. Liu T. Liu S. Wang B. (2021). Correlation between steroid levels in follicular fluid and hormone synthesis related substances in its exosomes and embryo quality in patients with polycystic ovary syndrome. Reproductive Biol. Endocrinol. 19, 74. 10.1186/s12958-021-00749-6 34001150 PMC8127216

[B409] Yu X. Wu H. Yang Y. Wang F. Wang Y.-L. Shao X. (2022). Placental development and pregnancy-associated diseases. Maternal-Fetal Med. 4, 36–51. 10.1097/FM9.0000000000000134 40406576 PMC12094368

[B410] Yuana Y. Böing A. N. Grootemaat A. E. Van der Pol E. Hau C. M. Cizmar P. (2015). Handling and storage of human body fluids for analysis of extracellular vesicles. J. Extracell. vesicles 4, 29260. 10.3402/jev.v4.29260 26563735 PMC4643195

[B411] Yue R. Lu S. Luo Y. Zeng J. Liang H. Qin D. (2022). Mesenchymal stem cell-derived exosomal microRNA-182-5p alleviates myocardial ischemia/reperfusion injury by targeting GSDMD in mice. Cell death Discov. 8, 202. 10.1038/s41420-022-00909-6 35422485 PMC9010441

[B412] Zakrzewski W. Dobrzyński M. Szymonowicz M. Rybak Z. (2019). Stem cells: past, present, and future. Stem Cell Res. Ther. 10, 68–22. 10.1186/s13287-019-1165-5 30808416 PMC6390367

[B413] Zebrowska A. Jelonek K. Mondal S. Gawin M. Mrowiec K. Widłak P. (2022). Proteomic and metabolomic profiles of T cell-derived exosomes isolated from human plasma. Cells 11, 1965. 10.3390/cells11121965 35741093 PMC9222142

[B414] Zeng L. Wang H. Shi W. Chen L. Chen T. Chen G. (2021). Aloe derived nanovesicle as a functional carrier for indocyanine green encapsulation and phototherapy. J. Nanobiotechnology 19, 439. 10.1186/s12951-021-01195-7 34930289 PMC8686546

[B415] Zhang K. Zhao X. Chen X. Wei Y. Du W. Wang Y. (2018). Enhanced therapeutic effects of mesenchymal stem cell-derived exosomes with an injectable hydrogel for hindlimb ischemia treatment. INTERFACES 10, 30081–30091. 10.1021/acsami.8b08449 30118197

[B416] Zhang Q. Sun J. Huang Y. Bu S. Guo Y. Gu T. (2019a). Human amniotic epithelial cell-derived exosomes restore ovarian function by transferring microRNAs against apoptosis. Mol. Ther. Nucleic Acids 16, 407–418. 10.1016/j.omtn.2019.03.008 31022607 PMC6479666

[B417] Zhang W. Ou X. Wu X. (2019b). Proteomics profiling of plasma exosomes in epithelial ovarian cancer: a potential role in the coagulation Cascade, diagnosis and prognosis. Int. J. Oncol. 54, 1719–1733. 10.3892/ijo.2019.4742 30864689 PMC6438431

[B418] Zhang L. Li H.-H. Yuan M. Li D. Wang G.-Y. (2020a). Exosomal miR-22-3p derived from peritoneal macrophages enhances proliferation, migration, and invasion of ectopic endometrial stromal cells through regulation of the SIRT1/NF-κB signaling pathway. Eur. Rev. Med. Pharmacol. Sci. 24, 571–580. 10.26355/eurrev_202001_20033 32016958

[B419] Zhang N. Zhu J. Ma Q. Zhao Y. Wang Y. Hu X. (2020b). Exosomes derived from human umbilical cord MSCs rejuvenate aged MSCs and enhance their functions for myocardial repair. Stem Cell Res. Ther. 11, 273–15. 10.1186/s13287-020-01782-9 32641103 PMC7346506

[B420] Zhang X. Liu L. Tang M. Li H. Guo X. Yang X. (2020c). The effects of umbilical cord-derived macrophage exosomes loaded with cisplatin on the growth and drug resistance of ovarian cancer cells. Drug Dev. Ind. Pharm. 46, 1150–1162. 10.1080/03639045.2020.1776320 32482115

[B421] Zhang Y. Deng Z. Lou D. Wang Y. Wang R. Hu R. (2020d). High-efficiency separation of extracellular vesicles from lipoproteins in plasma by agarose gel electrophoresis. Anal. Chem. 92, 7493–7499. 10.1021/acs.analchem.9b05675 32233393

[B422] Zhang L. Wang H. Zhao G. Li N. Wang X. Li Y. (2021a). Anti-Tim4 grafting strongly hydrophilic metal–organic frameworks immunoaffinity flake for high-efficiency capture and separation of exosomes. Anal. Chem. 93, 6534–6543. 10.1021/acs.analchem.1c00528 33851819

[B423] Zhang S. Huang B. Su P. Chang Q. Li P. Song A. (2021b). Concentrated exosomes from menstrual blood-derived stromal cells improves ovarian activity in a rat model of premature ovarian insufficiency. Stem Cell Res. Ther. 12, 178–16. 10.1186/s13287-021-02255-3 33712079 PMC7953711

[B424] Zhang Y. Chang X. Wu D. Deng M. Miao J. Jin Z. (2021c). Down-regulation of exosomal miR-214-3p targeting CCN2 contributes to endometriosis fibrosis and the role of exosomes in the horizontal transfer of miR-214-3p. Reprod. Sci. 28, 715–727. 10.1007/s43032-020-00350-z 33048316

[B425] Zhang M. Wang X. Xia X. Fang X. Zhang T. Huang F. (2022a). Endometrial epithelial cells-derived exosomes deliver microRNA-30c to block the BCL9/Wnt/CD44 signaling and inhibit cell invasion and migration in ovarian endometriosis. Cell Death Discov. 8, 151. 10.1038/s41420-022-00941-6 35368023 PMC8976844

[B426] Zhang W. Liu R. Chen Y. Wang M. Du J. (2022b). Crosstalk between oxidative stress and exosomes. Oxid. Med. Cell. Longev. 2022, 3553617. 10.1155/2022/3553617 36082080 PMC9448575

[B427] Zhang D. Du Q. Li C. Ding C. Chen J. He Y. (2023a). Functionalized human umbilical cord mesenchymal stem cells and injectable HA/Gel hydrogel synergy in endometrial repair and fertility recovery. Acta Biomater. 167, 205–218. 10.1016/j.actbio.2023.06.013 37331615

[B428] Zhang J. Guan M. Ma C. Liu Y. Lv M. Zhang Z. (2023b). Highly effective detection of exosomal miRNAs in plasma using liposome-mediated transfection CRISPR/Cas13a. ACS Sens. 8, 565–575. 10.1021/acssensors.2c01683 36722721

[B429] Zhang Y. Li M. Liu X. Yang W. Dong Q. Wang D. (2023c). A delayed spontaneous second-trimester tubo-abdominal pregnancy diagnosed and managed by laparotomy in a “self-identified” infertile woman, a case report and literature review. BMC Pregnancy Childbirth 23, 511. 10.1186/s12884-023-05793-1 37442982 PMC10347766

[B430] Zhang Y. Liang F. Zhang D. Qi S. Liu Y. (2023d). Metabolites as extracellular vesicle cargo in health, cancer, pleural effusion, and cardiovascular diseases: an emerging field of study to diagnostic and therapeutic purposes. arXiv 157, 114046. 10.1016/j.biopha.2022.114046 36469967

[B431] zhang Y. Yang Y. Ren L. Zhan M. Sun T. Zou Q. (2024). Predicting intercellular communication based on metabolite-related ligand-receptor interactions with MRCLinkdb. BMC Biol. 22, 152. 10.1186/s12915-024-01950-w 38978014 PMC11232326

[B432] Zhang X. Ma L. Liu X. Zhou X. Wang A. Lai Y. (2025). Sustained release of miR-21 carried by mesenchymal stem cell-derived exosomes from GelMA microspheres inhibits ovarian granulosa cell apoptosis in premature ovarian insufficiency. Mater. Today Bio 31, 101469. 10.1016/j.mtbio.2025.101469 39906205 PMC11790500

[B433] Zhao S. Qi W. Zheng J. Tian Y. Qi X. Kong D. (2020). Exosomes derived from adipose mesenchymal stem cells restore functional endometrium in a rat model of intrauterine adhesions. Reprod. Sci. 27, 1266–1275. 10.1007/s43032-019-00112-6 31933162

[B434] Zhao Y. Pan S. Wu X. (2022). Human umbilical cord mesenchymal stem cell-derived exosomes inhibit ovarian granulosa cells inflammatory response through inhibition of NF-κB signaling in polycystic ovary syndrome. J. Reproductive Immunol. 152, 103638. 10.1016/j.jri.2022.103638 35588629

[B435] Zhao M. Li Q. Zhao Y. Zhou H. Yan Y. Kong R.-M. (2024). Dual-aptamer recognition of DNA logic gate sensor-based specific exosomal proteins for ovarian cancer diagnosis. ACS sensors 9, 2540–2549. 10.1021/acssensors.4c00270 38635557

[B436] Zhao X. Liu X. Chen T. Xie H. Li S. Zhang Y. (2025). Fully integrated centrifugal microfluidics for rapid exosome isolation, glycan analysis, and point-of-care diagnosis. ACS nano 19, 8948–8965. 10.1021/acsnano.4c16988 40014808

[B437] Zheng Y.-L. Wang W.-D. Cai P.-Y. Zheng F. zhou Y.-F. Li M.-M. (2022). Stem cell-derived exosomes in the treatment of acute myocardial infarction in preclinical animal models: a meta-analysis of randomized controlled trials. Stem Cell Res. and Ther. 13, 151. 10.1186/s13287-022-02833-z 35395872 PMC8994329

[B438] Zhou X. Kurywchak P. Wolf-Dennen K. Che S. P. Y. Sulakhe D. D’souza M. (2021). Unique somatic variants in DNA from urine exosomes of individuals with bladder cancer. Mol. Ther. Methods and Clin. Dev. 22, 360–376. 10.1016/j.omtm.2021.05.010 34514028 PMC8408559

[B439] Zhou Z. Tu Z. Zhang J. Tan C. Shen X. Wan B. (2022). Follicular fluid-derived exosomal MicroRNA-18b-5p regulates PTEN-Mediated PI3K/Akt/mTOR signaling pathway to inhibit polycystic ovary syndrome development. Mol. Neurobiol. 59, 2520–2531. 10.1007/s12035-021-02714-1 35092573

[B440] Zhou C. Zhang B. Yang Y. Jiang Q. Li T. Gong J. (2023a). Stem cell-derived exosomes: emerging therapeutic opportunities for wound healing. Stem Cell Res. Ther. 14, 107. 10.1186/s13287-023-03345-0 37101197 PMC10134577

[B441] Zhou Y. Deng G. She H. Bai F. Xiang B. Zhou J. (2023b). Polydopamine-coated biomimetic bone scaffolds loaded with exosomes promote osteogenic differentiation of BMSC and bone regeneration. Regen. Ther. 23, 25–36. 10.1016/j.reth.2023.03.005 37063095 PMC10091039

[B442] Zhu X. Badawi M. Pomeroy S. Sutaria D. S. Xie Z. Baek A. (2017). Comprehensive toxicity and immunogenicity studies reveal minimal effects in mice following sustained dosing of extracellular vesicles derived from HEK293T cells. J. Extracell. vesicles 6, 1324730. 10.1080/20013078.2017.1324730 28717420 PMC5505007

[B443] Zhu Q. Cheng L. Deng C. Huang L. Li J. Wang Y. (2021). The genetic source tracking of human urinary exosomes. Proc. Natl. Acad. Sci. 118, e2108876118. 10.1073/pnas.2108876118 34663731 PMC8639375

[B444] Zhu Q. Tang S. Zhu Y. Chen D. Huang J. Lin J. (2022a). Exosomes derived from CTF1-modified bone marrow stem cells promote endometrial regeneration and restore fertility. Front. Bioeng. Biotechnol. 10, 868734. 10.3389/fbioe.2022.868734 35497344 PMC9043110

[B445] Zhu Y.-G. Shi M.-M. Monsel A. Dai C.-X. dong X. Shen H. (2022b). Nebulized exosomes derived from allogenic adipose tissue mesenchymal stromal cells in patients with severe COVID-19: a pilot study. Stem cell Res. and Ther. 13, 220. 10.1186/s13287-022-02900-5 35619189 PMC9135389

[B446] Zhu S. Wang A. Xu W. Hu L. Sun J. Wang X. (2023). The heterogeneity of fibrosis and angiogenesis in endometriosis revealed by single-cell RNA-Sequencing. Biochim. Biophys. Acta. Mol. Basis Dis. 1869, 166602. 10.1016/j.bbadis.2022.166602 36400338

[B447] Zhu X. Li W. Lu M. Shang J. Zhou J. Lin L. (2024). M6A demethylase FTO-Stabilized exosomal circBRCA1 alleviates oxidative stress-induced granulosa cell damage *via* the miR-642a-5p/FOXO1 axis. J. Nanobiotechnology 22, 367. 10.1186/s12951-024-02583-5 38918838 PMC11197183

[B448] zhu X. Lu M. Li W.-X. Lin L. Liu Y. Zhou J. (2025). HuMSCs-derived exosomal YBX1 participates in oxidative damage repair in granulosa cells by stabilizing COX5B mRNA in an m5C-dependent manner. Int. J. Biol. Macromol. 310, 143288. 10.1016/j.ijbiomac.2025.143288 40253045

[B449] Zhuang X. Xiang X. Grizzle W. Sun D. Zhang S. Axtell R. C. (2011). Treatment of brain inflammatory diseases by delivering exosome encapsulated anti-inflammatory drugs from the nasal region to the brain. Mol. Ther. 19, 1769–1779. 10.1038/mt.2011.164 21915101 PMC3188748

[B450] Zou W. Tang L. Wallis C. (2025). “my body is betraying me”: exploring the stigma and coping strategies for infertility among women across ethnic and racial groups. Health Commun., 1–12. 10.1080/10410236.2025.2470984 39991807

[B451] Zuo R. Liu M. Wang Y. Li J. Wang W. Wu J. (2019). BM-MSC-derived exosomes alleviate radiation-induced bone loss by restoring the function of recipient BM-MSCs and activating Wnt/β-catenin signaling. Stem cell Res. and Ther. 10, 30. 10.1186/s13287-018-1121-9 30646958 PMC6334443

