# Supplementary figures and images for: From biomolecules to breakthroughs: exosomes as next-generation theranostics in female infertility
Source: Front Cell Dev Biol. 2025 Oct 1;13:1605174. doi: 10.3389/fcell.2025.1605174 (PMC12521274; doi:10.3389/fcell.2025.1605174)

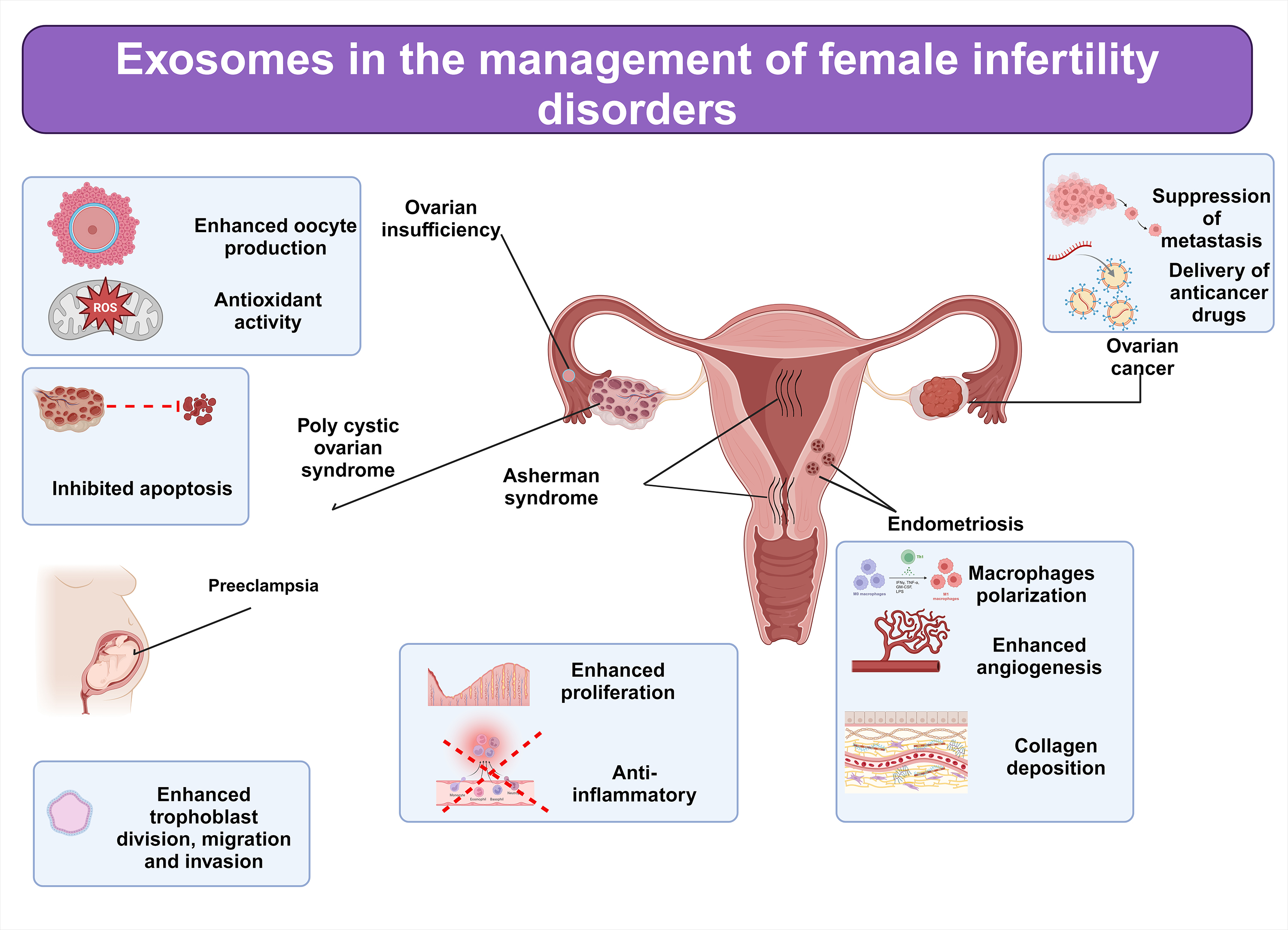

Supplement: Supplementary file 1 [file Image1.jpeg]
